# Development of cleaved amplified polymorphic sequence marker for powdery mildew resistance in Korean malting barley using QTL-seq

**DOI:** 10.3389/fpls.2025.1596811

**Published:** 2025-05-12

**Authors:** Jin-Cheon Park, Young-Mi Yoon, Chang-Hyun Lee, On-Sook Hur, Sang-Min Kim

**Affiliations:** ^1^ Winter Crop Research Division, Department of Crop Science, National Institute of Crop and Food Science, Rural Development Administration, Wanju, Republic of Korea; ^2^ Crop Environment Research Division, Department of Crop Science, National Institute of Crop and Food Science, Rural Development Administration, Wanju, Republic of Korea

**Keywords:** malting barley, powdery mildew, resistance, QTL-seq, molecular marker, candidate gene

## Abstract

**Introduction:**

Powdery mildew (PM) caused by *Blumeria graminis* f.sp. *hordei* is a major fungal disease affecting barley (*Hordeum vulgare* L.). The most effective approach to controlling this disease is the development of resistant cultivars. In this study, we investigated the genomic regions associated with PM resistance by performing quantitative loci sequencing (QTL-seq) twice using the parental lines ‘Hopum’ (susceptible) and ‘Jeonju 182’ (resistant) as reference genomes.

**Methods:**

This study was conducted from 2022 to 2024 at the National Institute of Agricultural Sciences in Wanju, Republic of Korea. We conducted artificial crossing, genomic DNA extraction, phenotypic evaluation, QTL-seq analysis, and cleaved amplified polymorphic sequence (CAPS) marker development. Candidate gene expression was analyzed using real-time quantitative reverse transcription PCR.

**Results:**

A total of 2,130 common variants were identified in two regions of chromosome 1H (6,940,595–18,008,713 bp and 19,363,700–20,551,018 bp). Twenty-one non-synonymous single nucleotide polymorphisms among these variants were used to develop CAPS markers, which were validated in an F_2_ population and malting barley cultivars. The PMC_75 marker, which is annotated as *HORVU.MOREX.r3.1HG0005790*, showed a strong association with resistance and was highly expressed in ‘Jeonju 182.’ This marker is associated with a Clathrin Assembly Protein, which is involved in vesicle formation and intracellular trafficking, processes essential for cellular signaling and defense responses.

**Conclusion:**

The development of the CAPS marker (PMC_75) provides a valuable tool for marker-assisted selection in breeding PM-resistant malting barley, improving breeding efficiency, and accelerating the development of resistant cultivars.

## 1 Introduction

Barley (*Hordeum vulgare* L.) is the fourth most widely cultivated cereal crop worldwide, following rice, wheat, and maize (FAOSTAT, 2021). It is primarily used for animal feed, followed by malting and brewing, with a smaller proportion used for human consumption (Ross et al., 2023). In Korea, barley is considered the second most important food crop after rice (Zhou, 2009). It is used for both food and industrial purposes, particularly in the preparation of mixed-grain rice and traditional dishes, as well as in beer production. Malting barley, which is primarily used for brewing, requires stable grain yield and the production of high-quality grain with uniform and well-filled kernels. In 2024, the total cultivation area of barley in Korea was 23,298 hectares, of which 5,468 hectares were devoted to malting barley production (KOSIS, 2024). With the rise of local craft beer production, malting barley is gaining increasing attention as a key raw material.

Cultivated barley originated approximately 10,000 years ago in the Fertile Crescent through the domestication of its wild progenitor, *Hordeum* sp*ontaneum* C. Koch (Badr et al., 2000). Barley is classified into two- and six-row types based on spike morphology and into hulled and hulless types depending on the presence of a hull. Two-row hulled barley is predominantly used for malting, requiring stable cultivation practices to ensure high-quality raw grain production.

Powdery mildew (PM) is one of the most common diseases affecting barley and is caused by the obligate biotrophic fungus *Blumeria graminis* f.sp. *hordei*, which thrives in warm temperatures and high humidity conditions during the growing season. The pathogen forms white conidia on the leaf surface and absorbs plant nutrients using invertase, a sugar-degrading enzyme, thereby restricting nutrient transport to the grain. This results in a reduced seed set and lower yields due to decreased photosynthetic efficiency caused by impaired CO_2_ exchange through stomata (Akhkha, 2008; Swarbrick et al., 2006). Powdery mildew (PM) infection can result in yield losses that vary depending on climate conditions and barley cultivars, with average yield losses reported to range from 10-20%, and in some cases, exceeding 50% (Tratwal and Weber, 2006). In Korea, powdery mildew frequently occurs under warm and humid spring conditions and has been identified as a potential threat to barley production, particularly under recent climate trends (Jeong et al., 2024). This disease negatively affects grain filling and kernel uniformity, which are critical for meeting the quality standards required for malting (Oser, 2015; Sánchez-Martín et al., 2018). As a result, the continued occurrence of powdery mildew poses a serious constraint on the stable production of high-quality malting barley and may ultimately undermine the competitiveness of the domestic brewing industry.

PM is a rapidly spreading disease due to airborne spore transmission and requires a living host for infection and progression. When susceptible barley cultivars are cultivated, PM spores cover the leaf surface, inhibiting photosynthesis and leading to reduced plant growth and yield (Ellwood et al., 2024). Barley plants infected with PM suffer the most severe damage at GS 22 (early tillering stage) and GS 31 (stem elongation stage), and early disease onset reportedly significantly impacts plant growth and grain filling (Agostinetto et al., 2014). According to studies by Glawe (2008) and Zhang et al. (2005), *B. graminis* f.sp. *hordei* exhibits peak invasion activity within 24–48 h (day 2) post-infection, during which haustoria formation occurs, facilitating nutrient absorption and host-pathogen interactions. Considering the progression of pathogen infection, identifying critical early time points is necessary for assessing gene expression associated with host resistance.

Several resistance genes against PM, including *Mla*, *Mlk*, and *Mlg*, have been identified; however, their effectiveness varies depending on the host cultivar. In contrast, *Mlo* has been shown to confer broad-spectrum resistance across different barley genotypes (Dreiseitl, 2024). Fungicide application is a crucial strategy for managing fungal diseases and ensuring high grain quality; however, excessive fungicide use raises concerns about the development of resistant fungal strains. Therefore, developing resistant cultivars by incorporating resistance genes is the most cost-effective strategy for minimizing yield losses caused by PM (Yin et al., 2023).

Barley, a diploid species (2n=2x=14) with a genome size of 5.1 Gb, is one of the crops with the largest genomes among major crops, with over 80% of its nucleotide sequence consisting of simple repeats (Ariyadasa et al., 2014). These repetitive sequences have been a major challenge for genome assembly in large-genome crops. However, since the assembly of a high-resolution reference genome for the six-row American barley cultivar ‘Morex’ by an international research team in 2017, various genomic analyses have been conducted to explore variants associated with target traits (Beier et al., 2017; Mascher et al., 2017).

Quantitative trait loci sequencing (QTL-seq) is a genetic analysis method based on whole-genome sequencing (WGS) that combines bulked segregant analysis (BSA) with next-generation sequencing technology (Wu et al., 2019). BSA is a powerful approach for identifying major QTLs or candidate genes by comparing individuals with distinct phenotypes (de la Fuente Cantó and Vigouroux, 2022; Zou et al., 2016). In this method, F_1_ hybrids are generated through artificial crosses between parental lines, resulting in the development of an F_2_ segregating population. Phenotypic screening is performed on the F_2_ individuals, and DNA from individuals exhibiting the two extreme phenotypes is pooled into bulk samples for sequencing. This enables the rapid and cost-effective identification of target traits in early generations. Due to these advantages, QTL-seq has been widely applied to various traits in different crops, including grain number per panicle in rice (*Oryza sativa*) (Ariharasutharsan et al., 2024), cytoplasmic male sterility in maize (*Zea mays*) (Zheng et al., 2020), plant height in rapeseed (*Brassica napus*) (Dong et al., 2021), heading time in wheat (*Triticum aestivum*) (Komura et al., 2024), protein content in peanut (*Arachis hypogaea*) (Chen et al., 2024), and fruit skin color in cucumber (*Cucumis sativus*) (Kishor et al., 2021). However, research utilizing QTL-seq for identifying traits associated with PM resistance in barley remains limited.

Developing cultivars incorporating these key target traits requires introgressing the associated genes through crosses with genetic resources that possess the desired traits. However, barley breeding in Korea predominantly relies on conventional breeding methods, where trait selection is performed at advanced generations through phenotypic evaluation. This approach poses challenges, including the potential loss of valuable genetic resources in early generations and an extended breeding cycle. Therefore, this study aimed to develop molecular markers associated with powdery mildew (PM) resistance and utilize them for marker-assisted selection (MAS) to enable rapid and precise selection from early generations, ultimately shortening the breeding cycle and improving breeding efficiency. This study was conducted in the context of a limited number of prior studies on the development of molecular markers for PM resistance in Korean barley, and the findings are expected to serve as a foundation for future applications of early-generation selection technologies and the development of resistant cultivars using diverse genetic resources.

## 2 Materials and methods

### 2.1 Identification of PM pathogen

The barley PM pathogen was collected from infected plants at the Jeju Agricultural Research Institute in March 2022, and genomic DNA (gDNA) was extracted from fungal hyphae formed on the upper surface of infected leaves using a DNeasy Plant Kit (Qiagen, Hilden, Germany) following the manufacturer’s instructions. The extracted DNA was stored at –80 °C until further analysis. PCR amplification was performed using the ITS5 (GGAAGTAAAAGTCGTAACAAGG) and ITS4 (TCCTCCGCTTATTGATATGC) primers under the following conditions: an initial pre-denaturation at 94°C for 3 min, followed by 35 cycles of denaturation at 94°C for 30 s, annealing at 53°C for 30 s, and extension at 72°C for 1 min, with a final elongation step at 72°C for 10 min. To obtain accurate sequence information for the ITS region, additional sequencing was conducted using the ITS1 (TCCGTAGGTGAACCTGCGG) and ITS2 (GCTGCGTTCTTCATCGATGC) primers, and sequencing was performed outsourced to Macrogen (Seoul, Korea). The obtained sequences were analyzed using BLASTN in the National Center for Biotechnology Information (NCBI) database for sequence comparison, and the 5.8S rRNA sequences of the identified isolates were further analyzed phylogenetically using MEGA 11 software (Tamura et al., 2021). A neighbor-joining method with 1,000 bootstrap replications was used to compare the evolutionary relationships between the identified isolate and other PM-causing fungal species registered in the GenBank database.

### 2.2 Plant materials

To develop markers associated with PM resistance in barley, an artificial cross was performed using ‘Hopum,’ a susceptible malting barley cultivar (Hyun et al., 2006a), as the female parent and ‘Jeonju 182,’ a resistant cultivar, as the male parent. The resulting F_1_ hybrids were obtained and subsequently grown to produce an F_2_ population, allowing for genetic segregation.

### 2.3 Phenotypic evaluation and inheritance pattern

PM resistance was evaluated in 10 individuals each from the susceptible (‘Hopum’) and resistant (‘Jeonju 182’) parents, 15 F_1_ hybrids (‘Hopum’ × ‘Jeonju 182’), and 200 randomly selected F_2_ individuals. The assessment was conducted based on the infection type (IT) scale proposed by Mains and Dietz (1930), categorizing disease severity from IT0 to IT4. To ensure consistent pathogen proliferation, all plants were maintained for 10 days in a growth chamber with controlled temperature and humidity. IT0 represents a highly resistant stage where the leaves remain free of visible infection. IT1 is classified as resistant, where the obligate biotrophic *B. graminis* f.sp. *hordei* begins to colonize the leaf surface but is suppressed by a hypersensitive response (HR), leading to localized necrosis. IT2 indicates moderate susceptibility, characterized by necrotic spots with little conidia formation. IT3 and IT4 are classified as susceptible stages, with white conidial spores appearing in small (IT3) or large (IT4) amounts without necrotic spot formation. To determine the inheritance pattern of PM resistance, a chi-square (χ²) test was performed. The inheritance pattern was analyzed based on the phenotypic evaluation of the parental lines (‘Hopum’ and ‘Jeonju 182’), 15 F_1_ hybrids, and 200 F_2_ individuals. Resistance and susceptibility were classified according to the results of the seedling-stage phenotypic assessment, and the observed segregation ratio in the F_2_ population was compared with the expected Mendelian ratios to infer the genetic basis of resistance.

### 2.4 gDNA extraction

A total of 42 DNA samples were extracted from ‘Hopum’ (susceptible parent), ‘Jeonju 182’ (resistant parent), 20 susceptible F_2_ individuals, and 20 resistant F_2_ individuals for QTL-seq analysis. Leaf samples were collected at the 2-leaf stage from all 200 F_2_ individuals before transferring them to the growth chamber for seedling-stage PM phenotyping. Based on the phenotyping results, 20 resistant and 20 susceptible individuals were selected for gDNA extraction. gDNA was extracted using the HiGene™ Genomic DNA Prep Kit (BIOFACT, Daejeon, Korea) following the manufacturer’s protocol. The quantity and purity of the extracted DNA were assessed using a NanoDrop 1000 spectrophotometer (Thermo Fisher Scientific, Waltham, MA, USA) and confirmed using electrophoresis. Equal amounts of gDNA from the 20 susceptible F_2_ individuals were pooled to create the susceptible DNA pool (HvPM_S20), while DNA from the 20 resistant F_2_ individuals was pooled to create the resistant DNA pool (HvPM_R20).

### 2.5 Whole-genome re-sequencing and QTL-seq

The quality and quantity of the extracted gDNA were measured using an Agilent 2200 TapeStation (Agilent Technologies, Santa Clara, CA, USA). A paired-end sequencing library was then constructed using a TruSeq DNA PCR-Free Kit (Illumina, San Diego, CA, USA), and WGS was performed on an Illumina NovaSeq platform (Illumina). To ensure high-quality sequencing data, raw reads were preprocessed using Trimmomatic, which filtered out low-quality reads and adapter sequences. QTL-seq analysis was performed using the QTL-seq program (version 2.2.2, https://github.com/YuSugihara/QTL-seq) following the method described by Takagi et al. (2013). Briefly, low-quality and duplicate reads were removed from the raw sequencing data using Trimmomatic (version 0.39, Bolger et al., 2014). The reference genome used for alignment was the *Hordeum vulgare* Morex V3 genome sequence, obtained from Phytozome13 (https://phytozome-next.jgi.doe.gov/info/HvulgareMorex_V3, Mascher et al., 2021). First, high-quality sequencing data from ‘Hopum’ and the two pooled DNA samples were processed using the QTL-seq program. The mean single nucleotide polymorphism (SNP) index and Δ(SNP index) values were calculated within genomic intervals using a sliding window of 2 Mb with 100-kb increments, and the results were visualized as SNP index plots along the chromosomes. Candidate genomic regions associated with the trait were identified based on the sliding-window plot. Genomic regions with an average Δ(SNP index) significantly higher than that of the surrounding regions and *p* < 0.01 were considered candidate QTLs. Variants within the candidate QTL regions were extracted from the variant calling file (VCF), and only those satisfying *p* < 0.01 were selected. In the second analysis, high-quality sequencing data from ‘Jeonju 182’ and the two pooled DNA samples were processed using the same QTL-seq pipeline to identify candidate QTL regions. Significant variants (*p* < 0.01) were selected following the same criteria as in the first analysis. Finally, common significant variants (*p* < 0.01) identified in both QTL-seq analyses were selected for further investigation.

### 2.6 Variant analysis and selection

The genomic locations of the identified variants were analyzed using the SnpEff program (version 5.0e, Cingolani et al., 2012). Variants located within gene regions, including the 5′ untranslated region (UTR), 3′ UTR, introns, and exons, were classified as genic, while those located outside gene regions were categorized as intergenic. Additionally, variants within coding sequences (CDSs) were further classified as synonymous or non-synonymous. Functional information on the genes containing these variants was obtained from the gene description file provided in the *Hordeum vulgare* Morex V3 genome database on Phytozome13. The identified variants were further filtered based on the following criteria to refine candidate variants: (1) only variants that differed between the two pooled DNA samples (susceptible and resistant) were selected; (2) only variants that differed between the parental lines (‘Hopum’ and ‘Jeonju 182’) were retained; (3) only variants that were homozygous in the susceptible pooled DNA sample were considered; and (4) optionally, variants that caused changes in the protein sequence of the associated genes were prioritized for selection.

### 2.7 Development of molecular marker

The flanking sequences (± 500 bp) surrounding the selected variant regions were extracted from the *Hordeum vulgare* Morex V3 reference genome. Forward and reverse primers were designed to amplify these regions for PCR-based cleaved amplified polymorphic sequence (CAPS) marker development using CAPS-finder.pl (https://github.com/mfcovington/CAPS-finder/blob/master/CAPS-finder.pl). The primer design parameters were set as follows: primer size of 19–22 mer, maximum GC content of 59%, annealing temperature (Tm) range of 55–62°C, and amplicon size of 300–700 bp. The specificity of the designed primers was verified by conducting BLASTN analysis against the reference genome with a cutoff E-value of 1e-5.

### 2.8 RNA extraction and cDNA synthesis

Leaf samples from ‘Hopum’ (susceptible) and ‘Jeonju 182’ (resistant) were collected at three time points: day 0 (before inoculation), day 2 (when the pathogen began invading and spores first appeared on susceptible plants), and day 4 (when infection had spread further). The collected samples were ground into a fine powder using a mortar and pestle in liquid nitrogen with RNA lysis buffer, and the resulting suspension was stored at -80°C until further use. Total RNA was extracted using a Quick-RNA Miniprep Kit (Zymo Research, Irvine, CA, USA) according to the manufacturer’s instructions. The quality and quantity of the extracted RNA were assessed using a DeNovix DS-11+ Spectrophotometer (DeNovix, Wilmington, DE, USA) and further validated using 1.2% (v/v) agarose gel electrophoresis. For cDNA synthesis, 2 μg of RNA from each sample was used in the following reaction. RNA (2 μg) and 1 μL oligo(dT) (100 pmol/μL) were mixed, and DEPC-treated distilled water was added to a final volume of 15.5 μL. Next, the mixture was incubated at 65°C for 5 min to denature secondary RNA structures. Thereafter, 2 μL 10X reaction buffer, 1 μL dNTP mix (10 mM each dATP, dGTP, dCTP, dTTP), 0.5 μL RNase inhibitor (20 U/μL), and 1 μL reverse transcriptase (200 U/μL; Enzynomics, Daejeon, Korea) were added to reach a final volume of 20 μL. Subsequently, the reaction mixture was incubated at 42°C for 90 min, followed by enzyme inactivation at 70°C for 15 min. To verify cDNA synthesis quality, PCR amplification was performed using β-TUB6 gene primers (Chen et al., 2018), and the amplification products were confirmed using agarose gel electrophoresis. For PCR, a 20-μL reaction mixture was prepared using 50 ng cDNA as the template, 0.8 μL gene-specific primer mix (10 pmol/μL for both forward and reverse primers), 10 μL 2X Taq mix (DS Biotech, China), and sterile distilled water. PCR conditions comprised an initial denaturation at 94°C for 5 min, followed by 30 cycles of 94°C for 15 s, 55°C for 20 s, and 72°C for 30 s.

### 2.9 Real-time quantitative reverse transcription PCR for candidate gene

Real-time PCR was performed on independently synthesized cDNA templates from ‘Hopum’ (susceptible) and ‘Jeonju 182’ (resistant) without technical replicates. The reaction mixture consisted of 4 μL cDNA (12.5 ng/μL), 0.8 μL gene-specific primers (10 pmol/μL each for forward and reverse), 10 μL 2× TOPreal™ SYBR Green qPCR PreMIX (Enzynomics), and 5.2 μL sterile distilled water, bringing the final volume to 20 μL. Amplification was conducted using a LightCycler^®^ 480 II real-time PCR system (Roche Diagnostics GmbH, Mannheim, Germany) under the following conditions: initial denaturation at 95°C for 5 min, followed by 65 cycles of 95°C for 15 s, 55°C for 20 s, and 72°C for 30 s. The relative gene expression levels were analyzed using the comparative Ct method (Livak and Schmittgen, 2001), with values calculated as 2^-(Ct target gene - Ct housekeeping gene)^.

### 2.10 Statistical analysis

To examine the segregation pattern of PM resistance in the F_2_ malting barley population, statistical analysis was performed using the R software package (R Foundation for Statistical Computing, Vienna, Austria). Based on the phenotypic evaluation results, the goodness-of-fit of the observed segregation ratio to the expected Mendelian inheritance pattern was tested using the chi-square (χ²) test.

## 3 Results

### 3.1 Pathogen isolation and identification

**Figure 1:**
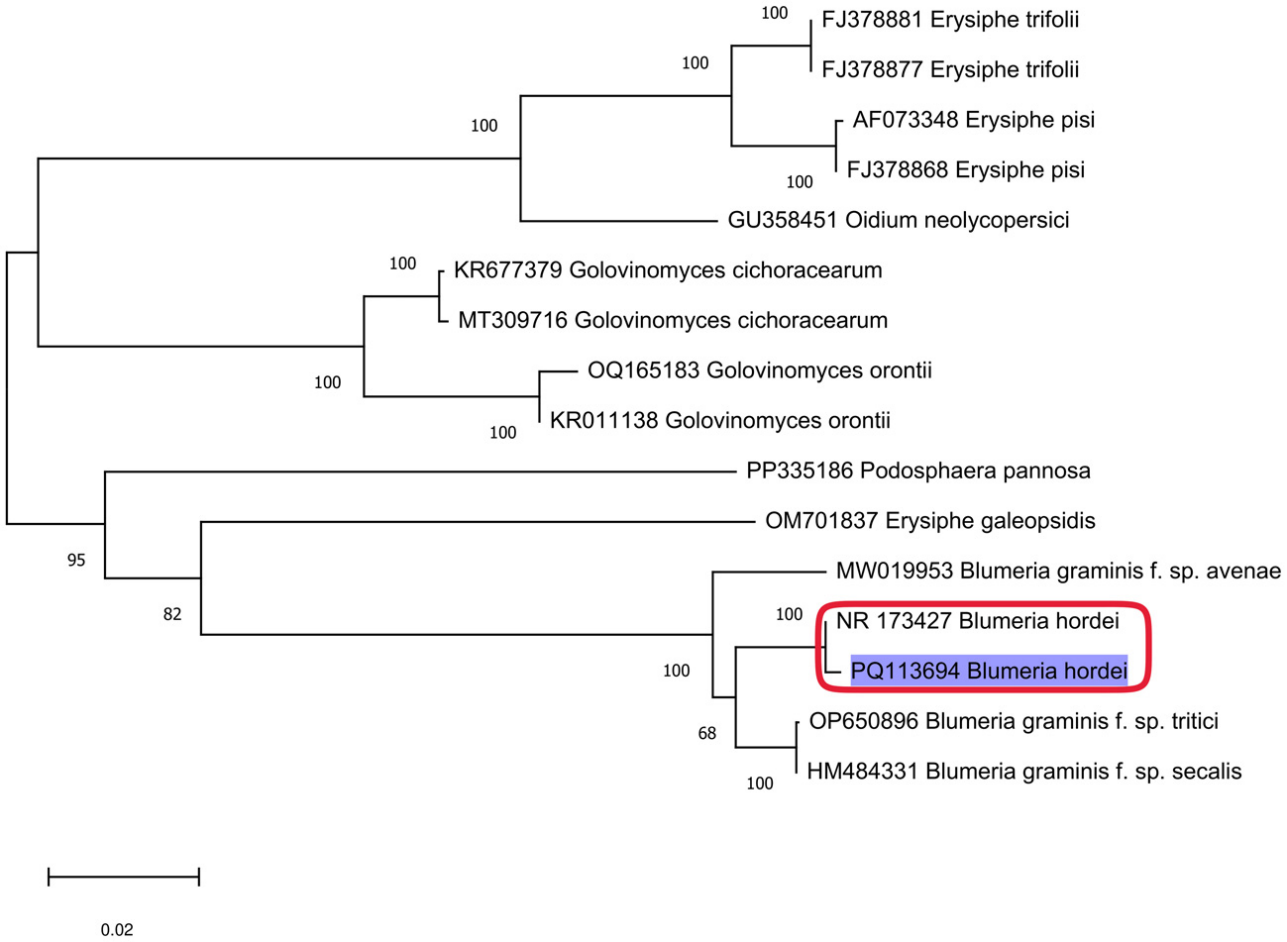
Phylogenic comparison of sequences of the ITS region and rDNA sequences using MEGA 11. Bootstrap support values for 1,000 replicates were calculated using the neighbor-joining method.

PM in barley was isolated from white spores obtained from susceptible seedlings at the early growth stage. To identify the PM pathogen, the extracted gDNA was sequenced and analyzed using NCBI BLASTN, revealing over 99% homology with NR173427, which is registered as *B. hordei*. The nucleotide sequence (553 bp) of the ITS region (5.8S rRNA) of the PM pathogen used in this analysis has been deposited in the NCBI GenBank database under accession number PQ113694. A phylogenetic tree was constructed using MEGA 11, and the ITS sequence of the barley PM pathogen clustered within the same clade as *B. hordei* but as a distinct clade from other *Blumeria* species infecting different hosts, such as wheat (*tritici*), rye (*secalis*), and oat (*avenae*). Additionally, it was distinguished from other PM pathogens belonging to the genera *Erysiphe*, *Oidium*, *Golovinomyces*, and *Podosphaera*, which infect other crops (**Figure 1**).

### 3.2 Phenotypic evaluation and genetic inheritance of PM resistance

**Figure 2:**
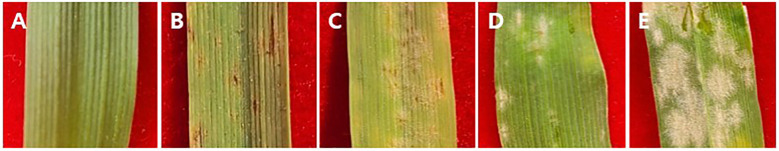
Classification of infection types (ITs) based on powdery mildew severity. **(A)** IT0: highly resistant, **(B)** IT1: resistant, **(C)** IT2: moderately susceptible, **(D)** IT3: susceptible, **(E)** IT4: highly susceptible.

Seedling evaluation was conducted using seeds from 200 randomly selected F_2_ individuals in a controlled growth chamber. The plants were classified into ITs as follows: IT0 (85 individuals), IT1 (62), IT2 (26), IT3 (11), and IT4 (16) (**Figure 2**). When categorized into resistance (IT0-1) and susceptibility (IT2-4) groups, 147 individuals exhibited resistance, whereas 53 showed susceptibility. These results suggest that resistance to PM is controlled by a single dominant gene (**Table 1**). Phenotypic evaluation of the parental lines, F_1_ hybrids, and F_2_ individuals confirmed that all susceptible parental plants were susceptible, all resistant parental plants were resistant, and all F_1_ hybrids exhibited resistance, indicating a dominant inheritance pattern. Chi-square analysis yielded a χ² value of 0.16667 and a *p*-value of 0.6831, which was not statistically significant, confirming the expected 3:1 segregation ratio. Based on these findings, PM resistance was conclusively determined to be governed by a single dominant gene.

**Table 1:** Segregation analysis of powdery mildew resistance in malting barley.

Name	Population	Plant Number	Phenotype of powdery mildew				
			Resistance	Susceptible	Expected	χ^2^	p^*^
Hopum	P1	10	0	10	–	–	–
Jeonju182	P2	10	10	0	–	–	–
Hopum/Jeonju182	F_1_	15	15	0	–	–	–
Hopum/Jeonju182	F_2_	200	147	53	3:1	0.16667	0.6831

^*^ *p-value(>0.05)*, not significant.

### 3.3 BSA and WGS

**Figure 3:**
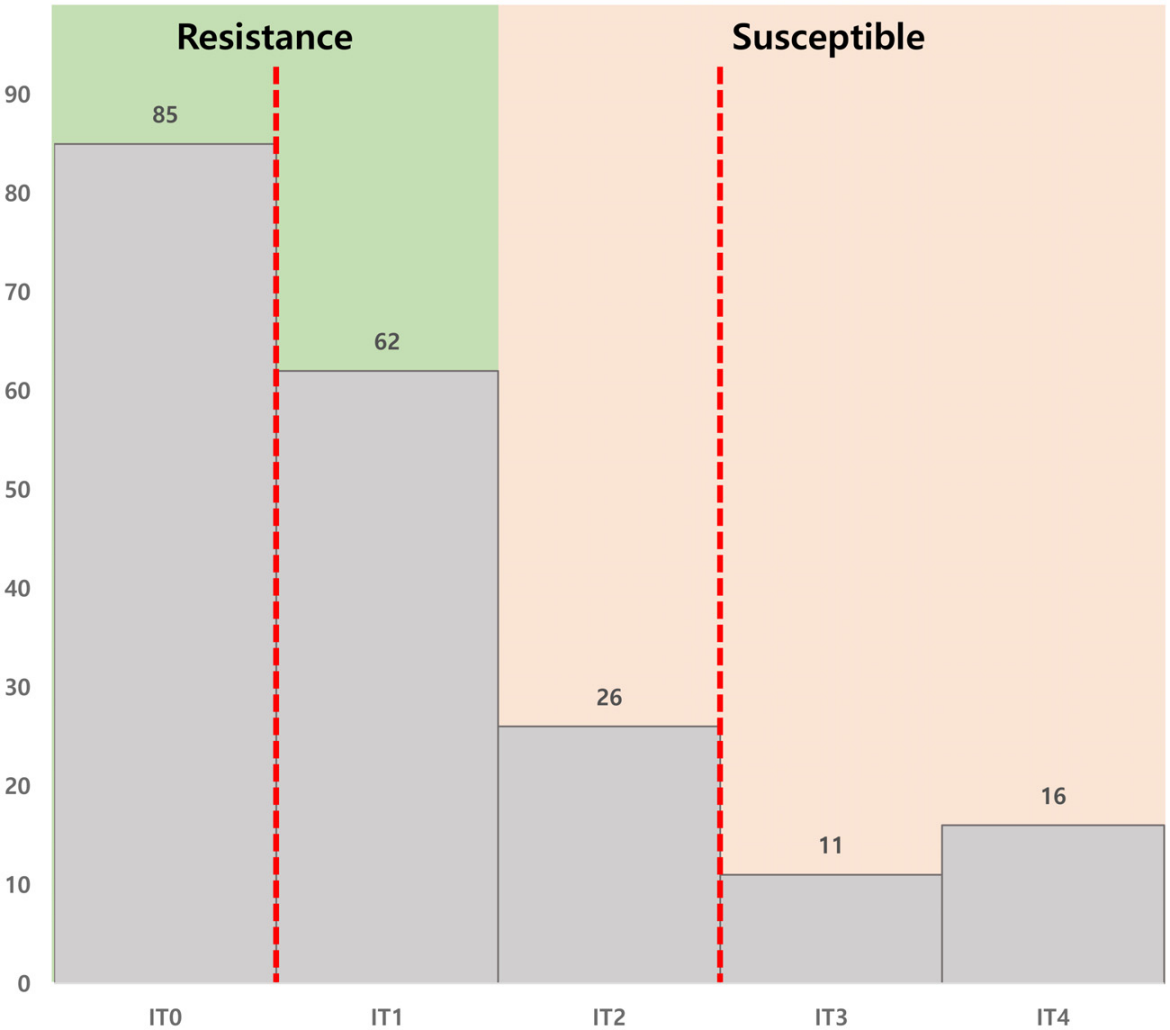
Seedling test results for powdery mildew resistance. For trait evaluation, IT0–1 was classified as resistant to powdery mildew, whereas IT2–4 was classified as susceptible.

A total of 20 susceptible individuals, comprising 16 IT4 individuals and 4 IT3 individuals, were selected. For the resistant group, 20 individuals were randomly selected from 85 IT0 plants (**Figure 3**). To perform BSA, DNA was extracted from the parental lines, ‘Hopum’ (susceptible) and ‘Jeonju 182’ (resistant), as well as from the 20 susceptible (IT3-4) and 20 resistant (IT0) individuals to prepare samples for QTL-seq analysis.

To conduct genetic analysis, the DNA of 20 susceptible individuals was pooled in equal amounts to create the bulk DNA sample HvPM_S20, while the DNA of 20 resistant individuals was pooled similarly to form HvPM_R20. Additionally, WGS was performed, including that of the two parental lines, ‘Hopum’ (susceptible) and ‘Jeonju 182’ (resistant), to identify genetic variations through QTL-seq analysis. The sequencing results yielded 712,327,018 reads (106,208,374,312 bp) for ‘Hopum,’ 722,813,670 reads (107,852,063,420 bp) for ‘Jeonju 182,’ 740,374,466 reads (110,458,639,607 bp) for *HvPM_S20*, and 780,896,672 reads (109,255,275,105 bp) for *HvPM_R20*. The processed sequencing data covered approximately 25× the reference genome, with over 90% of the reads classified as high-quality (**Table 2**).

**Table 2:** Summary of whole-genome re-sequencing data used for QTL-seq analysis.

Sample	Raw data		Trimmed data			Coverage (X)^*^
	Reads	Read Length (bp)	Reads	Read Length (bp)	%	
Hopum	762,636,918	115,158,174,618	712,327,018	106,208,374,312	92.23	25.13
Jeonju182	780,753,646	117,893,800,546	722,813,670	107,852,063,420	91.48	25.52
HvPM_S20	788,764,476	119,103,435,876	740,374,466	110,458,639,607	92.74	26.14
HvPM_R20	780,896,672	117,915,397,472	732,048,160	109,255,275,105	92.66	25.86

^*^Coverage(X): Trimmed data relative to the barley genome size (4,225,605,719 bp).

### 3.4 QTL-seq analysis

Using the QTL-seq program, variations identified in two comparative combinations against the reference genome were analyzed. In the first combination (Hopum-HvPM_S20 vs. HvPM_R20), a total of 10,245,336 variants were detected in ‘Hopum,’ 10,911,858 in HvPM_S20, and 10,806,815 in HvPM_R20, with 1,838,816 variants being statistically significant (**Supplementary Table 1**). In the second combination (Jeonju 182-HvPM_S20 vs HvPM_R20), 8,684,920 variants were detected in ‘Jeonju 182,’ 10,686,283 in HvPM_S20, and 10,649,264 in HvPM_R20, with 1,958,916 variants being statistically significant (**Supplementary Table 2**).

To identify genomic regions associated with PM resistance in malting barley, the SNP index was calculated between the PM-susceptible and -resistant pools. For this analysis, the reference genome Morex was used, and the sequencing data from the two QTL-seq combinations were compared. An SNP index of 0 indicated sequences detected only in the susceptible pool, while an index of 1 represented sequences detected only in the resistant pool. To assess the average SNP index across genomic regions, a sliding window approach with 2-Mb window and 100-kb step sizes was applied. Based on this, SNP index distribution plots for all chromosomes were generated for both susceptible and resistant groups (**Supplementary Figures 1**, **2**). Additionally, Δ(SNP index) values were calculated to compare the SNP index differences between the two groups, and they were visualized with statistical confidence intervals mapped onto the reference genome chromosomes (**Supplementary Figures 3**, **4**).

**Figure 4:**
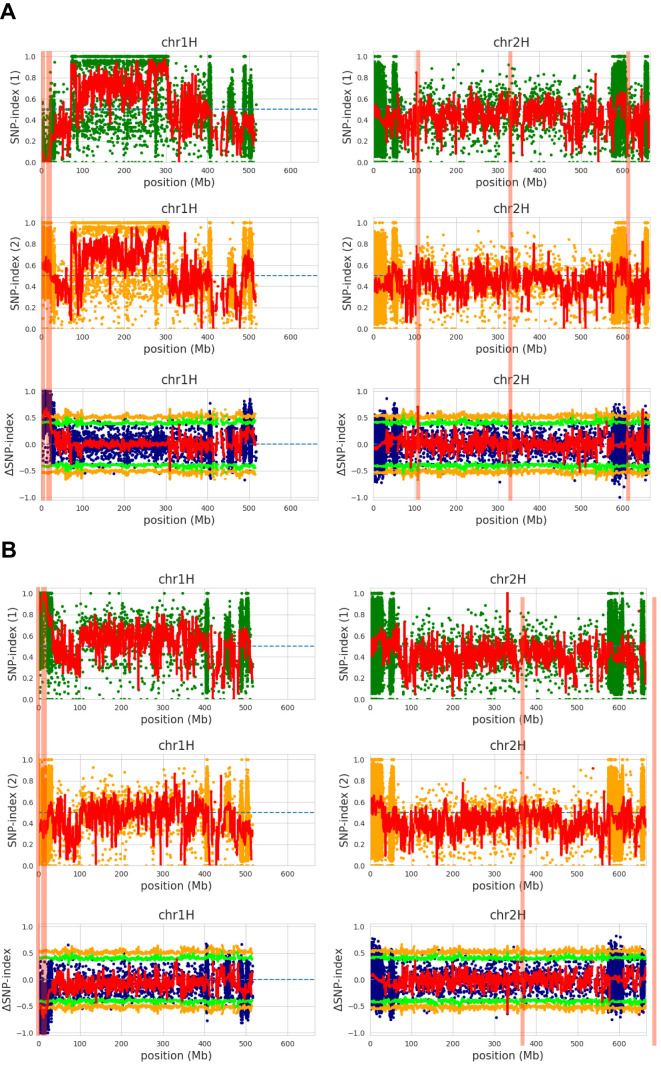
ΔSNP index plots (**A**; Hopum-S20 vs R20, **B**; Jeonju182-S20 vs R20) between bulk1 and bulk2 in chr1H and 2H. SNP index plots of bulk1 (top) and bulk2 (next to the top), ΔSNP-index plot (next to the bottom) with statistical confidence intervals under the null hypothesis of no QTLs (green, *p* < 0.05; orange, *p <* 0.01). The significant genomic regions with *p* < 0.01 are highlighted by the red shaded bar.

Following the principles of QTL-seq analysis, significant genomic regions were identified at *p* < 0.01. In the first combination, major QTL candidate regions associated with PM resistance were detected on chromosome 1 at two loci (3.5, 6.8–21.2Mb) and on chromosome 2 at three loci (106.9–108.6, 330.9–331.3 and 647.4–648.4 Mb). Notably, one candidate QTL region on chromosome 1 was detected as a single peak, and a detailed analysis of the ±2 Mb region (1.5–5.5 Mb) around this locus was conducted. In the second combination, major QTL candidate regions were identified on chromosome 1 at two loci (6.1-6.5, 6.9–20.6 Mb) and on chromosome 2 at two loci (331.2–331.3 and 646.9–648.8 Mb) (**Figure 4**).

In the significant genomic region of chromosome 1 (99% confidence interval), the Δ(SNP index) values ranged from 0.5187 to 0.6785 in the first combination and from 0.5324 to 0.7184 in the second combination. On chromosome 2, the Δ(SNP index) values ranged from 0.6364 to 0.7 in the first combination and from 0.6364 to 0.6515 in the second combination (**Supplementary Tables 3**, **4**).

Across the two analysis combinations, three QTL candidate regions meeting the *p* < 0.01 threshold were identified: 6.9–20.6 Mb on chromosome 1 and 331.2–331.3 Mb and 647.4–648.4 Mb on chromosome 2 (**Supplementary Table 5**). A total of 2,144 common variants were found within these candidate QTL regions, including 1,808 SNPs and 336 insertions/deletions (InDels). Among these, the final set of 2,130 variants on chromosome 1 was confirmed after filtering for variants that were homozygous or heterozygous in the resistant parent and resistant bulk samples (**Supplementary Table 6**).

### 3.5 Identification of variant types within candidate QTL regions

Among the 2,130 variants identified within the candidate QTL regions, variants that were not detected in the BLASTN results against the reference sequence (‘Morex’) or had non-unique flanking sequences were excluded. As a result, a final set of 134 CAPS markers was developed for PCR-based gel analysis (**Supplementary Table 7**). Among these 134 markers, most (75) were located within introns. For SNP variations, 21 were non-synonymous SNPs that caused amino acid changes, while 26 were synonymous SNPs that did not affect the amino acid sequence. Additionally, 1 InDel within a coding region led to codon changes, and 11 variants were located in UTRs. In this study, marker validation focused on 21 non-synonymous SNPs, as these variants were located within CDSs and were likely to affect protein function through amino acid changes (**Table 3**).

**Table 3:** Primers for genotyping 21 non-synonymous single nucleotide polymorphism-based cleaved amplified polymorphic sequence markers.

Marker	Chr	Position	Ref	Alt	Sequence	Amplicon size (S/R)	Restriction enzyme
PMC_4	1H	7842041	A	G	(F) GTTGAATCTGGAATTCTGAAGC (R) CAAGAAGAGAACCTCCACTCAC	159,168/ 327	BstUI
PMC_23	1H	8870292	C	T	(F) CCACTTGAGGAGAACAACTTTC (R) CATCATATTCAATCCAATGCTG	90,214,216/ 214,306	NlaIII
PMC_28	1H	8938251	A	G	(F) ACGAGTTCAAGACCACCTACC (R) TTTCGGAATATTGAAGCTGTTT	8,12,39,70,108,38/ 8,12,39,108,455	HpaII
PMC_29	1H	8951116	G	A	(F) GAAGGTCCTCTTATGCTGTACG (R) AACACCATCCATAAAGTCGTTC	18,64,82,379/ 18,64,82,158,221	CviQI
PMC_30	1H	8951914	G	A	(F) GAACGACTTTATGGATGGTGTT (R) TGCAGTGTCGTTTCTTCTCTTA	220,452/ 672	BsaAI
PMC_31	1H	9312497	G	C	(F) AGATCTCGTTTCGTCTTGGTT (R) ACTGCTATGAACTTGGACTGCT	217,452/ 669	NaeI
PMC_36	1H	9330782	G	C	(F) CTCACGAGTGGCTATGACATTA (R) CTTCAAGTAGGACCTCACGTTC	61,173,106/ 60,61,106,113	MboI
PMC_44	1H	9622061	G	C	(F) TTATGAACTGAATGCTTGTTCG (R) TGAAGATGATACATGGAGGACA	564/ 203,361	StuI
PMC_55	1H	10536902	A	G	(F) GGTCGGGATAGTCATTGAGATA (R) GGTGAAGATGTCTCTCTACCCA	410/ 139,271	BglII
PMC_71	1H	11547580	G	C	(F) TTGTGCATCATTAAGCAGAAAC (R) ATAACTTCTATCAGGGCTGCAA	112,348/ 460	HinP1I
PMC_72	1H	11802894	A	G	(F) GTGATTTGTCCCTTGTCTGTTT (R) AAGAGAGAGCTAGCAGCAAAGA	28,37,40,48,50,291/ 37,40,50,76,291	HpyCH4V
PMC_75	1H	11864697	C	T	(F) GCTTCTTCGACATGGAGTACC (R) GTCTGCTTTGACAGGTTGCT	664/ 367,297	ScaI
PMC_76	1H	11864741	C	G	(F) GCTTCTTCGACATGGAGTACC (R) GTCTGCTTTGACAGGTTGCT	14,36,291,323/ 14,291,359	NlaIII
PMC_80	1H	12613010	A	G	(F) TATGGTCTACTTGGAGCCACTT (R) AGATGCCGAACAATGCTACTAT	496/ 241,255	BstNI
PMC_87	1H	13380333	C	T	(F) TGCTGAGAGGATAAGGAGAGAG (R) TGTAGGTGAACTCATCAACGAC	635/ 218,417	BalI
PMC_95	1H	14101542	C	T	(F) AGGTGGCTTGAAAGATGAAATA (R) AACTTCTGTCGGAACAGCTTTA	117,390/ 507	MboI
PMC_106	1H	14667209	G	C	(F) GTGGGTATCACTCAGGAATTGT (R) AGGGAAGGAATGCTAGGATAAG	106,120,125,205/ 106,120,330	CviQI
PMC_114	1H	15081836	G	A	(F) GGAACAGGCTACAAACCTAGTG (R) TGTTTGAAGTATTCACTCGACG	35,54,250,274/ 35,54,524	Hpy188I
PMC_121	1H	15224929	A	G	(F) CGCGTAATAGTTGATCAAGACA (R) CTGCTTTATTTCCTTACGATGG	50,73,168,399/ 73,218,399	MwoI
PMC_129	1H	19594745	C	T	(F) ACCACTACAAGCTACAGCCCTA (R) TTCGGATAGATACTCAAGCGAT	29,39,105,144,254/ 29,39,105,398	HpyCH4V
PMC_130	1H	19595703	G	A	(F) GGAGTCTCCTCTCCAGAACTTT (R) TATCAGTACCAAGGACCACACA	473/ 103,370	AgeI

The S in amplicon size represents susceptibility, whereas the R indicates the band size for resistance.

### 3.6 Identification of candidate genes through *in-silico* analysis

To identify candidate genes associated with PM resistance in malting barley, 21 non-synonymous SNPs causing amino acid sequence changes were analyzed, leading to the identification of 18 putative candidate genes within the QTL region on chromosome 1 (7.8–19.6 Mb). These candidate genes included PMC_4: *Polyadenylate-binding protein 2* (A/G, 1 SNP), PMC_23: *LINE-1 reverse transcriptase-like* (C/T, 1 SNP), PMC_28: *Cysteine proteinase* (A/G, 1 SNP), PMC_29: *Elongation factor* (G/A, 1 SNP), PMC_30: *Elongation factor* (G/A, 1 SNP), PMC_31: *Pumilio-like protein* (G/C, 1 SNP), PMC_36: *ERD (Early-responsive to dehydration stress) family protein* (G/C, 1 SNP), PMC_44: *Kinetochore protein* sp*c25* (G/C, 1 SNP), PMC_55: *Leucine-rich repeat protein kinase family protein putative* (A/G, 1 SNP), PMC_71: *Pectin acetylesterase* (G/C, 1 SNP), PMC_72: *Sensory neuron membrane protein 1* (A/G, 1 SNP), PMC_75: *Clathrin assembly protein putative expressed* (C/T, 1 SNP), PMC_76: *Clathrin assembly protein putative expressed* (C/G, 1 SNP), PMC_80: *Homeobox protein putative* (A/G, 1 SNP), PMC_87: *Disease resistance protein* (C/T, 1 SNP), PMC_95: *Inorganic pyrophosphatase family protein* (C/T, 1 SNP), PMC_106: *Protoheme IX farnesyltransferase* (G/C, 1 SNP), PMC_114: *Type I inositol-1,4,5-trisphosphate 5-phosphatase 1* (G/A, 1 SNP), PMC_121: *rRNA N-glycosidase* (A/G, 1 SNP), PMC_129: *Methyl-coenzyme M reductase II subunit gamma putative (DUF3741)* (C/T, 1 SNP), and PMC_130: *Methyl-coenzyme M reductase II subunit gamma putative (DUF3741)* (G/A, 1 SNP) (**Supplementary Table 8**).

### 3.7 Validation of CAPS markers based on 21 non-synonymous SNPs

PCR amplification and restriction enzyme digestion were performed for each CAPS marker using the four DNA samples employed in QTL-seq analysis: ‘Hopum’ (susceptible parent, SP), ‘Jeonju 182’ (resistant parent, RP), HvPM_S20 (S20), and HvPM_R20 (R20). Among the 21 non-synonymous SNP-based CAPS markers, band differences between susceptible and resistant phenotypes were observed in 19 markers, excluding PMC_87 and PMC_95. Restriction enzyme digestion of the 19 CAPS markers confirmed that all markers were successfully cleaved (**Supplementary Figure 5**). Using these 19 CAPS markers, an additional validation was conducted with 20 individuals from the susceptible and resistant bulked F_2_ populations analyzed in QTL-seq. In some markers, resistant individuals exhibited band patterns similar to those of susceptible individuals, which is likely due to the dominant inheritance of PM resistance, resulting in a heterozygous genotype (Aa). Ultimately, the band patterns of each CAPS marker in 20 susceptible and 20 resistant individuals were compared with those of the parental lines (**Supplementary Figure 6**). Among the tested markers, 12 CAPS markers (PMC_23, PMC_28, PMC_30, PMC_44, PMC_71, PMC_72, PMC_75, PMC_76, PMC_80, PMC_106, PMC_114, and PMC_121) consistently distinguished between susceptible and resistant individuals, with susceptible samples displaying the expected susceptible band pattern and resistant samples exhibiting the corresponding resistant band pattern (**Table 4**).

**Table 4:** Genotyping of F_2_ individuals using 19 cleaved amplified polymorphic sequence markers.

Marker	Susceptible bulk																				Resistance bulk																			
	1	2	3	4	5	6	7	8	9	10	11	12	13	14	15	16	17	18	19	20	1	2	3	4	5	6	7	8	9	10	11	12	13	14	15	16	17	18	19	20
PMC_4	S	S	S	S	S	S	S	S	S	S	S	S	S	S	S	S	S	S	S	R	R	R	R	R	R	R	R	R	R	R	R	R	R	R	R	R	R	R	R	R
PMC_23	S	S	S	S	S	S	S	S	S	S	S	S	S	S	S	S	S	S	S	S	R	R	R	R	R	R	R	R	R	R	R	R	R	R	R	R	R	R	R	R
PMC_28	S	S	S	S	S	S	S	S	S	S	S	S	S	S	S	S	S	S	S	S	R	R	R	R	R	R	R	R	R	R	R	R	R	R	R	R	R	R	R	R
PMC_29	S	R	S	S	S	S	S	S	S	S	S	S	S	S	S	S	S	S	S	S	R	R	R	R	R	R	R	R	R	R	R	R	R	R	R	R	R	R	R	R
PMC_30	S	S	S	S	S	S	S	S	S	S	S	S	S	S	S	S	S	S	S	S	R	R	R	R	R	R	R	R	R	R	R	R	R	R	R	R	R	R	R	R
PMC_31	–	–	S	S	S	S	S	S	–	S	S	S	S	S	S	S	–	S	S	S	R	R	R	R	R	R	R	R	R	R	R	R	R	R	R	R	R	R	R	R
PMC_36	S	S	S	S	S	S	S	S	S	S	S	S	S	S	S	S	R	S	S	S	R	R	R	R	R	R	R	R	R	R	R	R	R	R	R	R	R	R	R	R
PMC_44	S	S	S	S	S	S	S	S	S	S	S	S	S	S	S	S	S	S	S	S	R	R	R	R	R	R	R	R	R	R	R	R	R	R	R	R	R	R	R	R
PMC_55	S	S	S	S	S	S	S	S	S	S	S	S	S	S	S	S	S	S	S	S	R	S	S	S	S	S	S	R	S	S	S	S	S	S	S	S	S	S	R	S
PMC_71	S	S	S	S	S	S	S	S	S	S	S	S	S	S	S	S	S	S	S	S	R	R	R	R	R	R	R	R	R	R	R	R	R	R	R	R	R	R	R	R
PMC_72	S	S	S	S	S	S	S	S	S	S	S	S	S	S	S	S	S	S	S	S	R	R	R	R	R	R	R	R	R	R	R	R	R	R	R	R	R	R	R	R
PMC_75	S	S	S	S	S	S	S	S	S	S	S	S	S	S	S	S	S	S	S	S	R	R	R	R	R	R	R	R	R	R	R	R	R	R	R	R	R	R	R	R
PMC_76	S	S	S	S	S	S	S	S	S	S	S	S	S	S	S	S	S	S	S	S	R	R	R	R	R	R	R	R	R	R	R	R	R	R	R	R	R	R	R	R
PMC_80	S	S	S	S	S	S	S	S	S	S	S	S	S	S	S	S	S	S	S	S	R	R	R	R	R	R	R	R	R	R	R	R	R	R	R	R	R	R	R	R
PMC_106	S	S	S	S	S	S	S	S	S	S	S	S	S	S	S	S	S	S	S	S	R	R	R	R	R	R	R	R	R	R	R	R	R	R	R	R	R	R	R	R
PMC_114	S	S	S	S	S	S	S	S	S	S	S	S	S	S	S	S	S	S	S	S	R	R	R	R	R	R	R	R	R	R	R	R	R	R	R	R	R	R	R	R
PMC_121	S	S	S	S	S	S	S	S	S	S	S	S	S	S	S	S	S	S	S	S	R	R	R	R	R	R	R	R	R	R	R	R	R	R	R	R	R	R	R	R
PMC_129	S	S	S	S	S	S	S	S	S	S	S	S	R	S	S	S	S	S	S	S	R	R	R	R	R	R	R	R	R	R	R	R	R	R	R	R	R	S	R	R
PMC_130	S	S	S	S	S	S	S	S	S	S	S	S	R	S	S	S	S	S	S	S	R	R	R	R	R	R	R	R	R	R	R	R	R	R	R	R	R	S	R	R

### 3.8 Selection of candidate markers using malting barley cultivars

**Figure 5:**
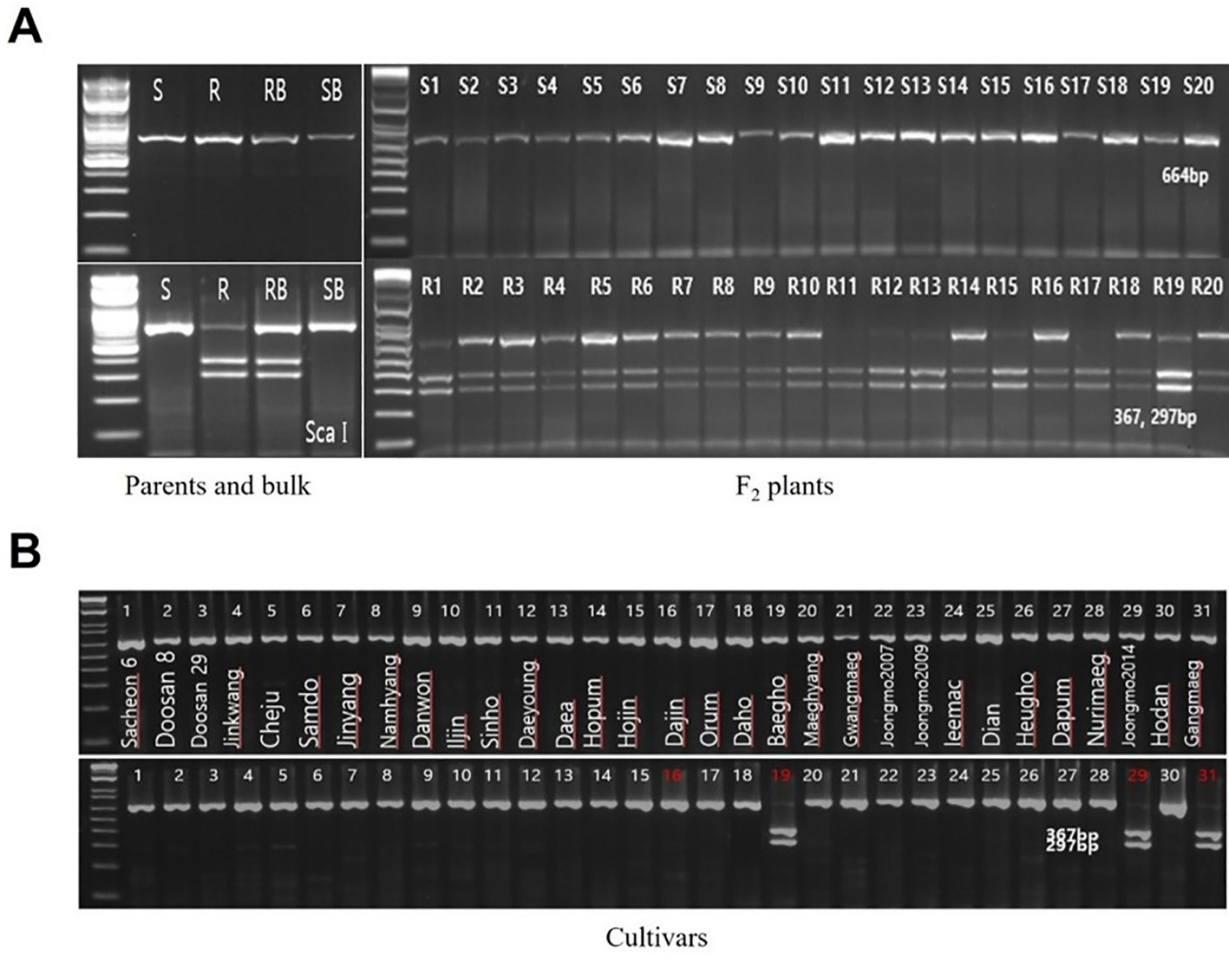
**(A)** Genotyping of parental lines and F_2_ individuals using the PMC_75 marker. The PCR product size was 664 bp, and after restriction enzyme (Sca I) digestion, the resistant individuals exhibited cleavage into 367 and 297 bp fragments. **(B)** Identification of Korean malting barley cultivars using the PMC_75 marker. The powdery mildew-resistant varieties include ‘Dajin’ (16), ‘Baegho’ (19), ‘Jungmo2014’ (29), and ‘Gangmaeg’ (31), whereas all other varieties are susceptible. ‘Baegho,’ ‘Jungmo2014,’ and ‘Gangmaeg’ are resistant varieties derived from ‘Nishinochikara’.

The 12 CAPS markers validated in the F_2_ population were applied to 31 malting barley cultivars developed in Korea to assess their applicability (**Supplementary Figure 7**). However, no single marker was found to be perfectly associated with PM resistance across all resistant cultivars (‘Dajin,’ ‘Baegho,’ ‘Jungmo2014,’ and ‘Gangmaeg’). Nevertheless, the PMC_75 marker consistently showed resistance-associated bands in ‘Baegho,’ ‘Jungmo2014,’ and ‘Gangmaeg’ but not in ‘Dajin’ (**Table 5**, **Figure 5**). This observation is likely due to the fact that ‘Baegho,’ ‘Jungmo2014,’ and ‘Gangmaeg’ inherited their resistance genes from ‘Nishinochikara,’ a Japanese PM-resistant cultivar, whereas ‘Dajin’ did not. The resistant cultivar used in this genetic analysis, ‘Jeonju 182,’ also originated from a ‘Nishinochikara’-derived resistant lineage (Hyun et al., 2006b; Kim et al., 2011; Tsuru et al., 1990). ‘Jeonju 182’ is derived from a ‘Baegho’/’Sukai Golden’ (‘Kanto Nijo 25’/’Tochikei 216’) cross, and ‘Sukai Golden’ is a highly PM-resistant cultivar (Taniguchi et al., 2001). The PM resistance of ‘Sukai Golden’ is inherited from ‘Kanto Nijo 25’ (Furusho et al., 1990). Investigation of the breeding pedigree of ‘Sukai Golden’ and ‘Nishinochikara’ reveals that the crossing parents of ‘Nishinochikara’ share the same parental lineage with ‘Sukai Golden,’ suggesting that it possesses strong PM resistance (Iida et al., 1992; Taketa et al., 2023). Additionally, ‘Baegho’ is derived from ‘Azuma Golden’/’Nishinochikara,’ ‘Jungmo2014’ from ‘Iksan 139’ (‘Kanto Nijo 7’/’Nishinochikara’)/’Stirling,’ and ‘Gangmaeg’ from ‘Daho’/’Sukai Golden,’ all of which include PM-resistant genetic resources in their parental backgrounds. Conversely, ‘Dajin’ is derived from ‘Misato Golden’/’Suwon 295’//’Suwon 295,’ with its PM resistance gene originating from ‘Suwon 295’ rather than ‘Nishinochikara’ (**Supplementary Figure 8**), which explains why it did not show resistance in this marker analysis. For PMC_75 marker validation, the PCR product size was 664 bp, and after restriction enzyme digestion, resistant samples showed two distinct bands (367 and 297 bp), confirming complete co-segregation with PM-resistant and susceptible phenotypes in the F_2_ population (**Figure 5**). These results strongly suggest that the candidate gene, which is associated with PMC_75, *HORVU.MOREX.r3.1HG0005790* (Clathrin Assembly Protein Putative Expressed), is closely related to PM resistance derived from ‘Jeonju 182.’

**Table 5:** Genotyping of malting barley cultivars using 12 cleaved amplified polymorphic sequence markers.

Marker Cultivar	PMC_23	PMC_28	PMC_30	PMC_44	PMC_71	PMC_72	PMC_75	PMC_76	PMC_80	PMC_106	PMC_114	PMC_121
1. Sacheon6	S	S	S	S	S	S	S	S	S	S	S	S
2. Doosan8	R	R	S	S	R	R	S	R	S	S	–	R
3. Doosan29	S	S	S	S	S	S	S	S	S	S	S	S
4. Jinkwang	S	S	S	S	S	S	S	S	S	S	S	R
5. Jeju	S	S	S	S	S	S	S	S	S	S	S	R
6. Samdo	S	S	S	S	S	S	S	S	S	S	S	R
7. Jinyang	R	R	S	S	R	S	S	R	R	R	R	R
8. Namhyang	R	R	S	S	R	S	S	R	R	R	R	R
9. Danwon	R	R	S	S	R	S	S	R	R	R	R	R
10. Iljin	R	R	S	S	R	S	S	R	R	R	R	R
11. Shinho	R	R	S	R	R	S	S	R	R	R	R	R
12. Daeyoung	S	S	S	S	S	S	S	S	S	S	S	R
13. Daea	R	R	S	S	R	S	S	R	R	R	R	R
14. Hopum	S	S	S	S	S	S	S	S	S	S	S	S
15. Hojin	R	R	S	R	R	S	S	R	R	R	R	R
16. DaJin	R	R	S	R	R	S	S	R	R	S	S	R
17. Oruem	S	R	S	S	R	R	S	R	S	S	R	R
18. Daho	R	S	S	S	S	S	S	S	S	S	S	S
19. Baegho	S	R	R	R	R	R	R	R	R	R	R	R
20. Machyang	R	R	S	S	S	R	S	R	R	S	S	S
21. Kwangmaeg	R	S	S	S	S	S	S	S	S	S	S	S
22. Joogmo2007	R	S	S	S	S	S	S	S	S	S	S	S
23. Joongmo2009	R	R	S	S	R	S	S	R	R	R	R	R
24. Leemac	R	R	S	S	R	S	S	R	R	R	R	R
25. Dian	R	S	S	S	S	S	S	S	S	S	S	S
26. Heugho	S	R	S	S	R	R	S	R	S	S	R	R
27. Dapum	R	S	S	S	S	S	S	S	S	S	S	S
28. Nurimaeg	R	R	S	S	R	S	S	R	R	R	R	R
29. Joongmo2014	S	R	S	R	R	R	R	R	R	R	R	R
30. Hodan	R	R	R	S	R	S	S	R	R	R	R	R
31. Gangmaeg	R	R	S	R	R	R	R	R	R	R	R	R

### 3.9 Expression analysis of candidate genes

**Figure 6:**
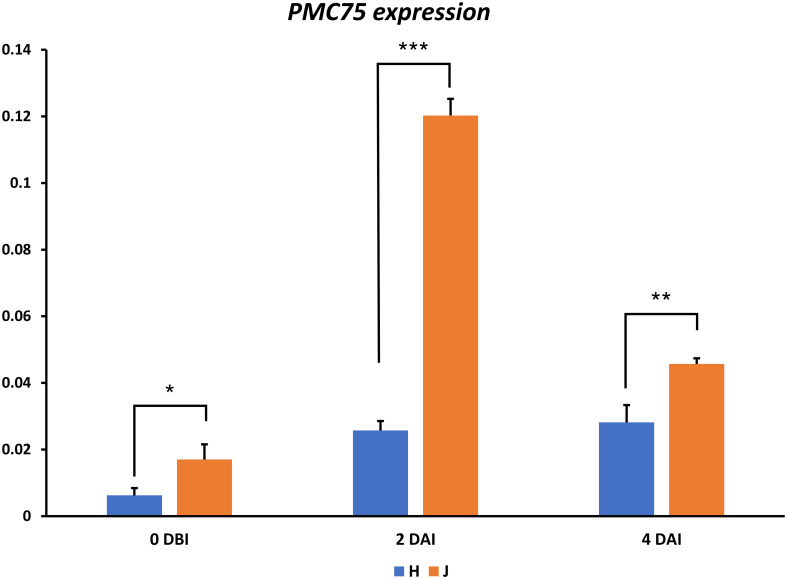
Expression analysis of the *PMC_75* candidate gene using RT-qPCR. Relative expression levels of the *PMC_75* gene compared with those of the housekeeping gene (*β-TUB6*). Error bars represent ± standard deviation (SD), and statistically significant differences were determined using Student’s *t*-test and indicated using asterisks (^*^ *p* < 0.05, ^**^ *p* < 0.01, ^***^ *p* < 0.001).

Quantitative real-time PCR (qPCR) was performed to validate the expression pattern of the candidate gene *1HG0005790*, associated with the PMC_75 marker, which was presumed to be linked to PM resistance in malting barley. The experiment was conducted using seedling leaves of the susceptible (‘Hopum,’ H) and resistant (‘Jeonju 182,’ J) varieties at three time points: pre-inoculation (0 days after inoculation, 0 DAI), at the initial stage of pathogen invasion and conidia formation (2 DAI), and at the stage of increased conidia production (4 DAI). Gene expression analysis revealed a significant difference in *1HG0005790* expression between the two varieties even before inoculation, with expression levels at 0 DAI recorded as 0.00624 in ‘Hopum’ and 0.01701 in ‘Jeonju 182.’ At 2 DAI, expression levels increased substantially, reaching 0.02572 in ‘Hopum’ and 0.12026 in ‘Jeonju 182,’ showing a marked contrast between the two varieties (**Figure 6**). By 4 DAI, expression levels slightly decreased to 0.02814 in ‘Hopum’ and 0.04567 in ‘Jeonju 182,’ which is likely due to a shift in defense responses following pathogen invasion and completion of conidia formation. Since significant differences in gene expression were observed between the susceptible and resistant varieties across all time points, this expression pattern strongly suggests that *1HG0005790* is a potential candidate gene associated with PM resistance.

## 4 Discussion

SNPs are the most abundant form of genetic variation across the genome and are stably inherited across generations. SNP markers are highly reliable due to their low mutation rates, high reproducibility, and minimal detection errors, enabling precise genotypic differentiation (Jehan and Lakhanpaul, 2006). Among various types of SNPs, non-synonymous SNPs alter amino acid sequences in proteins, potentially affecting protein–protein interactions and influencing disease resistance (Yates and Sternberg, 2013). In rice, non-synonymous SNPs are reportedly highly associated with functional variations in disease resistance-related genes, such as WRKY transcription factors (Srivastava et al., 2014). Similarly, in cucumber, studies on PM resistance have demonstrated differential gene expression patterns between resistant and susceptible lines in genes harboring non-synonymous SNPs (Xu et al., 2016). In the current study, marker validation focused on non-synonymous SNPs, which play a critical role in amino acid sequence alterations (**Table 3**, **Supplementary Figures 5**-**7**). However, additional consideration should be given not only to variations located in introns and UTRs, as these elements regulate gene expression at the transcriptional and post-transcriptional levels, but also to coding-region InDels can lead to frameshift mutations that may significantly impact gene function (Barrett et al., 2012; de la Chaux et al., 2007).

**Figure 7:**
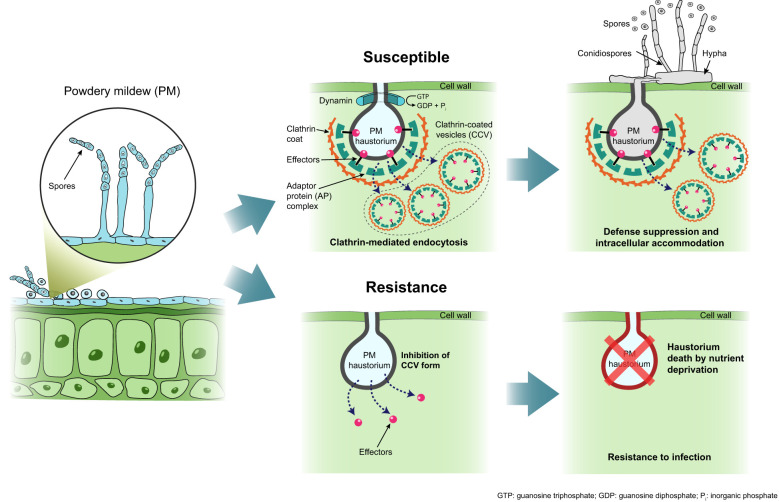
Powdery mildew development mechanisms in barley. In susceptible cultivars, which express clathrin assembly protein, vesicle formation is actively induced following haustorium development by the pahtogen. Clathrin-coated vesicle (CCV) is formed by the assembly of pathogen effectors, adapter protein (AP) complex, and clathrin coat. In resistant cultivars, absence of clathrin assembly protein expression disrupts vesicle formation, preventing disease progression and sproe developmnet.

The candidate gene linked to the PMC_75 marker is annotated as Clathrin Assembly Protein Putative Expressed, suggesting a potential role in clathrin-mediated endocytosis (CME)(**Supplementary Table 8**). Clathrin is an essential protein for vesicle trafficking, playing a critical role in intracellular transport and pathogen–host interactions (Royle, 2006). The C/T SNP identified in the PMC_75 marker may disrupt the normal function of Clathrin Assembly Protein, potentially influencing PM resistance. Previous studies have reported that mutations in Clathrin Heavy Chain 2 (CHC2) reduce endocytosis efficiency, leading to increased resistance against certain pathogens (Wu et al., 2015). This suggests that CME may be involved in the uptake of pathogen-secreted proteins or signaling components into host cells. PM pathogens establish infection by forming haustoria, which facilitate the delivery of effector proteins into host cells. Recent research has proposed that these effectors may enter plant cells through CME (Wang et al., 2023). If the SNP variation in the PMC_75 marker disrupts the normal function of Clathrin Assembly Protein Putative Expressed, the pathogen may experience difficulties in successfully invading host cells. These findings suggest that CME may play a critical role in the PM infection process and indicate that the PMC_75 marker may contribute to resistance expression (**Figure 7**). Furthermore, the present study represents one of the first validations of this association in relation to barley PM resistance.

RT-qPCR analysis revealed that at day 0, the candidate gene exhibited higher expression in the resistant cultivar compared with that in the susceptible cultivar, even in the absence of the pathogen(**Figure 6**). This suggests that the resistant cultivar has a relatively higher basal expression level of the gene, which may contribute to its defense mechanisms. A similar pattern has been reported in studies on Verticillium wilt resistance in olive (*Olea europaea*), where resistant cultivars exhibited higher basal expression of defense-related genes in roots compared with that in susceptible cultivars, even before pathogen exposure. Likewise, in rice root-knot nematode resistance, certain defense-related genes showed higher expression in resistant cultivars than in susceptible ones, regardless of infection status, aligning with our findings (Ramírez-Tejero et al., 2021; Kumari et al., 2016). At day 2 post-inoculation, the resistant cultivar exhibited a peak in candidate gene expression (**Figure 6**), likely due to a non-synonymous SNP variation causing an amino acid substitution in the Clathrin Assembly Protein, potentially leading to a functional mutation. This may have disrupted the pathogen’s ability to invade plant cells, as haustorium formation and other key infection processes typically occur within the first 0–2 days of infection. By day 4, the expression of the candidate gene in the resistant cultivar had declined (**Figure 6**). This pattern is consistent with time-course profiling studies in Arabidopsis, which analyzed the expression of defense-related genes at 0, 0.5, 2, and 4 days post-inoculation, showing a similar transition. This decrease does not indicate a weakening of resistance but rather suggests a dynamic regulation of plant defense mechanisms, where the initial immune response shifts to a subsequent defense phase (Wang et al., 2011).

QTL-seq is a rapid and cost-effective approach for QTL identification, and in the present study, it effectively facilitated the identification of candidate QTLs associated with PM resistance in barley (**Figure 4**, **Supplementary Tables 5**, **6**). However, several limitations exist. First, as QTL-seq is based on the BSA approach, its accuracy can be reduced when trait-associated variants are identified using a small number of individuals due to an incomplete correlation between genotype and phenotype. In the present study, DNA pools were constructed using 20 resistant and 20 susceptible individuals, but incorporating a larger sample size could further enhance the reliability of the identified QTLs (Shen and Messer, 2022). Second, without functional validation of variants within the candidate QTL regions, it remains uncertain whether these variants directly influence trait expression. While multiple variants were identified through QTL-seq in this study, their direct role in regulating resistance traits has not been verified. To address this limitation, additional functional validation using CRISPR/Cas9-based gene editing is required (Tsakirpaloglou et al., 2023). Furthermore, RNA-seq-based transcriptome analysis should be conducted to precisely compare gene expression differences between resistant and susceptible varieties, enabling the identification of more reliable candidate genes. Goyer et al. (2015) performed RNA-seq analysis to compare gene expression profiles between susceptible and resistant potato (*Solanum tuberosum*) varieties in response to potato virus Y (PVY), identifying differentially expressed genes during early infection and proposing their functional roles in resistance mechanisms. A similar approach could provide a more systematic evaluation of genes associated with PM resistance. Third, the influence of environmental factors cannot be excluded. Although PM resistance was evaluated under controlled growth conditions in the present study, various environmental factors such as temperature, humidity, and soil conditions could affect resistance expression under field conditions (El-Soda et al., 2014). Therefore, future studies should conduct QTL validation across multiple environments to assess reproducibility and incorporate genotype-by-environment (G×E) interactions into the analysis.

In the present study, two independent QTL-seq analyses were performed using each of the parental lines, leading to the identification of approximately 2,130 common variants associated with PM resistance. Based on these findings, a set of candidate resistance genes was explored. Unlike conventional QTL-seq studies that rely on a single analysis, the current study applied an independent QTL-seq approach for each parent and selected only the variants commonly detected in both analyses (**Figure 4**, **Supplementary Table 5**, **6**). This strategy enhanced the reliability of the identified variants by reducing potential genotype–phenotype association errors, thereby improving the precision of resistance gene identification. This approach provides a key advantage over traditional single QTL-seq studies, as it minimizes errors caused by genetic variation and enables a more precise identification of resistance-related genes. Future studies should further investigate candidate genes conserved in other plants to gain deeper insights into the mechanisms of pathogen resistance, including the role of Clathrin in disease defense. Additionally, the PMC_75 marker identified in this study has the potential to be utilized in MAS for breeding PM-resistant barley cultivars, contributing to the development of improved resistant varieties.

## Data Availability

The datasets presented in this study can be found in online repositories. The name of the repository/repositories and accession number(s) can be found below: NCBI accession number PRJNA1245611.
