# Supplementary material for: Development of cleaved amplified polymorphic sequence marker for powdery mildew resistance in Korean malting barley using QTL-seq
Source: Front Plant Sci. 2025 May 12;16:1596811. doi: 10.3389/fpls.2025.1596811 (PMC12104202; doi:10.3389/fpls.2025.1596811)
Supplement: Supplementary file 1 [file DataSheet1.zip › Supplementary Figure 5, 6, 7.docx]

| PCR | Restriction enzyme | PCR | Restriction enzyme |
| --- | --- | --- | --- |
| 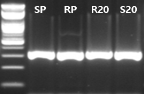 | 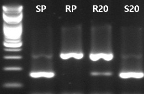 | 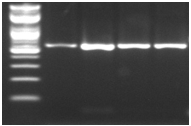 | 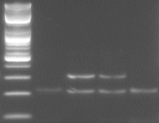 |
| PMC_4, 327bp, 60℃ | BstUⅠ | PMC_23, 520bp, 66℃ | NlaⅢ |
| 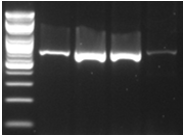 | 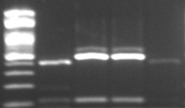 | 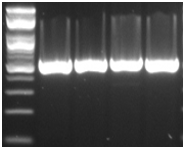 | 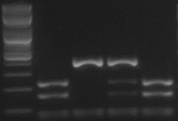 |
| PMC_28, 622bp, 60℃ | HpaⅡ | PMC_29, 543bp, 60℃ | CviQⅠ |
| 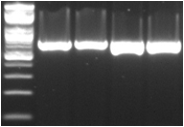 | 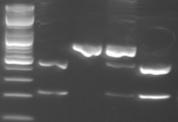 | 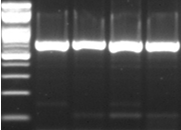 | 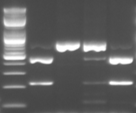 |
| PMC_30, 672bp, 60℃ | BsaAⅠ | PMC_31, 669bp, 60℃ | NaeⅠ |
| 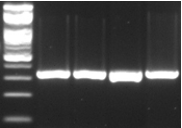 | 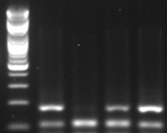 | 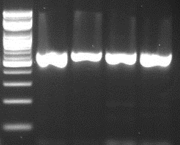 | 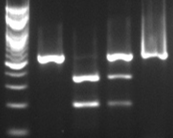 |
| PMC_36, 340bp, 60℃ | MboⅠ | PMC_44, 564bp, 60℃ | StuⅠ |
| 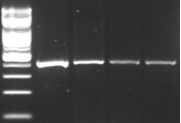 | 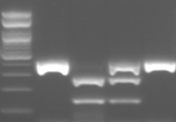 | 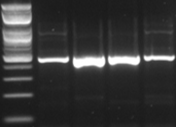 | 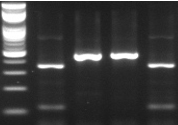 |
| PMC_55, 410bp, 66℃ | BglⅡ | PMC_71, 460bp, 60℃ | HinP1Ⅰ |
| 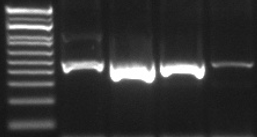 | 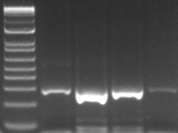 | 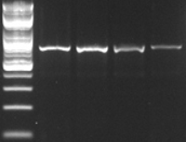 | 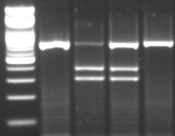 |
| PMC_72, 494bp, 60℃ | HpyCH4Ⅴ | PMC_75, 664bp, 66℃ | ScaⅠ |
| 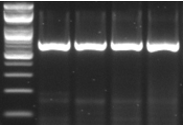 | 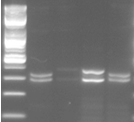 | 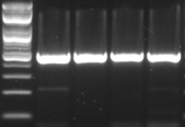 | 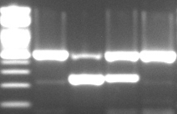 |
| PMC_76, 664bp, 60℃ | NlaⅢ | PMC_80, 496bp, 60℃ | BstNⅠ |
| 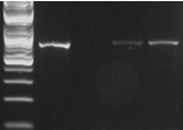 | Fail | 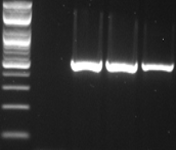 | Fail |
| PMC_87, 635bp, 60℃ |  | PMC_95, 507bp, 60℃ |  |
| 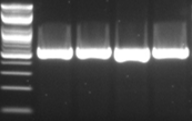 | 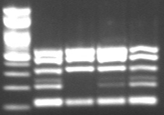 | 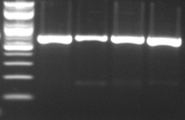 | 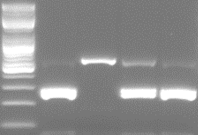 |
| PMC_106, 556bp, 60℃ | BsrⅠ | PMC_114, 613bp, 68℃ | Hpy188Ⅰ |
| 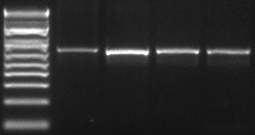 | 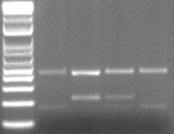 | 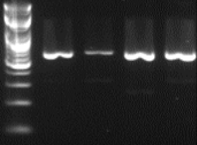 | 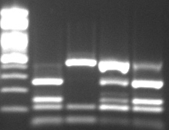 |
| PMC_121, 690bp, 66℃ | MwoⅠ | PMC_129, 571bp, 64℃ | HpyCH4Ⅴ |
| 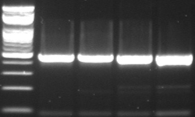 | 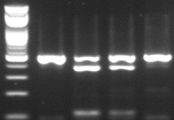 | 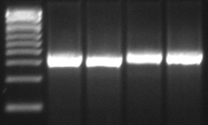 |  |
| PMC_130, 473bp, 60℃ | AgeⅠ | PMI, 373bp, 59℃ |  |

**Supplementary Figure 5** Validation of 21 cleaved amplified polymorphic sequence markers in parental lines and bulk samples. Marker name, product size, PCR annealing time.

| PMC_4  (PCR) | 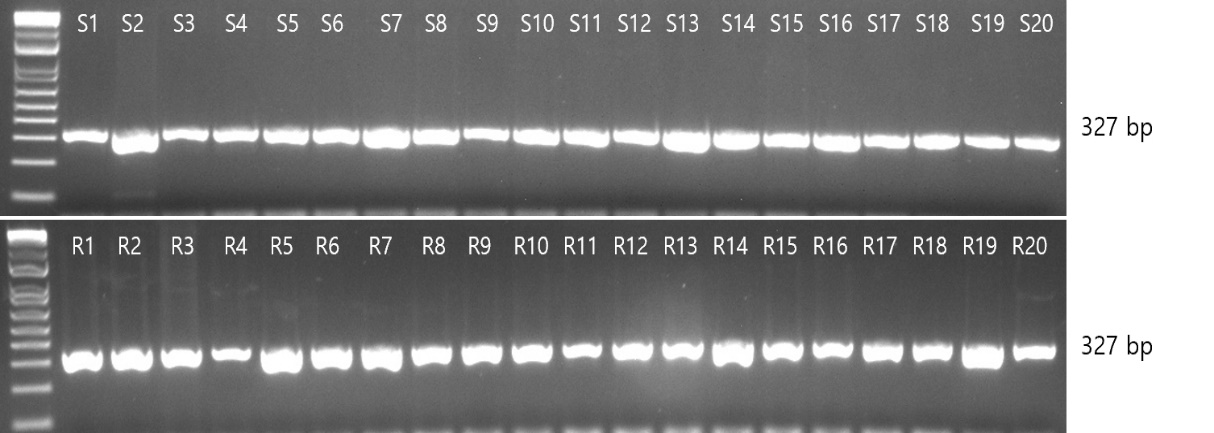 |
| --- | --- |
| PMC_4  (RE) | 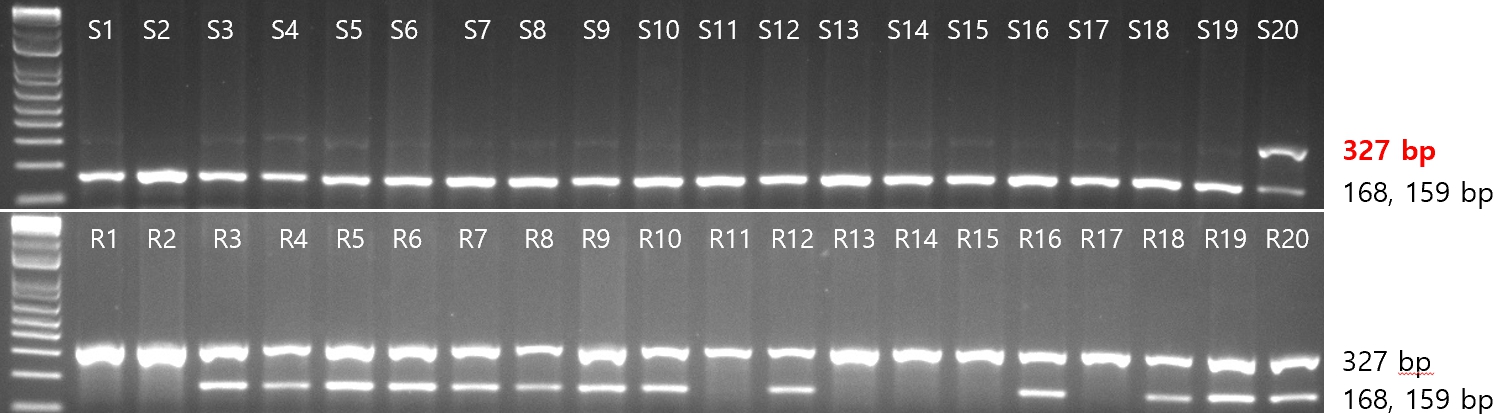 |
| PMC_23  (PCR) | 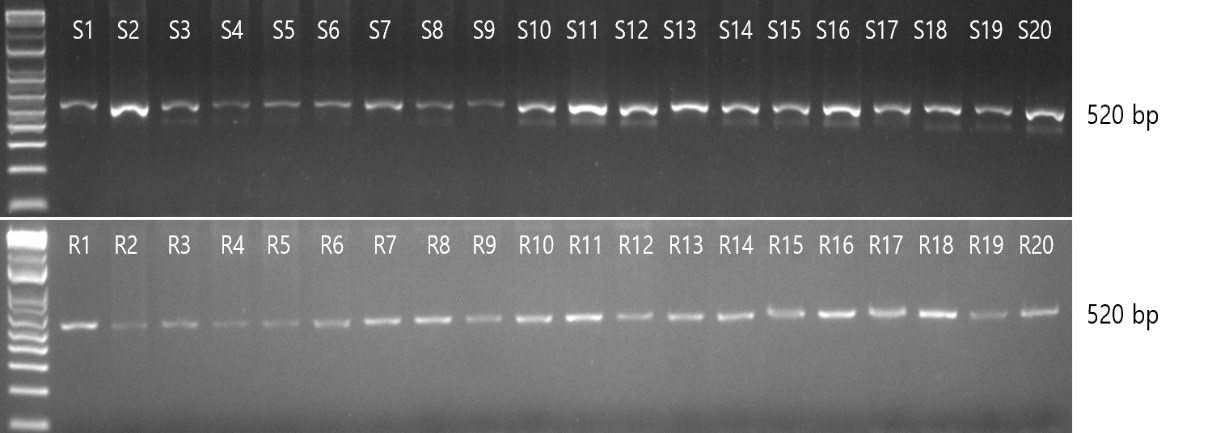 |
| PMC_23  (RE) | 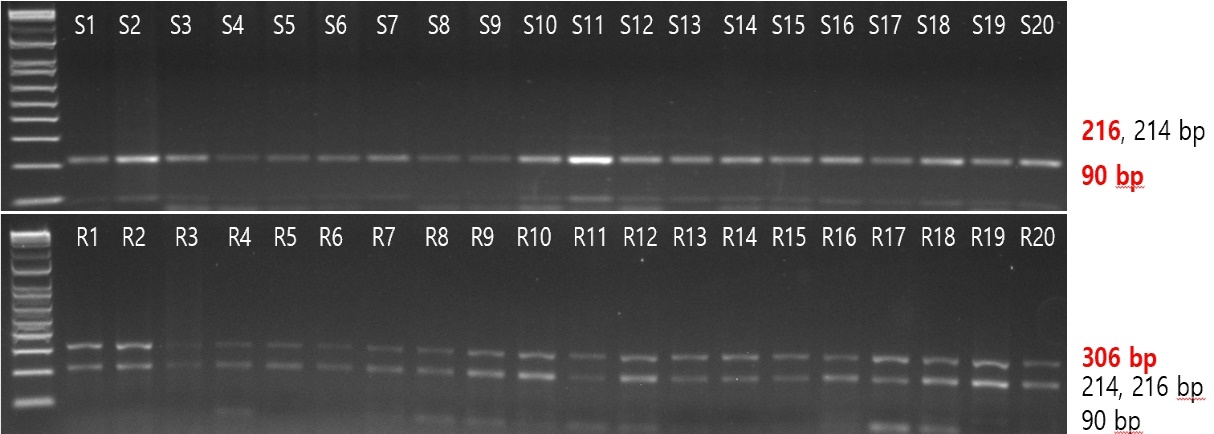 |
| PMC_28  (PCR) | 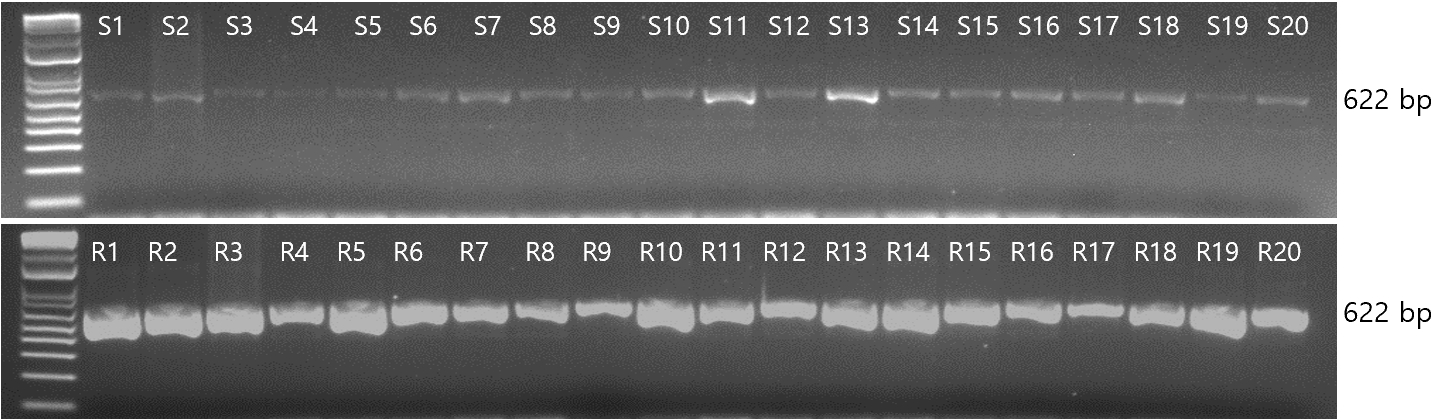 |
| PMC_28  (RE) | 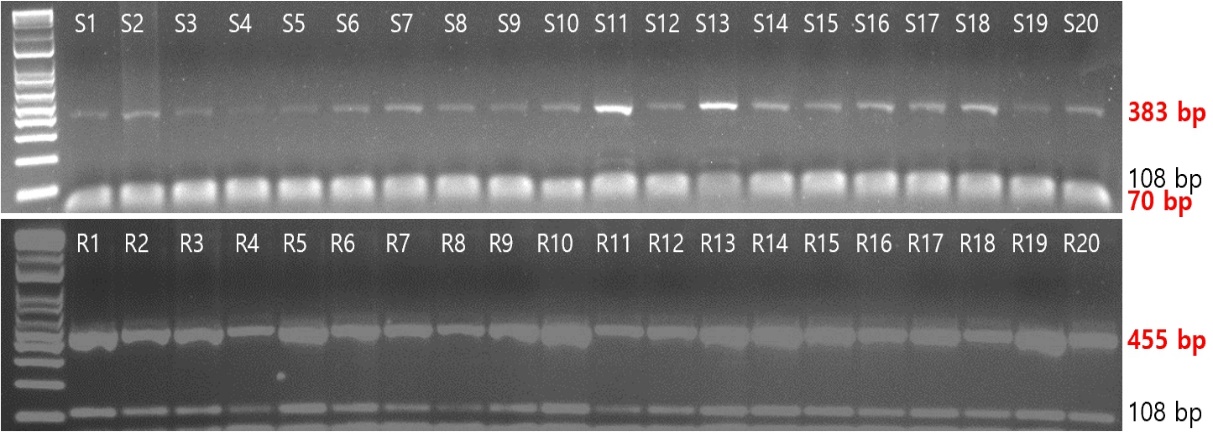 |
| PMC_29  (PCR) | 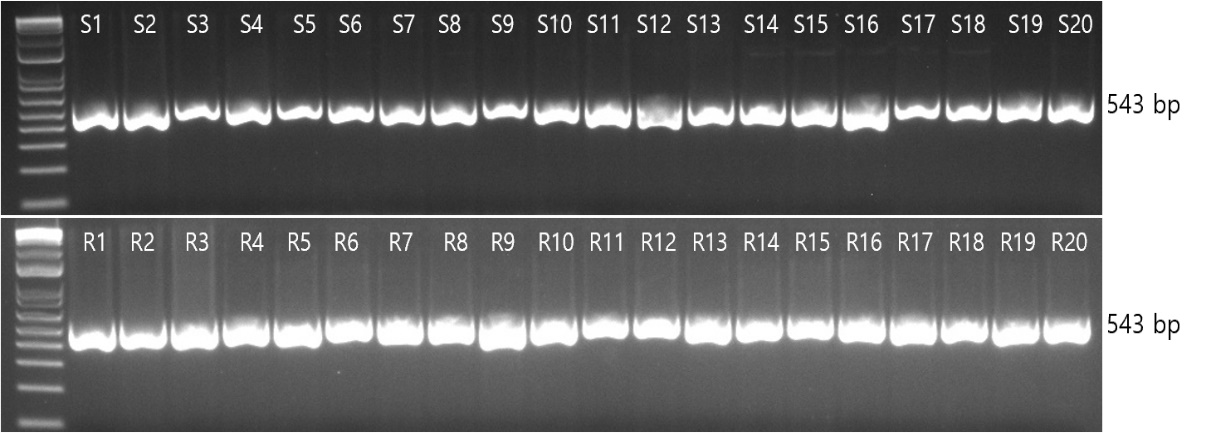 |
| PMC_29  (RE) | 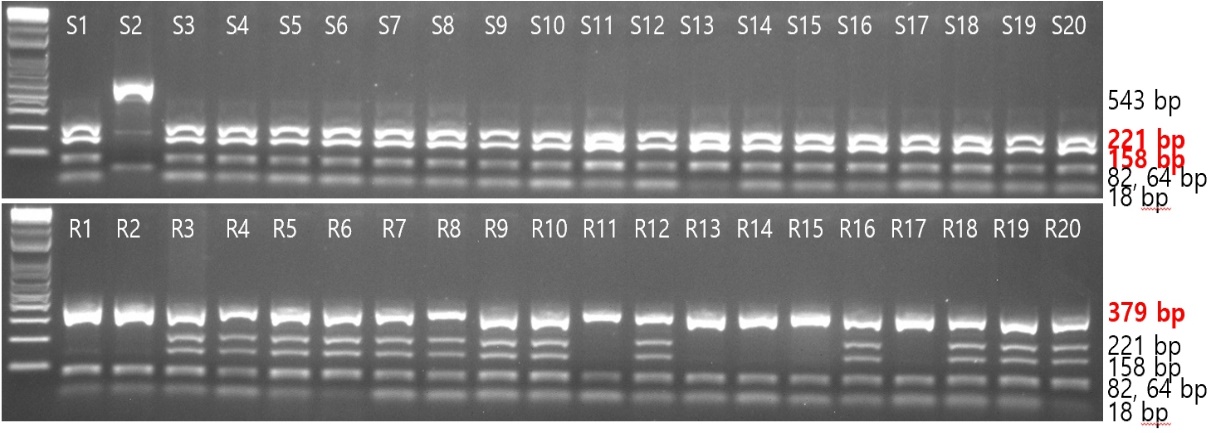 |
| PMC_30  (PCR) | 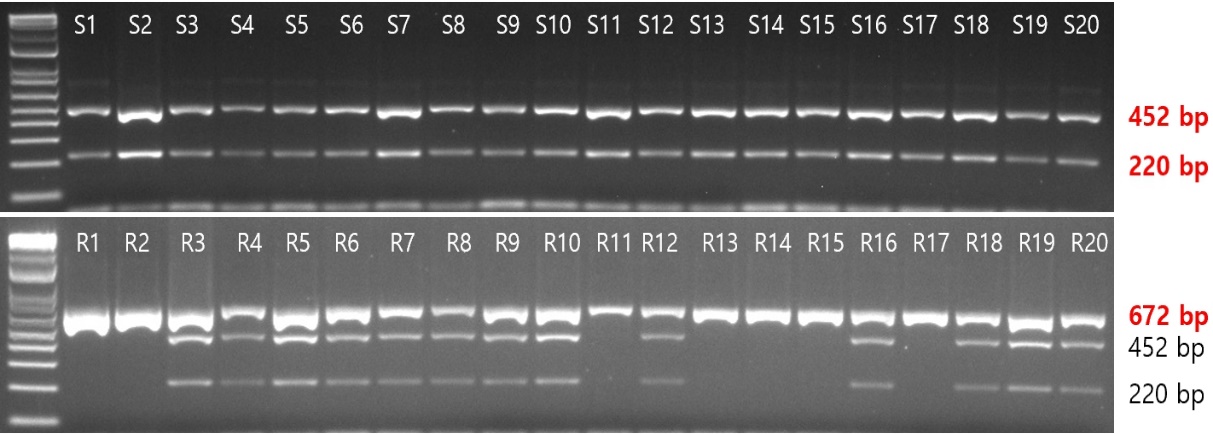 |
| PMC_30  (RE) | 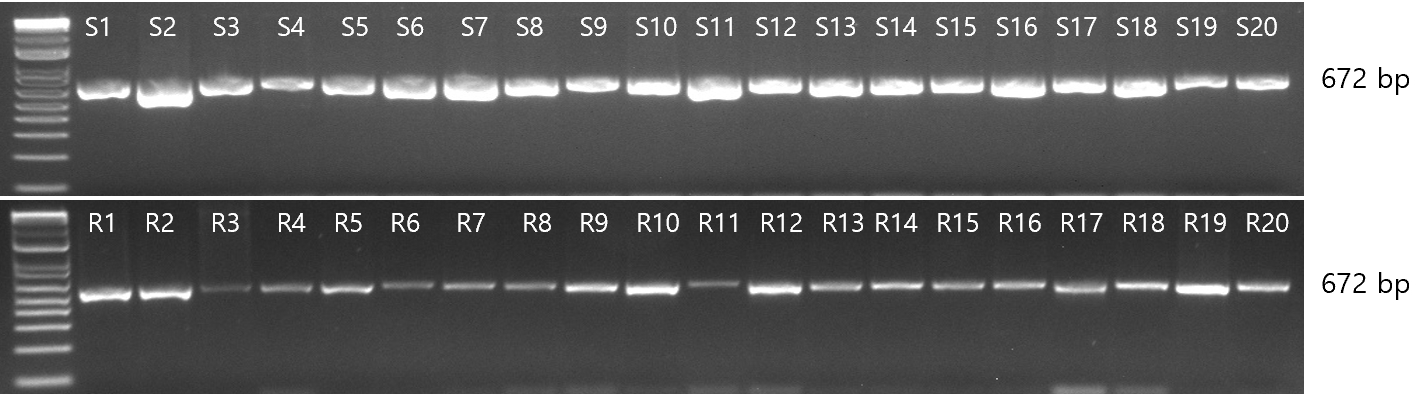 |
| PMC_31  (PCR) | 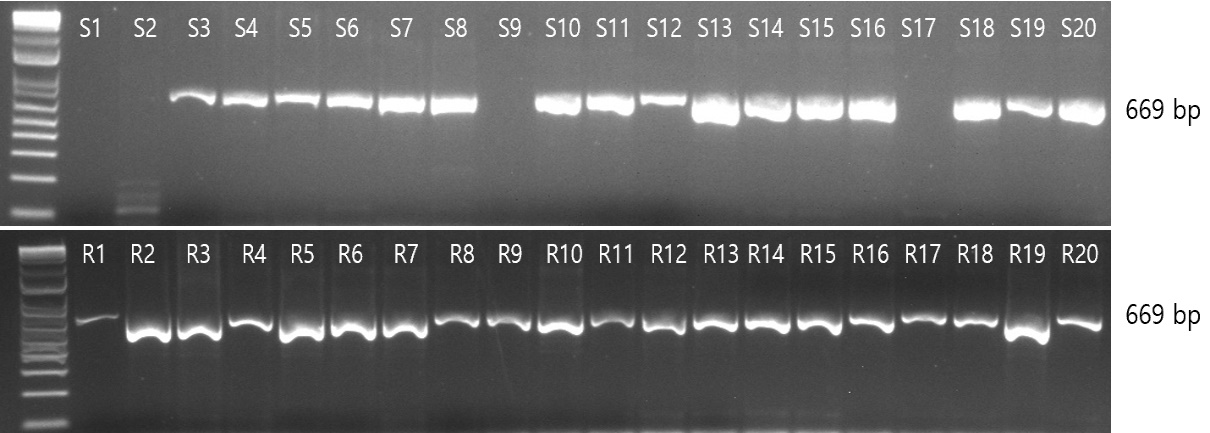 |
| PMC_31  (RE) | 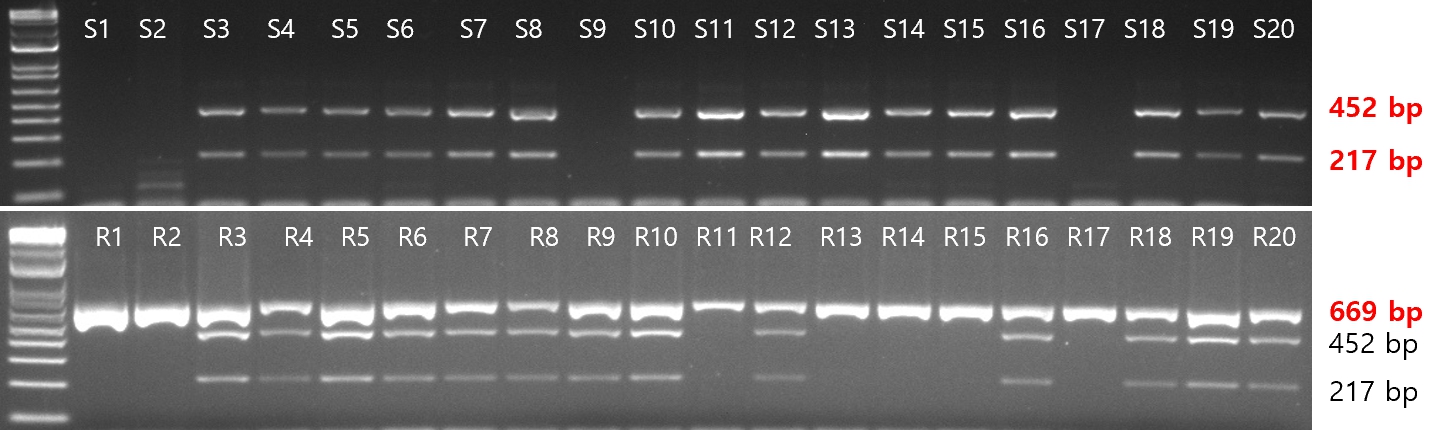 |
| PMC_36  (PCR) | 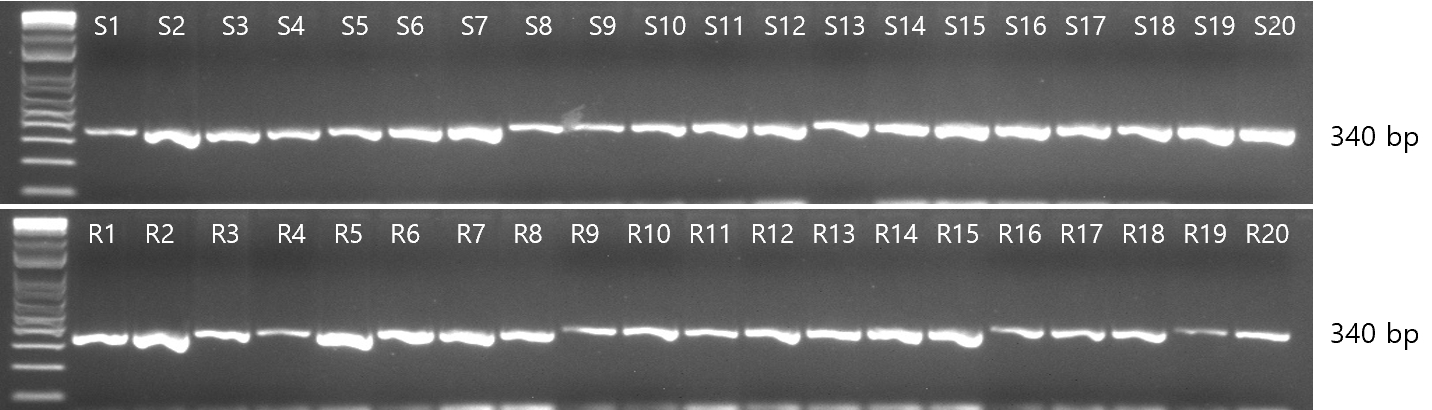 |
| PMC_36  (RE) | 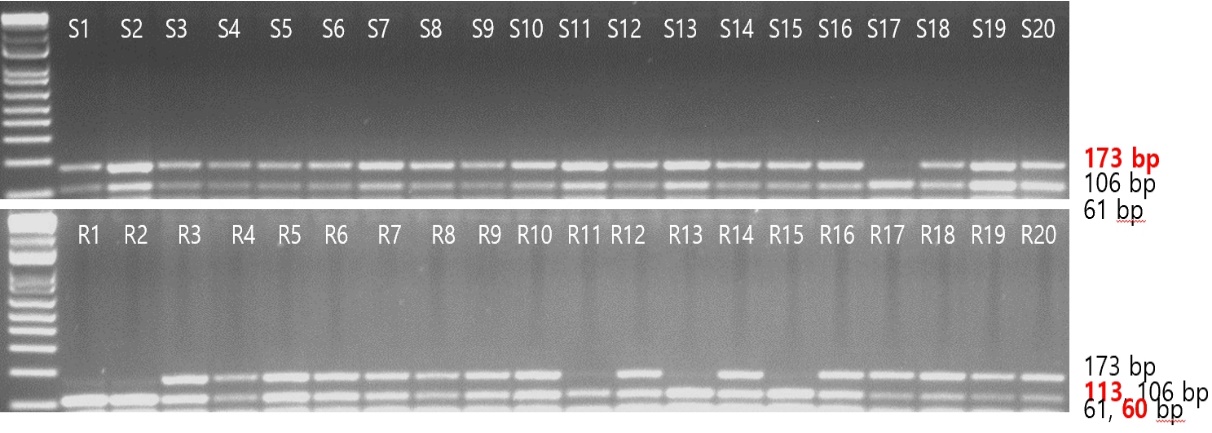 |
| PMC_44  (PCR) | 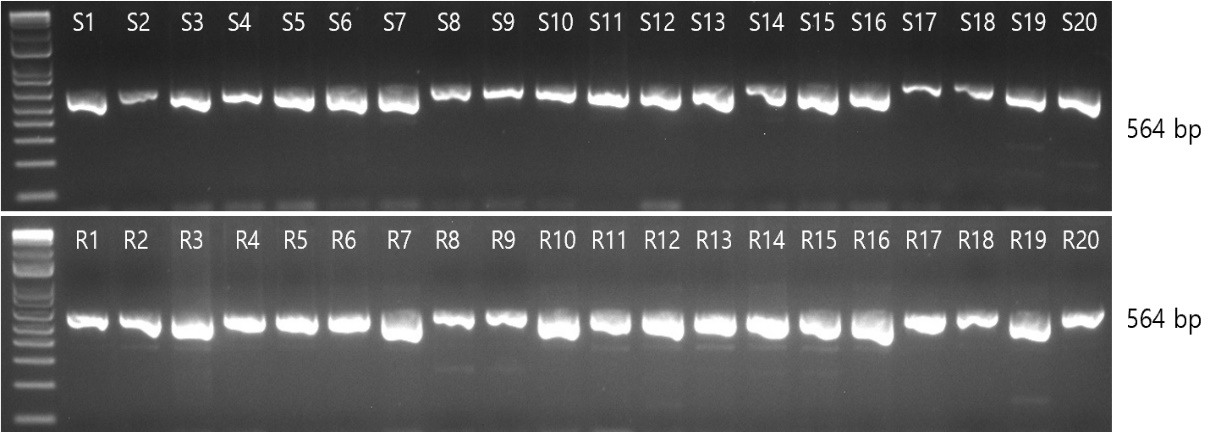 |
| PMC_44  (RE) | 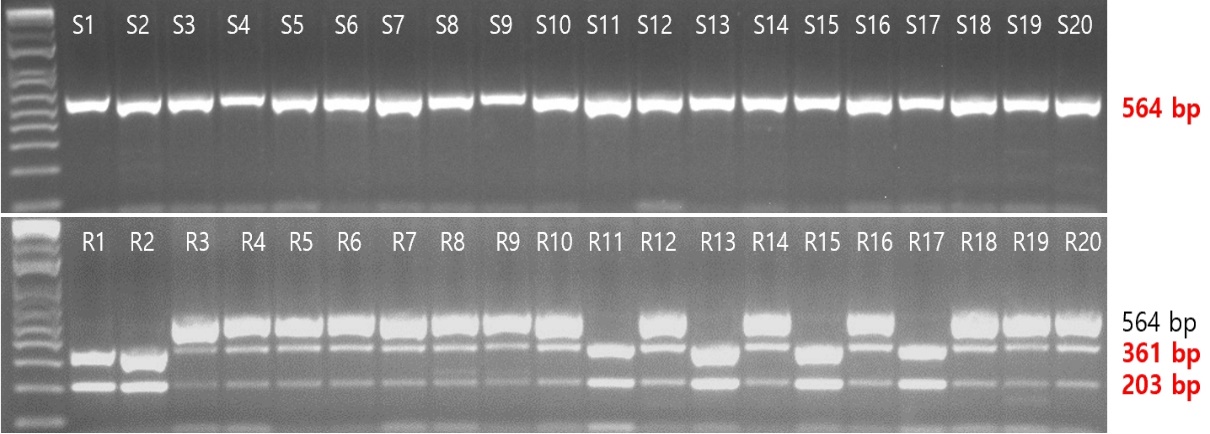 |
| PMC_55  (PCR) | 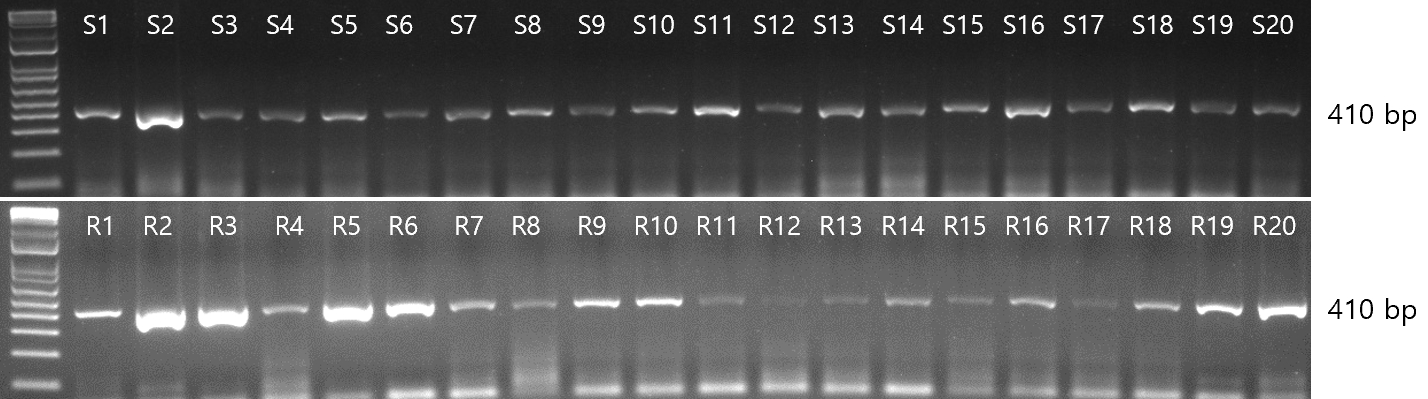 |
| PMC_55  (RE) | 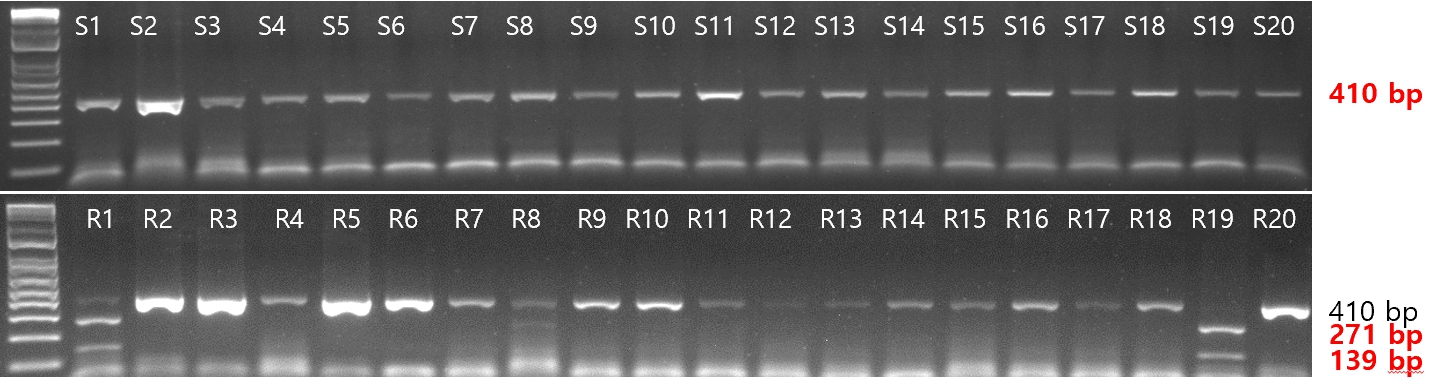 |
| PMC_71  (PCR) | 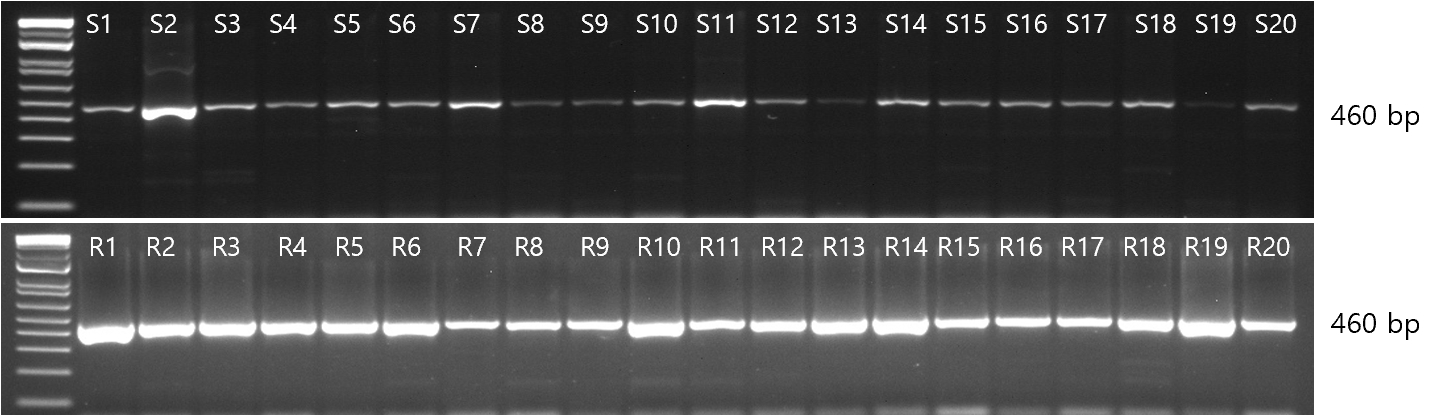 |
| PMC_71  (RE) | 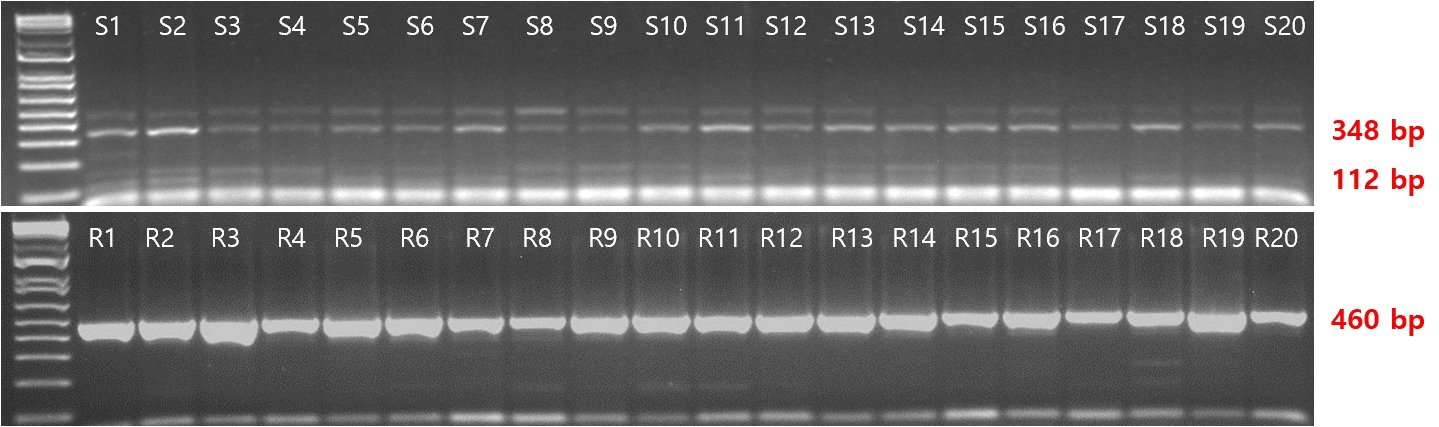 |
| PMC_72  (PCR) | 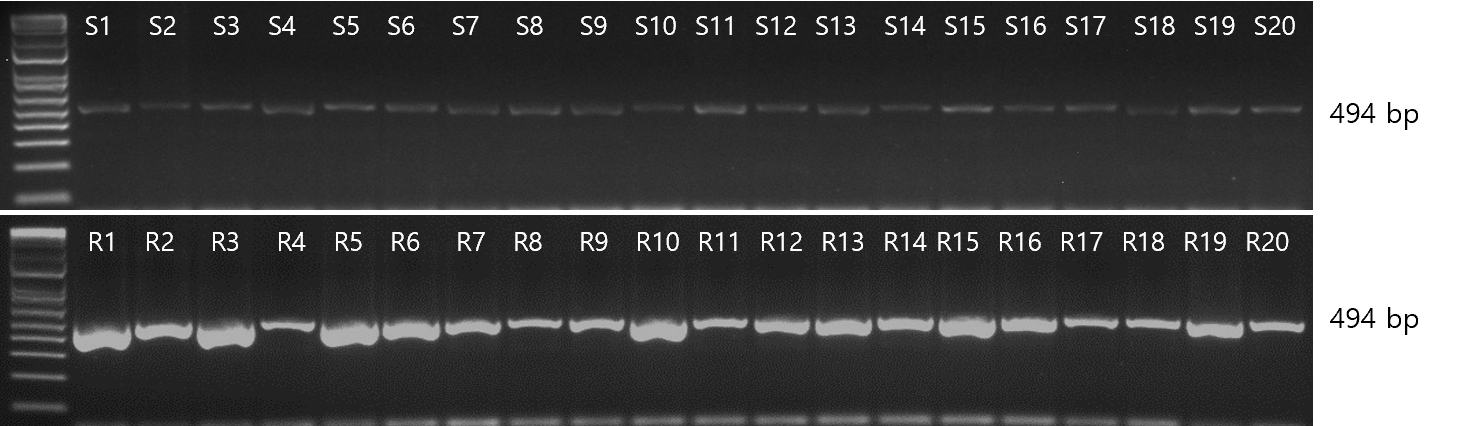 |
| PMC_72  (RE) | 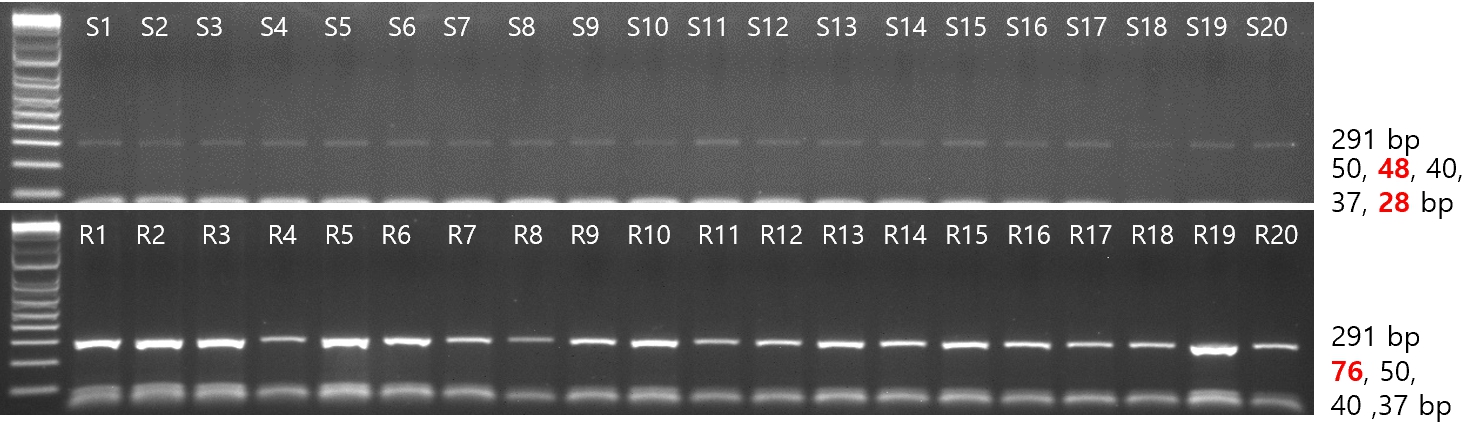 |
| PMC_75  (PCR) | 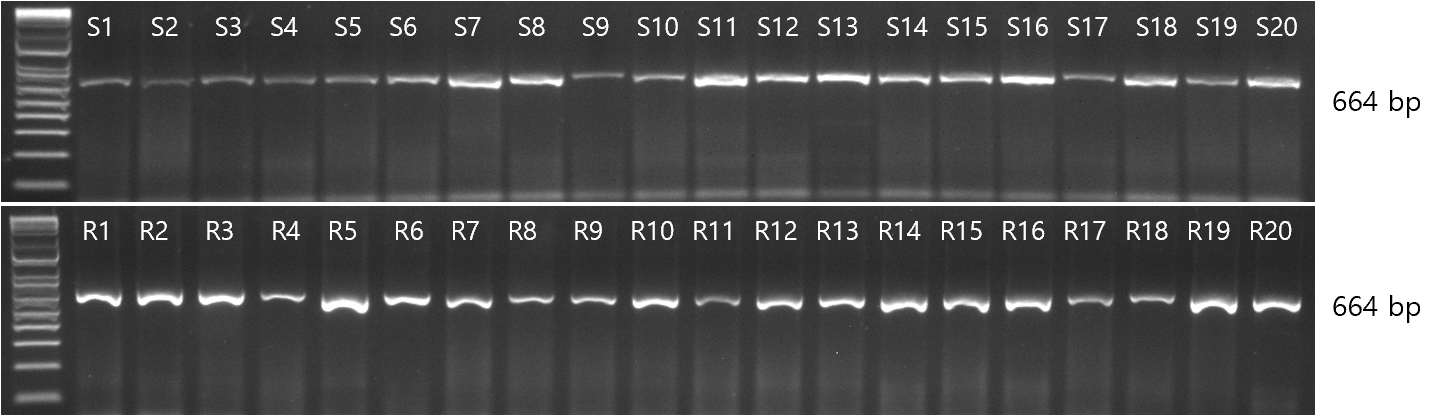 |
| PMC_75  (RE) | 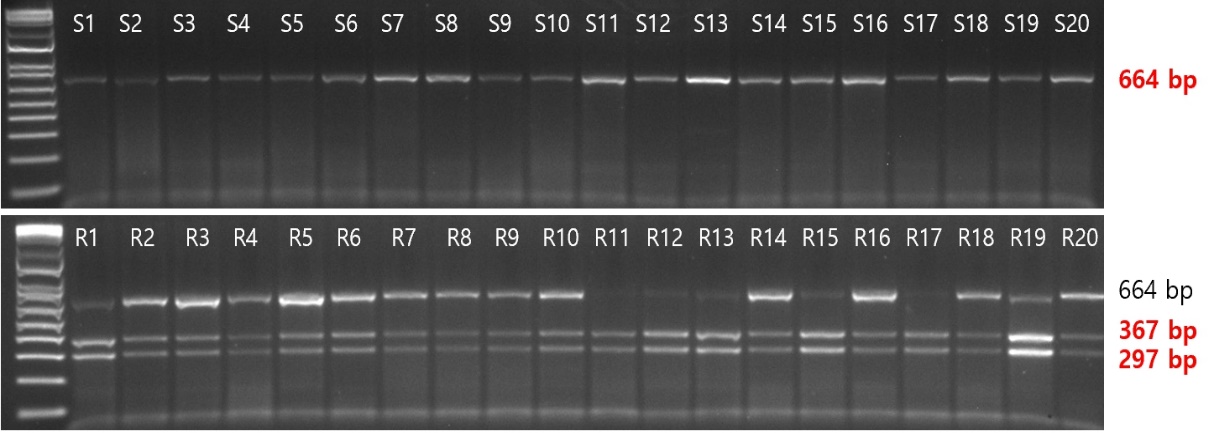 |
| PMC_76  (PCR) | 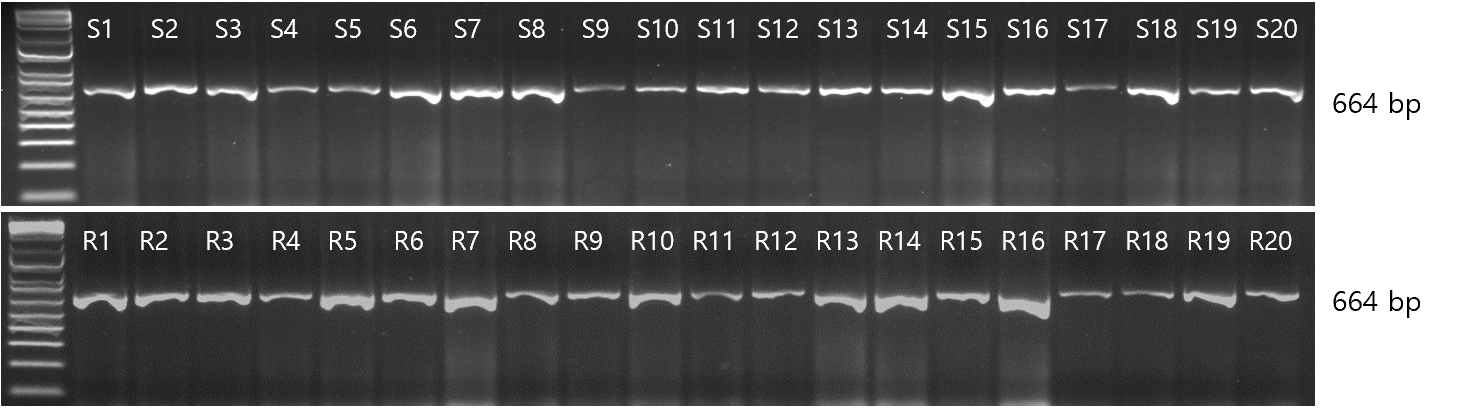 |
| PMC_76  (RE) | 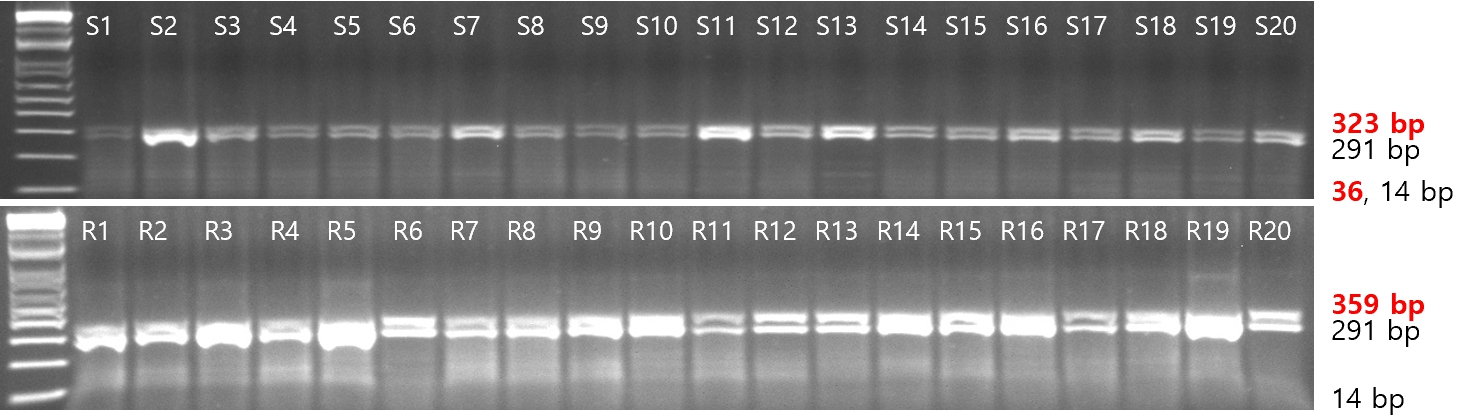 |
| PMC_80  (PCR) | 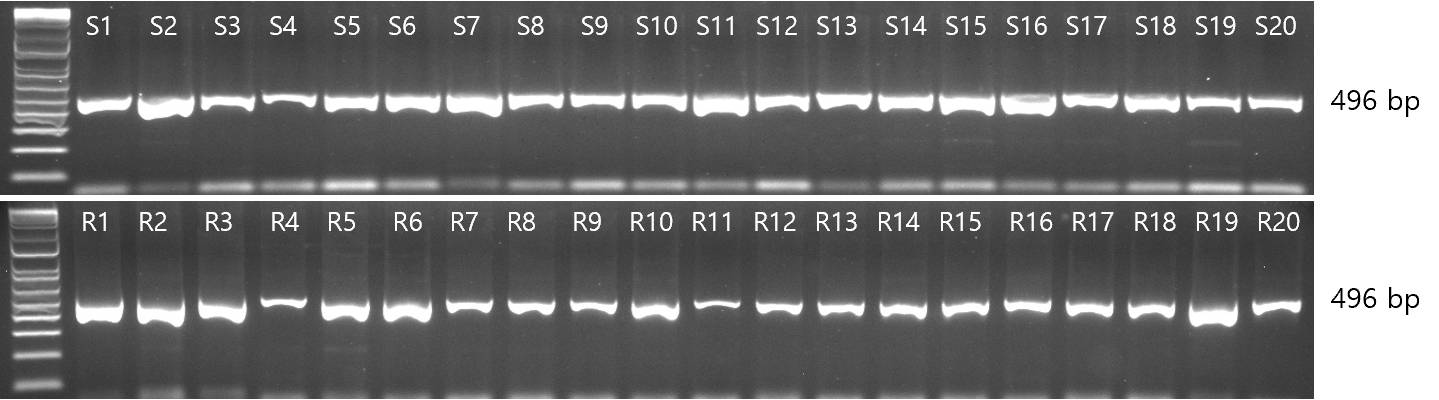 |
| PMC_80  (RE) | 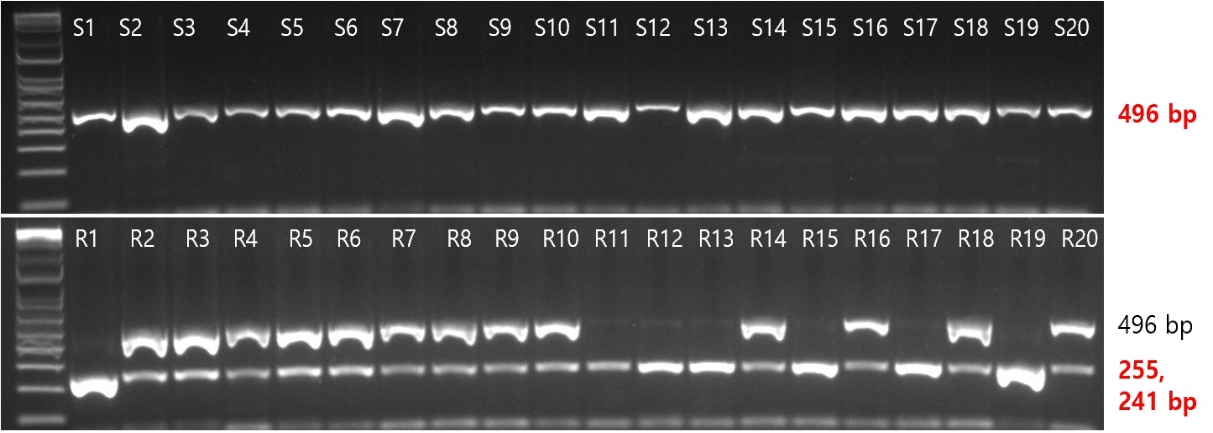 |
| PMC_106  (PCR) | 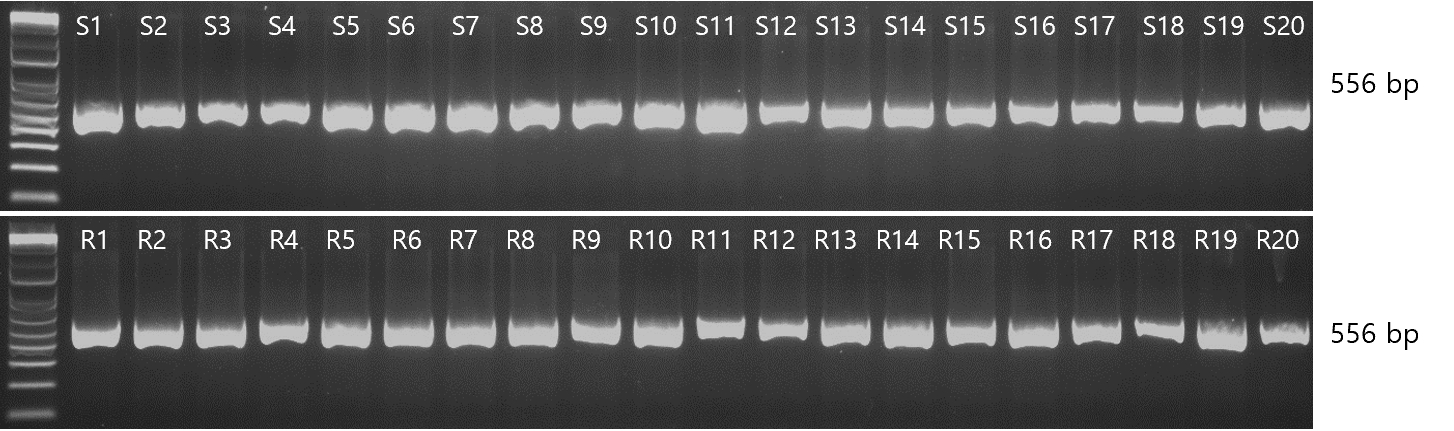 |
| PMC_106  (RE) | 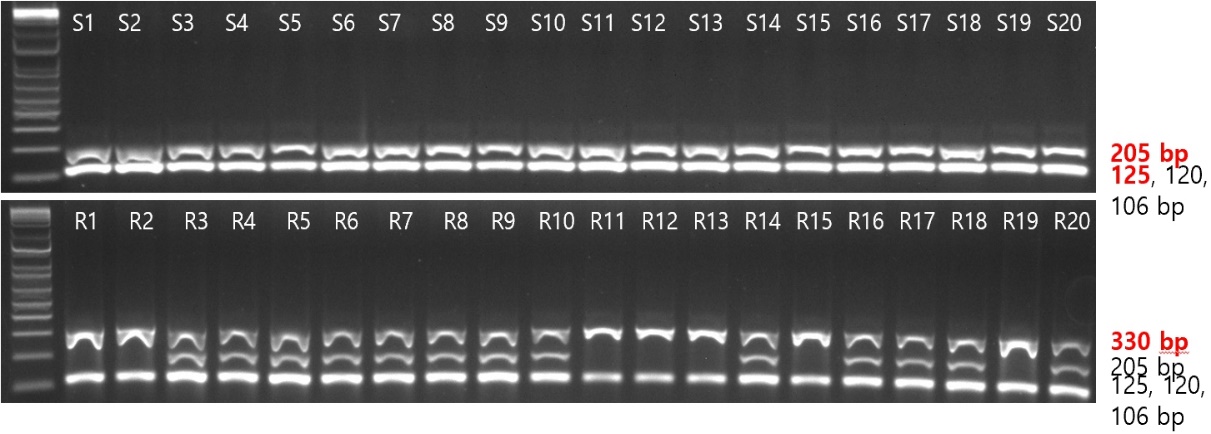 |
| PMC_114  (PCR) | 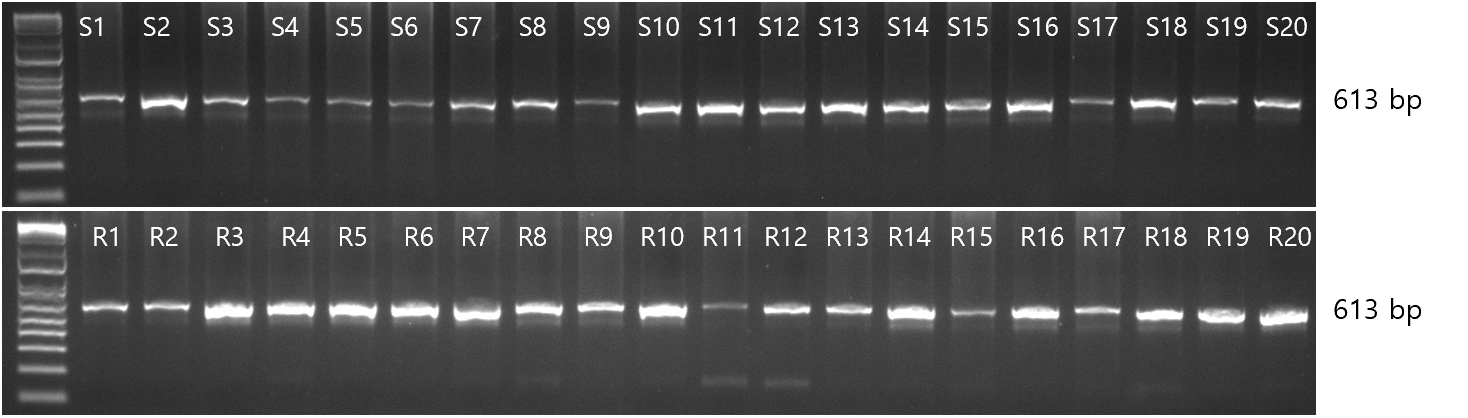 |
| PMC_114  (RE) | 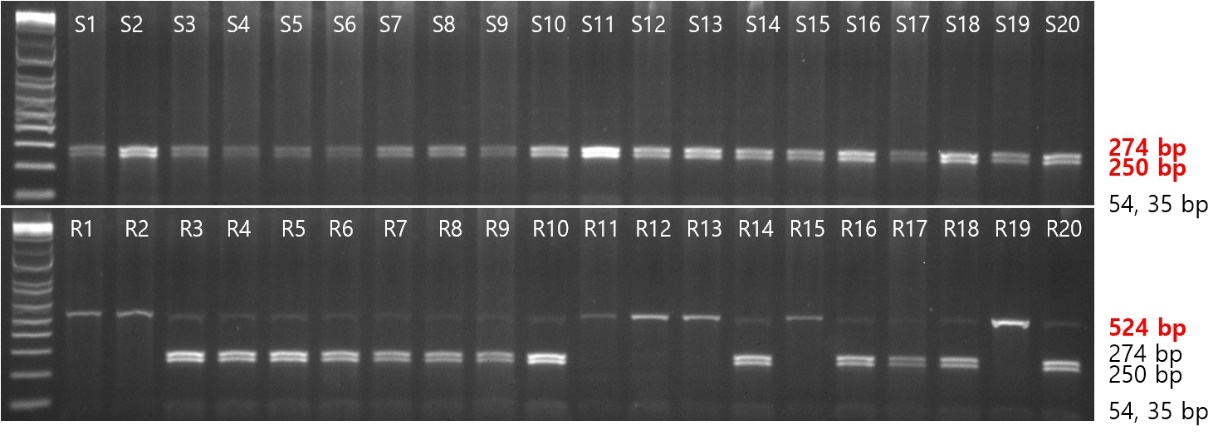 |
| PMC_121  (PCR) | 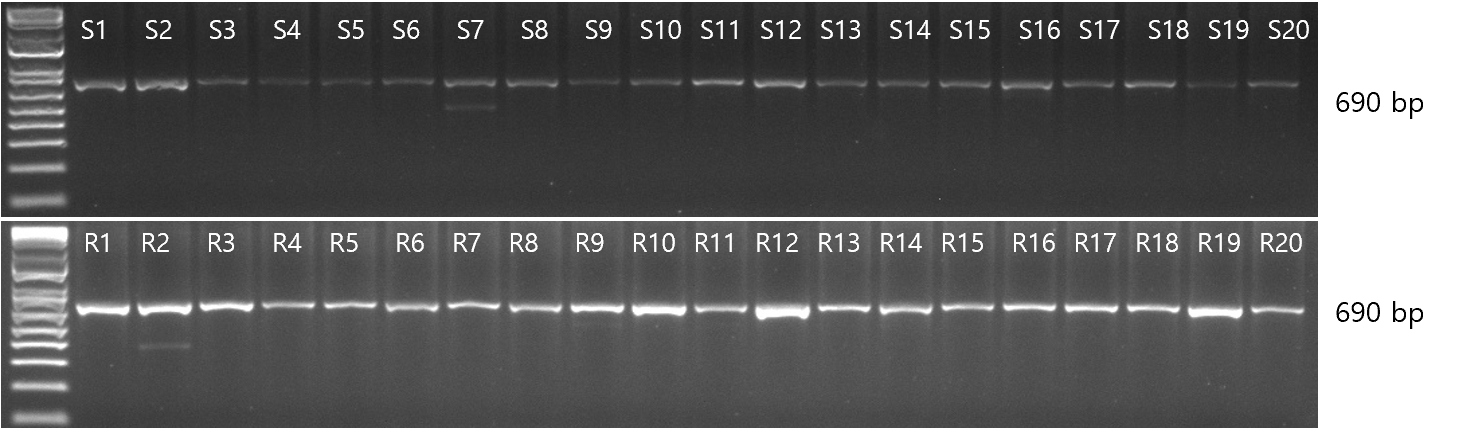 |
| PMC_121  (RE) | 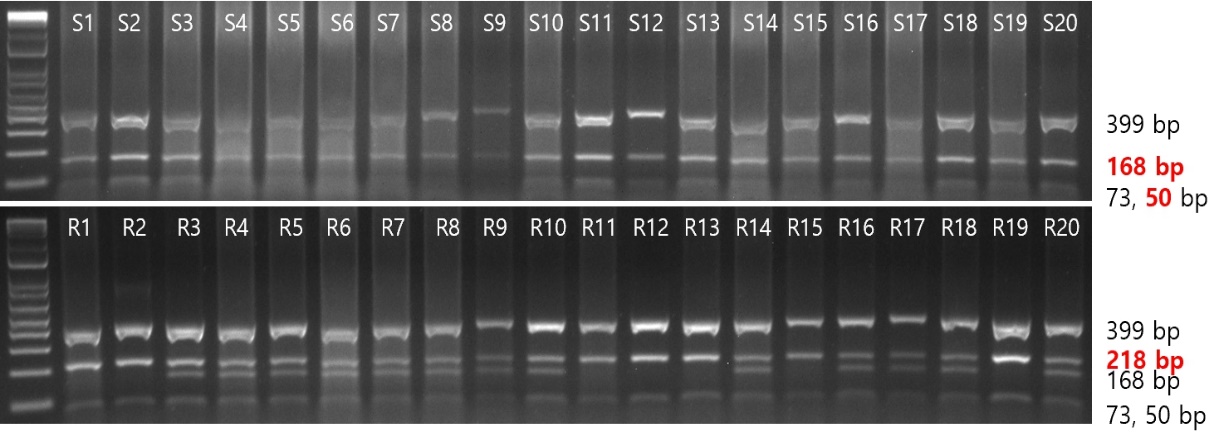 |
| PMC_129  (PCR) | 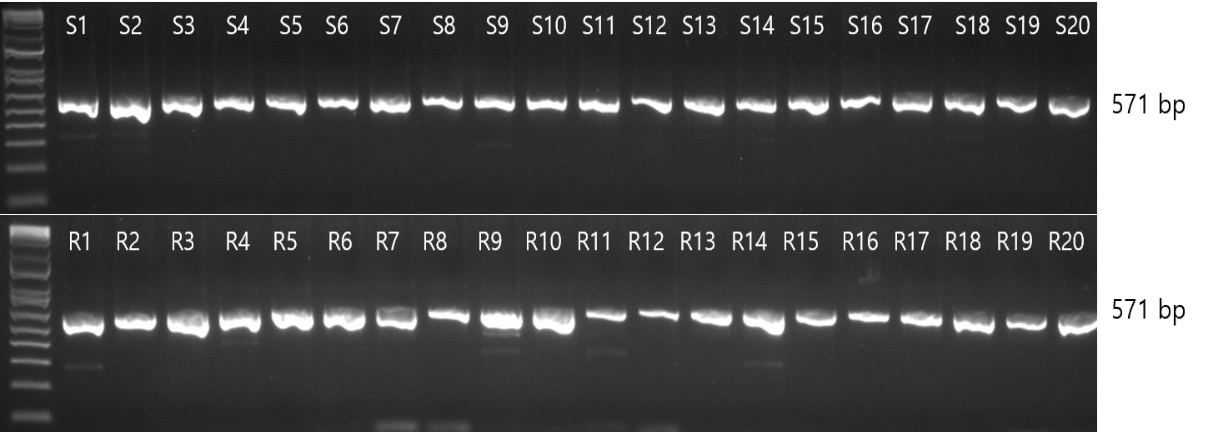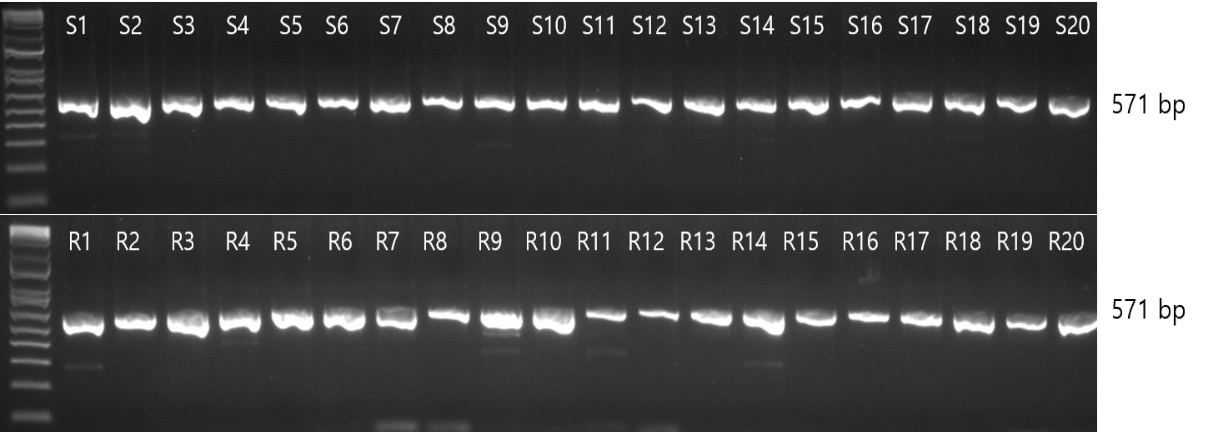 |
| PMC_129  (RE) | 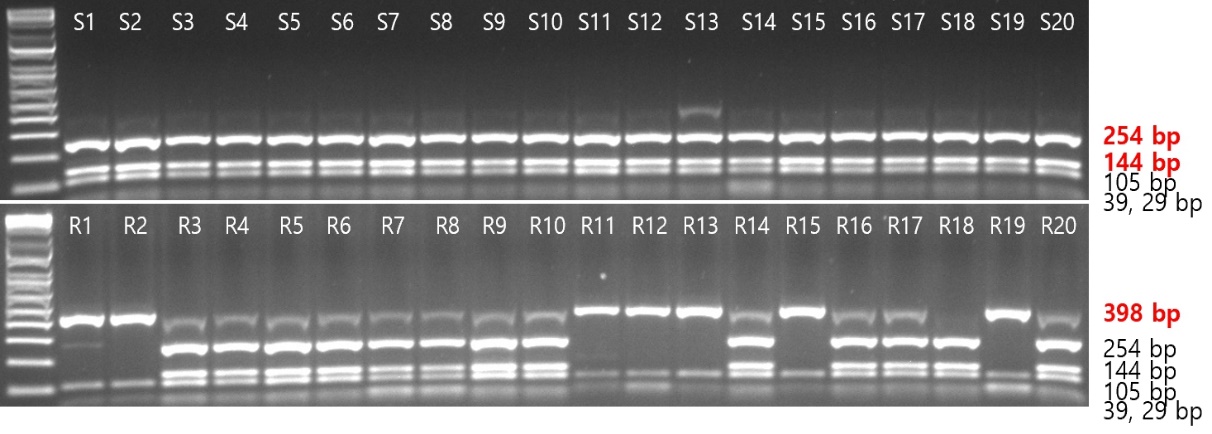 |
| PMC_130  (PCR) | 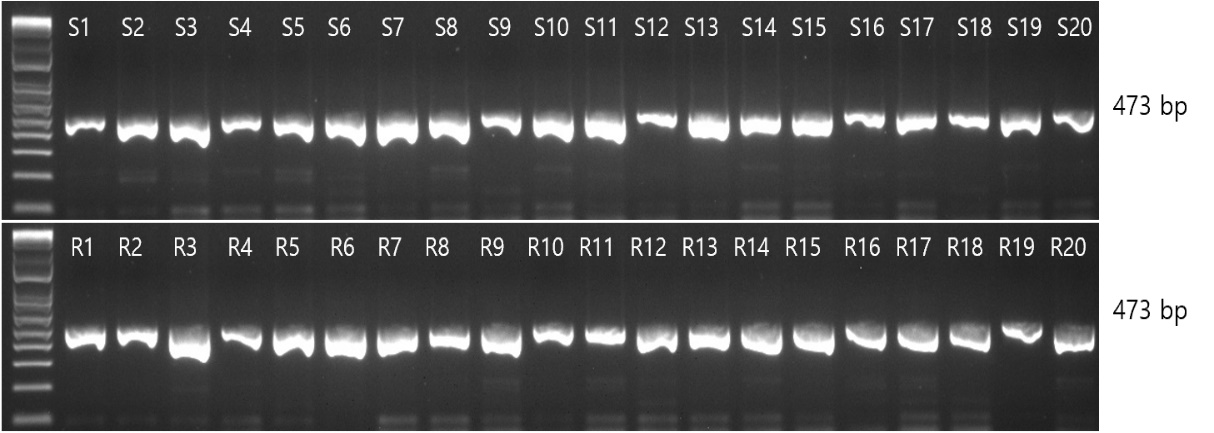 |
| PMC_130  (RE) | 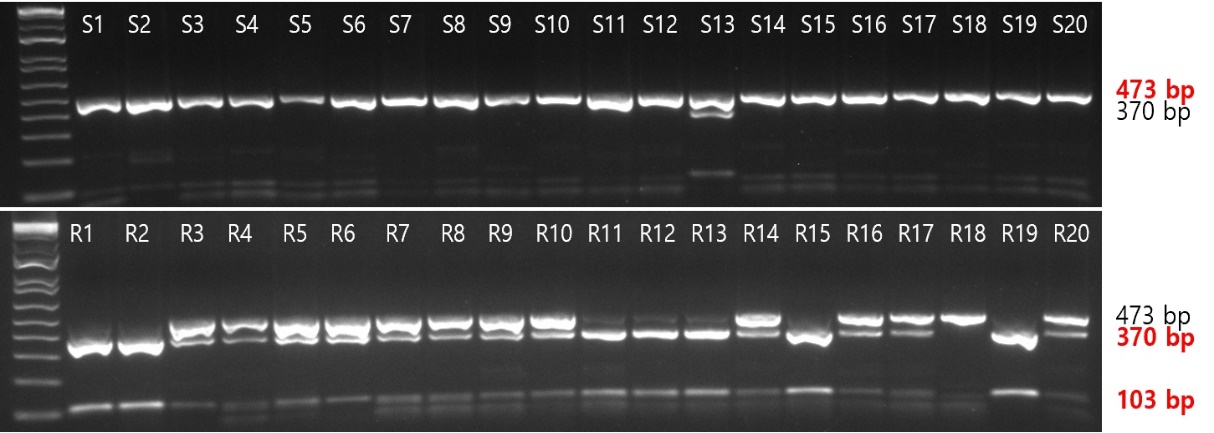 |

**Supplementary Figure 6** Validation of 19 cleaved amplified polymorphic sequence markers in susceptible and resistant F2 individuals. The red letter indicates the size determining the band pattern differences between susceptible and resistant individuals, RE; restriction enzyme.

| PMC_4  (top: PCR, down: RE) | 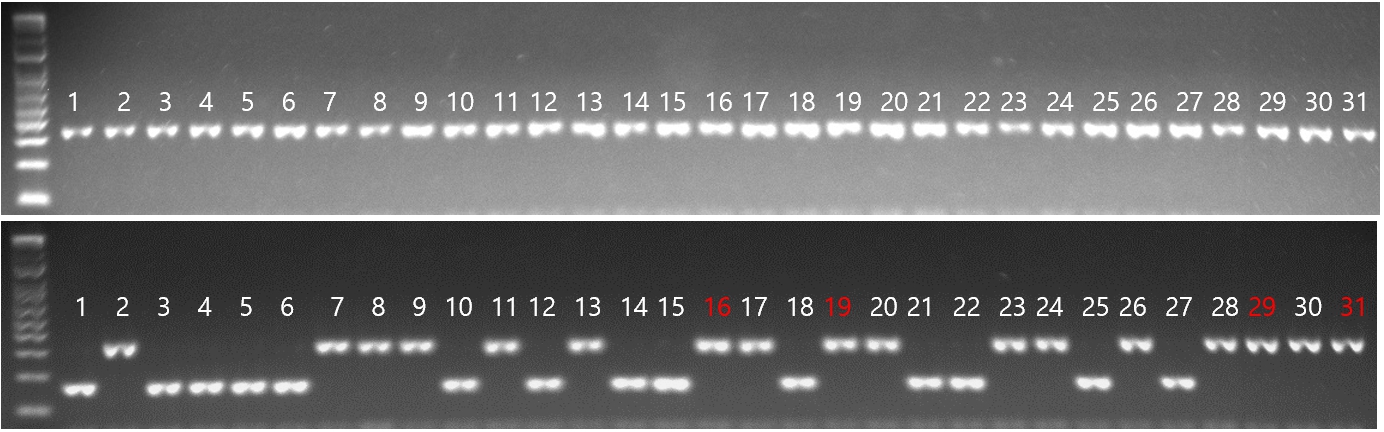 |
| --- | --- |
| PMC_23  (top: PCR, down: RE) | 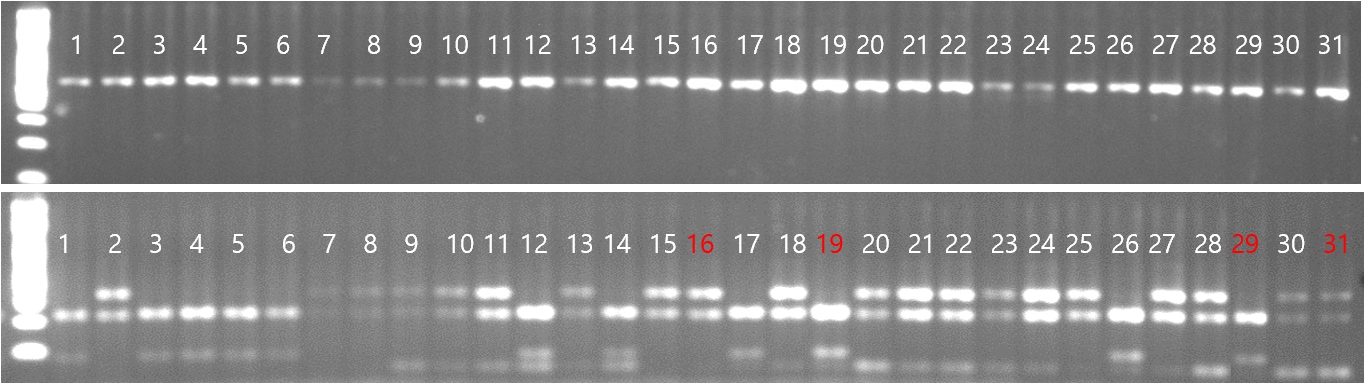 |
| PMC_28  (top: PCR, down: RE) | 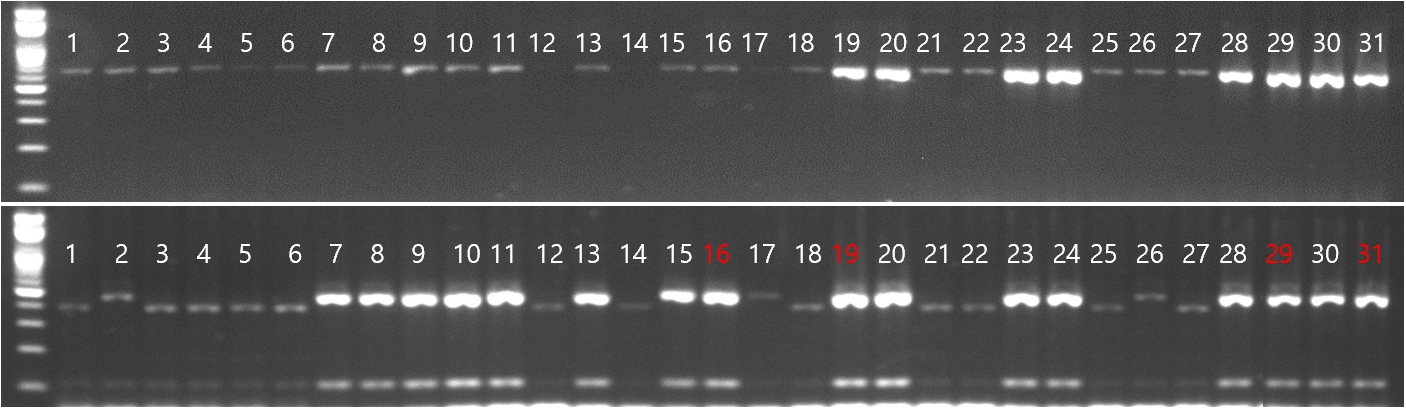 |
| PMC_30  (top: PCR, down: RE) | 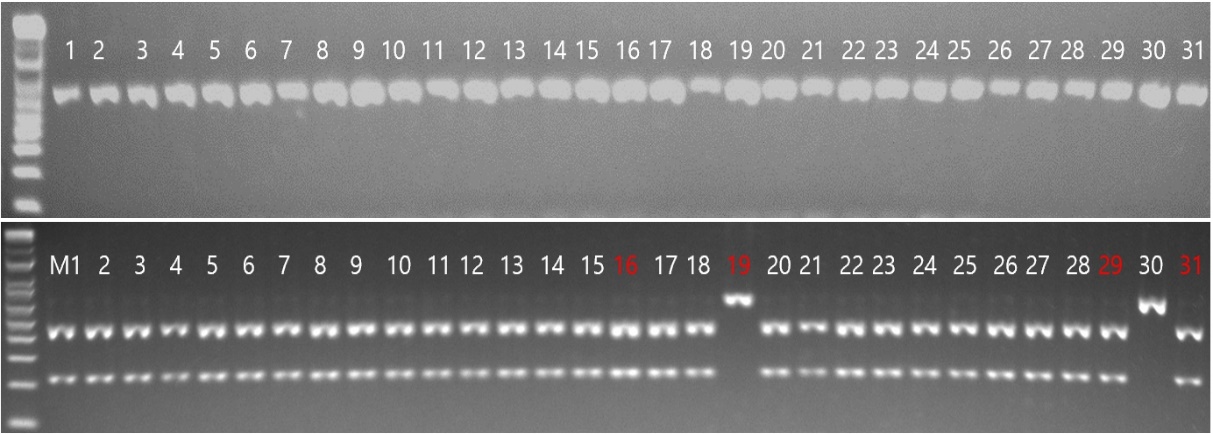 |
| PMC_71  (top: PCR, down: RE) | 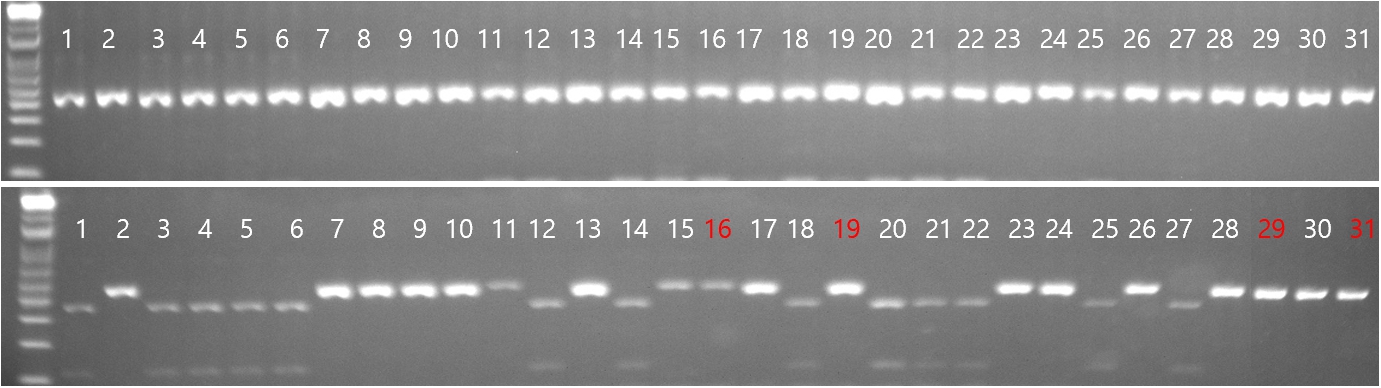 |
| PMC_72  (top: PCR, down: RE) | 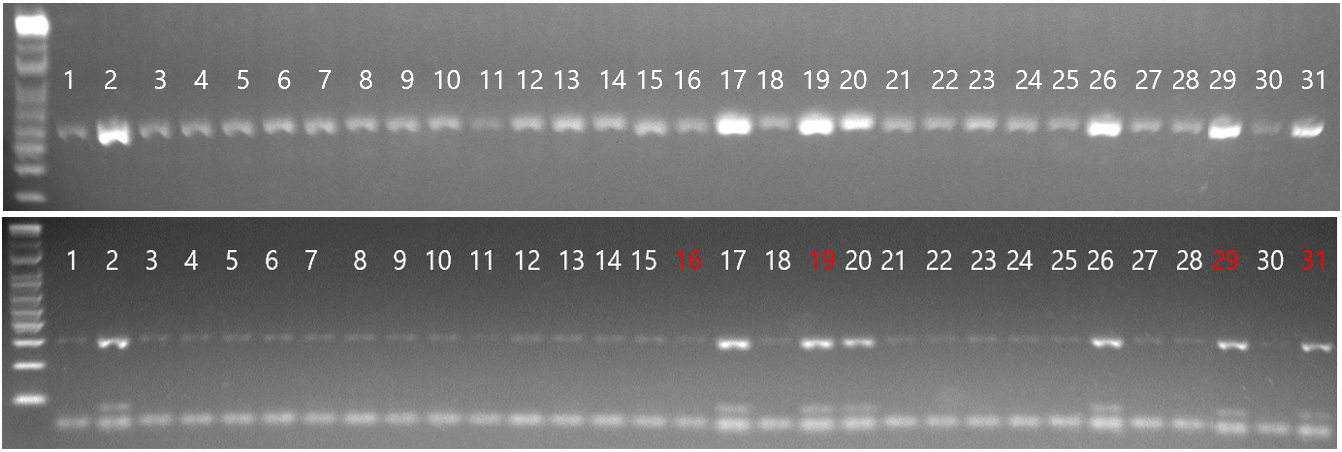 |
| PMC_75  (top: PCR, down: RE) | 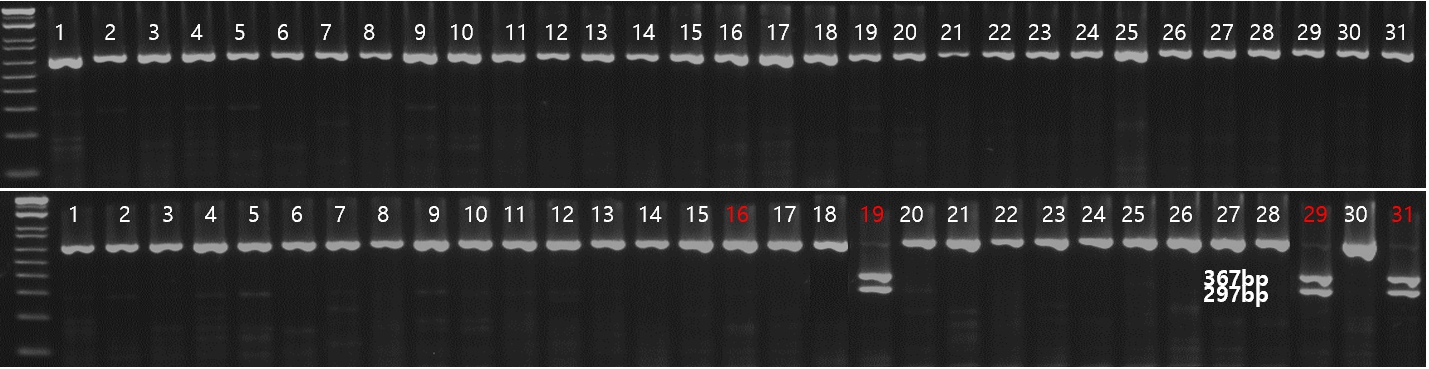 |
| PMC_76  (top: PCR, down: RE) | 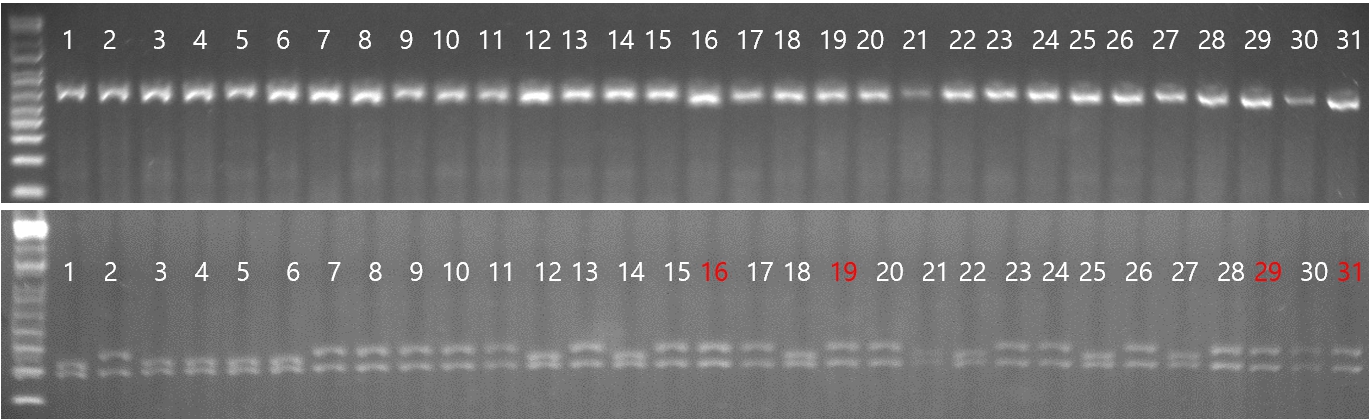 |
| PMC_80  (top: PCR, down: RE) | 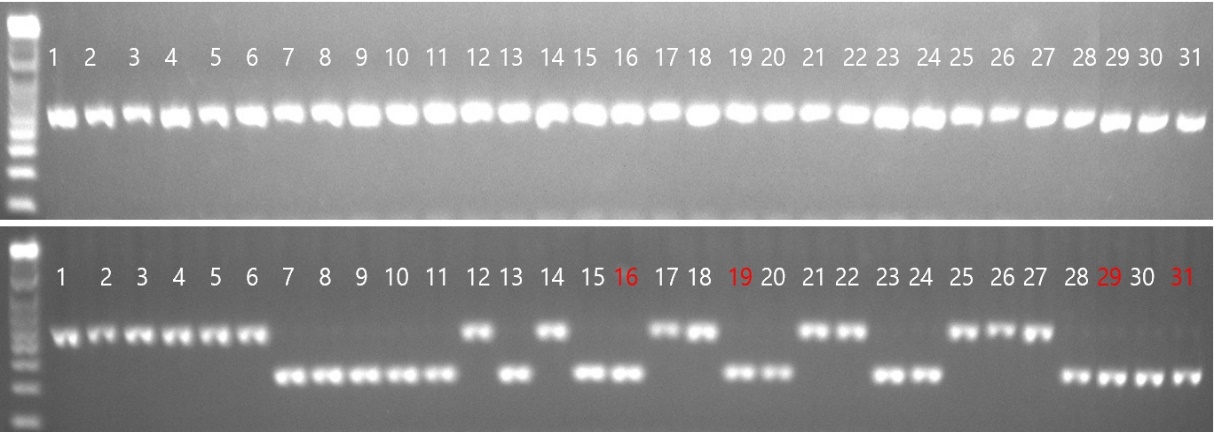 |
| PMC_106  (top: PCR, down: RE) | 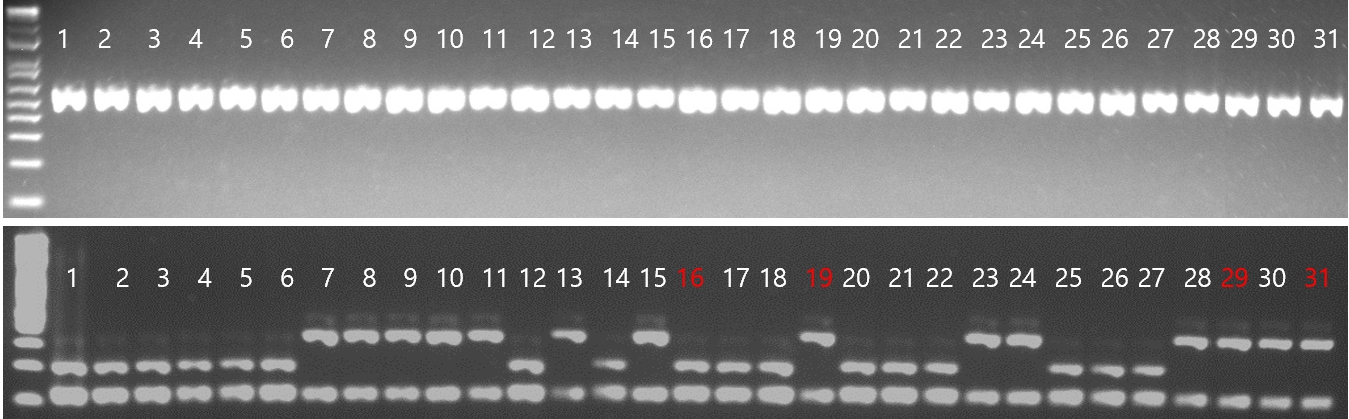 |
| PMC_114  (top: PCR, down: RE) | 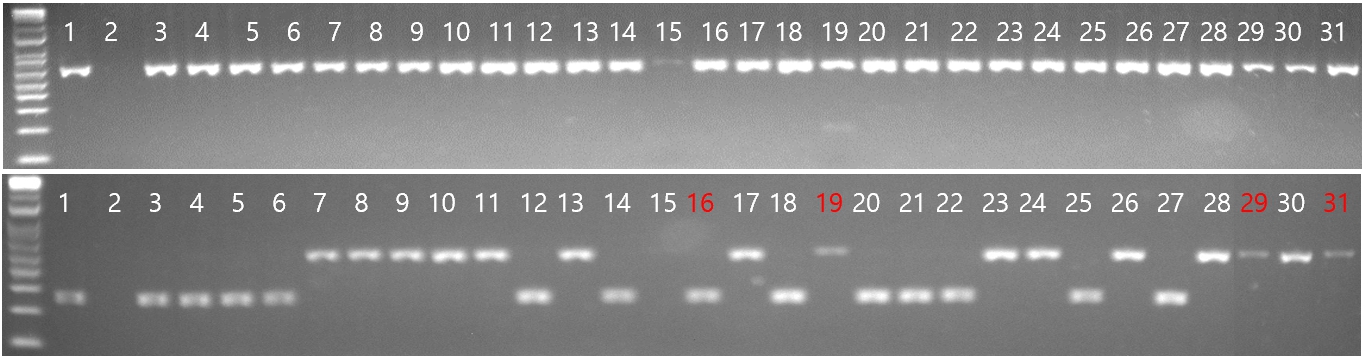 |
| PMC_121  (top: PCR, down: RE) | 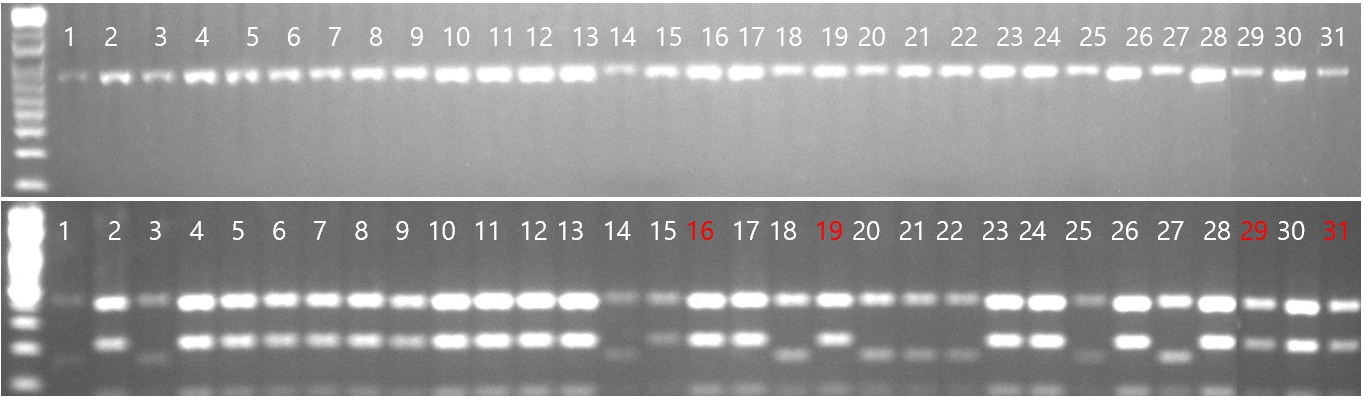 |

**Supplementary Figure 7** Validation of 12 cleaved amplified polymorphic sequence markers in 31 Korean malting barley cultivars. The red letter indicates resistant cultivars. 16; ‘Dajin,’ 19; ‘Baegho,’ 29; ‘Joongmo2014,’ 31; ‘Gangmaeg.’
