# Supplementary figures and images for: Development of cleaved amplified polymorphic sequence marker for powdery mildew resistance in Korean malting barley using QTL-seq
Source: Front Plant Sci. 2025 May 12;16:1596811. doi: 10.3389/fpls.2025.1596811 (PMC12104202; doi:10.3389/fpls.2025.1596811)

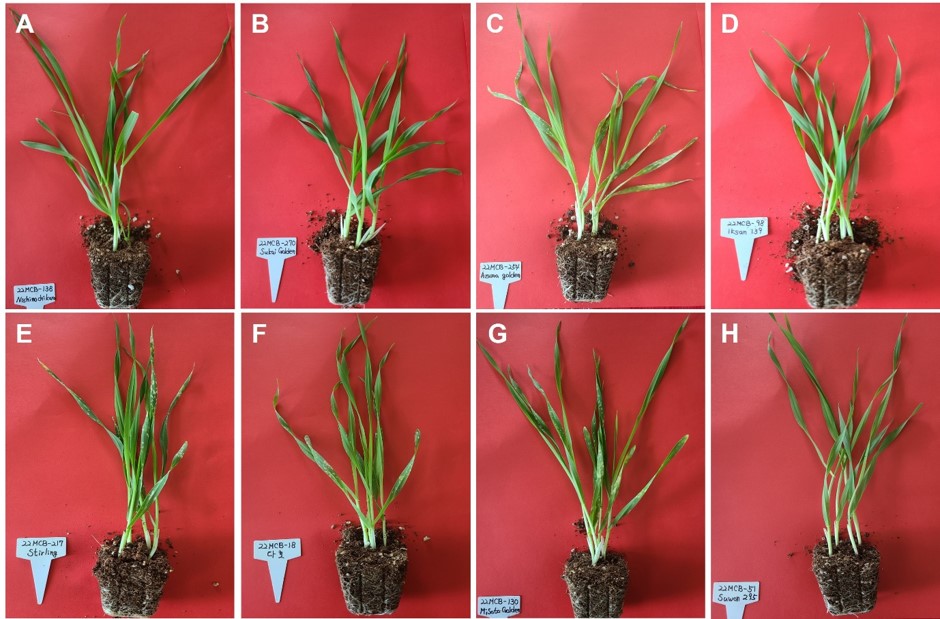

Supplement: Supplementary file 1 [file DataSheet1.zip › Supplementary Figure 8.jpg]

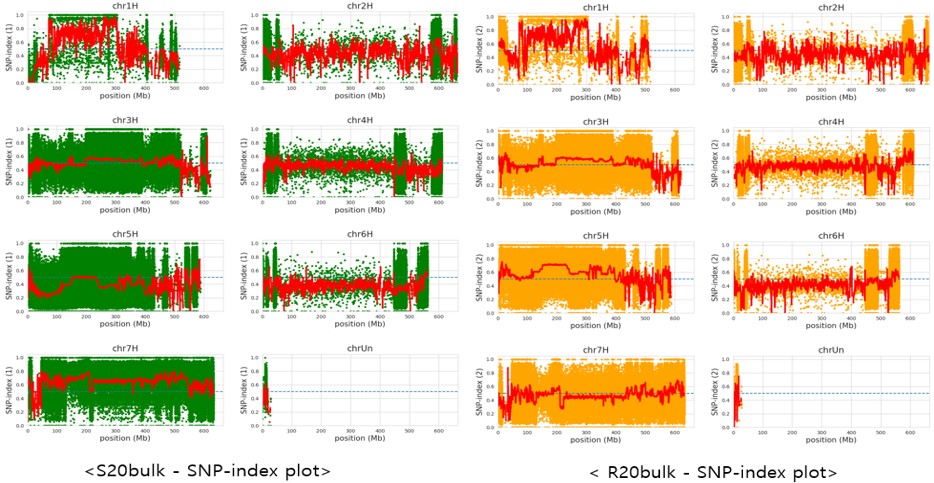

Supplement: Supplementary file 1 [file DataSheet1.zip › Supplementary Figure 1.jpg]

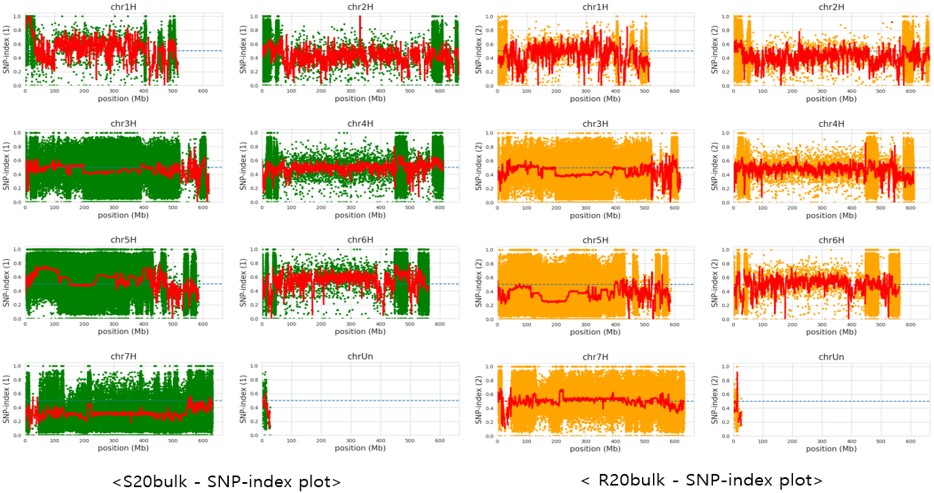

Supplement: Supplementary file 1 [file DataSheet1.zip › Supplementary Figure 2.jpg]

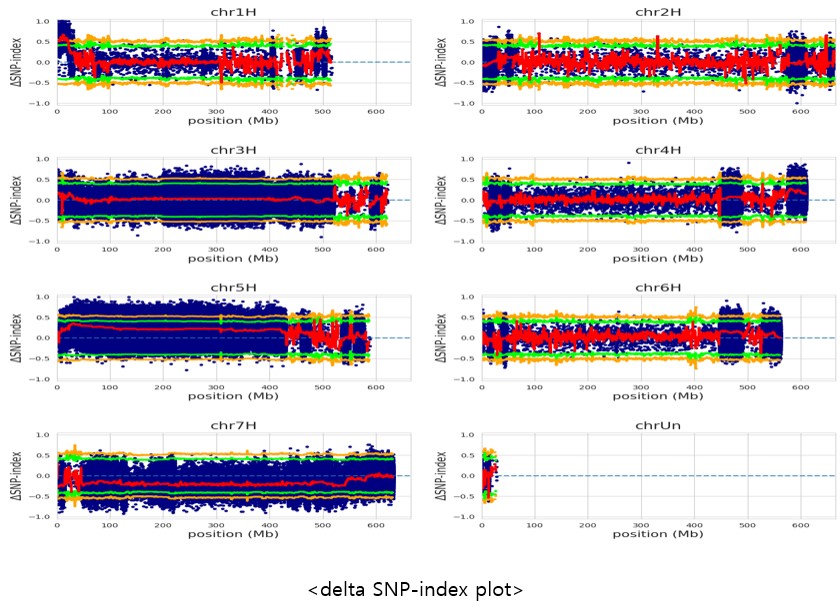

Supplement: Supplementary file 1 [file DataSheet1.zip › Supplementary Figure 3.jpg]

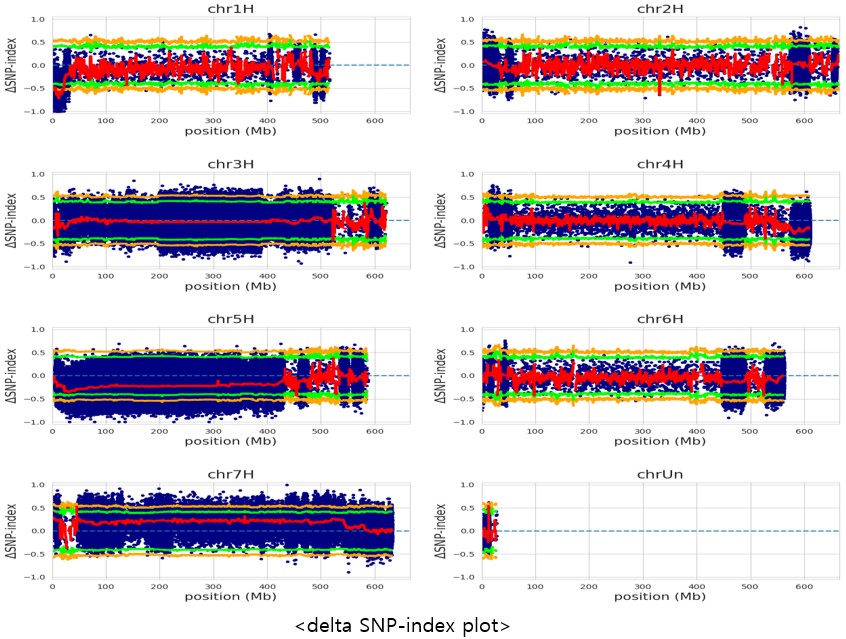

Supplement: Supplementary file 1 [file DataSheet1.zip › Supplementary Figure 4.jpg]
