# Supplementary material for: Development of cleaved amplified polymorphic sequence marker for powdery mildew resistance in Korean malting barley using QTL-seq
Source: Front Plant Sci. 2025 May 12;16:1596811. doi: 10.3389/fpls.2025.1596811 (PMC12104202; doi:10.3389/fpls.2025.1596811)
Supplement: Supplementary file 2 [file DataSheet2.docx]

***Supplementary information***

**Supplementary Figure 1** Single nucleotide polymorphism (SNP)-index plot generated using a sliding window approach (Hopum-S20 vs R20). Green/Orange dot: SNP index, Red line: Sliding window average of SNP index.

**Supplementary Figure 2** Single nucleotide polymorphism (SNP)-index plot generated using a sliding window approach (Jeonju182-S20 vs R20). Green/Orange dot: SNP index, Red line: Sliding window average of SNP index.

**Supplementary Figure 3** ΔSingle nucleotide polymorphism (SNP)-index plots (Hopum-S20 vs. R20) between bulk1 and bulk2. Blue dot: ΔSNP-index, red line: sliding window average of ΔSNP-index with a 2-Mb interval and a window size of 100 kb, green lines: sliding window average of 95% confidence interval upper/lower side (*p* < 0.05), orange lines: sliding window average of 99% confidence interval upper/lower side (*p* < 0.01), sliding window size: 2-Mb window size and 100-kb increment.

**Supplementary Figure 4** ΔSingle nucleotide polymorphism (SNP)-index plots (Jeonju182-S20 vs. R20) between bulk1 and bulk2. Blue dot: ΔSNP-index, red line: sliding window average of ΔSNP-index with a 2-Mb interval and a window size of 100 kb, green lines: sliding window average of 95% confidence interval upper/lower side (*p* < 0.05), orange lines: sliding window average of 99% confidence interval upper/lower side (*p* < 0.01), sliding window size: 2 Mb window size and 100-kb increment.

**Supplementary Figure 5** Validation of 21 cleaved amplified polymorphic sequence markers in parental lines and bulk samples.

**Supplementary Figure 6** Validation of 19 cleaved amplified polymorphic sequence markers in susceptible and resistant F_2_ individuals. The red letter indicates the size determining the band pattern differences between susceptible and resistant individuals, RE; restriction enzyme.

**Supplementary Figure 7** Validation of 12 cleaved amplified polymorphic sequence markers in 31 Korean malting barley cultivars. The red letter indicates resistant cultivars. 16; ‘Dajin,’ 19; ‘Baegho,’ 29; ‘Joongmo2014,’ 31; ‘Gangmaeg.’

**Supplementary Figure 8** Validation of powdery mildew infection in different malting barley cultivars. (A) ‘Nishinochikara’ (resistant), (B) ‘Sukai Golden’ (resistant), (C) ‘Azuma Golden’ (susceptible), (D) ‘Iksan139’ (resistant), (E) ‘Stirling’ (susceptible), (F) ‘Daho’ (susceptible), (G) ‘Misato Golden’ (susceptible), (H) ‘Suwon295’ (resistant).

**Supplementary Table 1** Variants identified in Hopum-HvPMS20 vs R20.

**Supplementary Table 2** Variants identified in Jeonju182-HvPMS20 vs R20.

**Supplementary Table 3** Statistically significant sliding window regions in Hopum-HvPMS20 vs. R20 (*p* < 0.01). POSI, central position of window (window size = 2 Mb, step = 100 kb). Mean_p99, p95, the mean absolute value of the single nucleotide polymorphism (SNP) index at the 99%, 95% confidence interval upper/lower limits (or the mean absolute value of the SNP index corresponding to *p* = 0.01, 0.05) was used as a threshold. SNPs with an absolute mean SNP index exceeding this value were considered significant at a significance level of 0.01, 0.05.

**Supplementary Table 4** Statistically significant sliding window regions in Jeonju182-HvPMS20 vs. R20 (*p* < 0.01). POSI, central position of window (window size = 2 Mb, step = 100 kb). Mean_p99, p95, the mean absolute value of the single nucleotide polymorphism (SNP) index at the 99%, 95% confidence interval upper/lower limits (or the mean absolute value of the SNP index corresponding to *p* = 0.01, 0.05) was used as a threshold. SNPs with an absolute mean SNP index exceeding this value were considered significant at a significance level of 0.01, 0.05.

**Suppelmentary Table 5** Candidate quantitative trait loci (QTL) regions and common variants identified in two QTL-seq analyses.

**Supplementary Table 6** Variant information identified through filtering within candidate quantitative trait loci regions.

**Supplementary Table 7** List of 134 cleaved amplified polymorphic sequence markers designed based on variant analysis within candidate quantitative trait loci (QTL) regions. The markers shaded in gray represent non-synonymous single nucleotide polymorphism (SNP) variants.

**Supplementary Table 8** Predicted candidate genes and their functions associated with 21 non-synonymous single nucleotide polymorphism (SNP) markers. nSNP refers to a non-synonymous SNP.
