# Supplementary material for: Development of cleaved amplified polymorphic sequence marker for powdery mildew resistance in Korean malting barley using QTL-seq
Source: Front Plant Sci. 2025 May 12;16:1596811. doi: 10.3389/fpls.2025.1596811 (PMC12104202; doi:10.3389/fpls.2025.1596811)
Supplement: Supplementary file 3 [file Table1.docx]

***Supplementary Tables***

**Supplementary Table 1** Variants identified in Hopum-HvPMS20 vs R20.

| Chromosome no. | No. of variants | | | No. of  comparable  variants |
| --- | --- | --- | --- | --- |
|  | Hopum | HvPM_S20 | HvPM_R20 |  |
| Chr1H | 1,085,047 | 1,003,563 | 1,004,546 | 31,807 |
| Chr2H | 1,717,237 | 1,772,300 | 1,756,037 | 52,633 |
| Chr3H | 1,730,667 | 1,582,915 | 1,544,546 | 520,634 |
| Chr4H | 832,002 | 915,147 | 904,090 | 86,589 |
| Chr5H | 1,681,405 | 2,188,675 | 2,116,621 | 740,229 |
| Chr6H | 1,033,119 | 1,118,622 | 1,111,081 | 73,132 |
| Chr7H | 2,154,326 | 2,319,348 | 2,358,833 | 333,334 |
| ChrUn | 11,533 | 11,288 | 11,061 | 458 |
| Total | **10,245,336** | **10,911,858** | **10,806,815** | **1,838,816** |

**Supplementary Table 2** Variants identified in Jeonju182-HvPMS20 vs R20.

| Chromosome no. | No. of variants | | | No. of  comparable  variants |
| --- | --- | --- | --- | --- |
|  | Jeonju182 | HvPM_S20 | HvPM_R20 |  |
| Chr1H | 974,605 | 1,001,194 | 1,008,474 | 26,280 |
| Chr2H | 1,706,163 | 1,772,333 | 1,755,582 | 49,912 |
| Chr3H | 566,271 | 1,445,385 | 1,417,225 | 648,771 |
| Chr4H | 842,100 | 915,424 | 908,942 | 84,544 |
| Chr5H | 1,582,313 | 2,118,916 | 2,121,972 | 763,221 |
| Chr6H | 1,041,366 | 1,118,257 | 1,115,942 | 82,821 |
| Chr7H | 1,964,224 | 2,304,140 | 2,310,583 | 302,524 |
| ChrUn | 7,878 | 10,634 | 10,544 | 843 |
| Total | **8,684,920** | **10,686,283** | **10,649,264** | **1,958,916** |

**Supplementary Table 3** Statistically significant sliding window regions in Hopum-HvPMS20 vs. R20 (*p < 0.01*). POSI, central position of window (window size = 2 Mb, step = 100 kb). Mean_p99, p95, the mean absolute value of the single nucleotide polymorphism (SNP) index at the 99%, 95% confidence interval upper/lower limits (or the mean absolute value of the SNP index corresponding to *p = 0.01, 0.05*) was used as a threshold. SNPs with an absolute mean SNP index exceeding this value were considered significant at a significance level of 0.01, 0.05.

| **CHROM** | **POSI** | **mean_p99** | **mean_p95** | **mean_SNP**  **index1** | **mean_SNP**  **index2** | **mean_SNP**  **index** |
| --- | --- | --- | --- | --- | --- | --- |
| chr1H | 3500000 | 0.518 | 0.401 | 0.0813 | 0.6 | 0.5187 |
| chr1H | 6800000 | 0.5303 | 0.4089 | 0.0315 | 0.5653 | 0.5338 |
| chr1H | 6900000 | 0.5305 | 0.409 | 0.0293 | 0.5818 | 0.5525 |
| chr1H | 7000000 | 0.5335 | 0.4114 | 0.0299 | 0.5991 | 0.5692 |
| chr1H | 7100000 | 0.5342 | 0.4119 | 0.0287 | 0.604 | 0.5754 |
| chr1H | 7200000 | 0.5336 | 0.4115 | 0.0272 | 0.6056 | 0.5784 |
| chr1H | 7300000 | 0.5351 | 0.4128 | 0.0259 | 0.6056 | 0.5798 |
| chr1H | 7400000 | 0.5361 | 0.4137 | 0.0243 | 0.6092 | 0.5849 |
| chr1H | 7500000 | 0.5375 | 0.4151 | 0.0231 | 0.6132 | 0.5901 |
| chr1H | 7600000 | 0.5366 | 0.4144 | 0.022 | 0.6152 | 0.5932 |
| chr1H | 7700000 | 0.537 | 0.4149 | 0.0214 | 0.6187 | 0.5972 |
| chr1H | 7800000 | 0.5379 | 0.416 | 0.0182 | 0.63 | 0.6118 |
| chr1H | 7900000 | 0.5378 | 0.4161 | 0.0164 | 0.6298 | 0.6134 |
| chr1H | 8000000 | 0.539 | 0.4169 | 0.0142 | 0.6319 | 0.6177 |
| chr1H | 8100000 | 0.5388 | 0.4167 | 0.0141 | 0.6335 | 0.6194 |
| chr1H | 8200000 | 0.5393 | 0.4171 | 0.0138 | 0.6316 | 0.6178 |
| chr1H | 8300000 | 0.54 | 0.4174 | 0.0135 | 0.6294 | 0.616 |
| chr1H | 8400000 | 0.5381 | 0.4158 | 0.0126 | 0.6239 | 0.6112 |
| chr1H | 8500000 | 0.5369 | 0.4151 | 0.011 | 0.6134 | 0.6024 |
| chr1H | 8600000 | 0.5385 | 0.4165 | 0.01 | 0.6144 | 0.6044 |
| chr1H | 8700000 | 0.5397 | 0.4174 | 0.009 | 0.6157 | 0.6067 |
| chr1H | 8800000 | 0.54 | 0.4176 | 0.0056 | 0.6136 | 0.608 |
| chr1H | 8900000 | 0.5418 | 0.4191 | 0.0021 | 0.6081 | 0.606 |
| chr1H | 9000000 | 0.5409 | 0.4185 | 0.002 | 0.5961 | 0.5942 |
| chr1H | 9100000 | 0.5396 | 0.4176 | 0.0019 | 0.588 | 0.5861 |
| chr1H | 9200000 | 0.5395 | 0.4173 | 0.0017 | 0.5782 | 0.5766 |
| chr1H | 9300000 | 0.5359 | 0.4145 | 0.0016 | 0.5749 | 0.5733 |
| chr1H | 9400000 | 0.5347 | 0.4135 | 0.0015 | 0.5701 | 0.5685 |
| chr1H | 9500000 | 0.534 | 0.4127 | 0.0016 | 0.5683 | 0.5667 |
| chr1H | 9600000 | 0.5352 | 0.4136 | 0.0014 | 0.5664 | 0.565 |
| chr1H | 9700000 | 0.535 | 0.4134 | 0.0014 | 0.564 | 0.5627 |
| chr1H | 9800000 | 0.5351 | 0.4134 | 0.0024 | 0.5573 | 0.5549 |
| chr1H | 9900000 | 0.5349 | 0.4132 | 0.0022 | 0.5557 | 0.5535 |
| chr1H | 10000000 | 0.5336 | 0.4122 | 0.0024 | 0.5497 | 0.5472 |
| chr1H | 10100000 | 0.5329 | 0.4117 | 0.0025 | 0.5501 | 0.5476 |
| chr1H | 10200000 | 0.5326 | 0.4115 | 0.0025 | 0.552 | 0.5496 |
| chr1H | 10300000 | 0.5331 | 0.4121 | 0.0026 | 0.5518 | 0.5492 |
| chr1H | 10400000 | 0.5356 | 0.4143 | 0.002 | 0.5486 | 0.5466 |
| chr1H | 10500000 | 0.5369 | 0.4149 | 0.0023 | 0.5524 | 0.5502 |
| chr1H | 10600000 | 0.5355 | 0.4137 | 0.0021 | 0.5546 | 0.5524 |
| chr1H | 10700000 | 0.5356 | 0.414 | 0.0027 | 0.5565 | 0.5539 |
| chr1H | 10800000 | 0.5346 | 0.4132 | 0.0027 | 0.5618 | 0.5591 |
| chr1H | 10900000 | 0.5348 | 0.4134 | 0.0026 | 0.5756 | 0.5731 |
| chr1H | 11000000 | 0.535 | 0.4137 | 0.0028 | 0.5874 | 0.5845 |
| chr1H | 11100000 | 0.5359 | 0.4144 | 0.0029 | 0.5945 | 0.5916 |
| chr1H | 11200000 | 0.5353 | 0.4141 | 0.0034 | 0.6149 | 0.6115 |
| chr1H | 11300000 | 0.5407 | 0.4183 | 0.0038 | 0.6266 | 0.6228 |
| chr1H | 11400000 | 0.5422 | 0.4196 | 0.004 | 0.6328 | 0.6288 |
| chr1H | 11500000 | 0.5418 | 0.4195 | 0.0041 | 0.6301 | 0.626 |
| chr1H | 11600000 | 0.5414 | 0.4191 | 0.0044 | 0.6351 | 0.6307 |
| chr1H | 11700000 | 0.5408 | 0.4185 | 0.0044 | 0.6361 | 0.6318 |
| chr1H | 11800000 | 0.5411 | 0.4187 | 0.002 | 0.6364 | 0.6344 |
| chr1H | 11900000 | 0.5404 | 0.4181 | 0.002 | 0.6448 | 0.6427 |
| chr1H | 12000000 | 0.5404 | 0.4181 | 0.002 | 0.6448 | 0.6427 |
| chr1H | 12100000 | 0.5427 | 0.42 | 0.0019 | 0.6486 | 0.6466 |
| chr1H | 12200000 | 0.5435 | 0.4204 | 0.002 | 0.6485 | 0.6466 |
| chr1H | 12300000 | 0.5413 | 0.4186 | 0.0016 | 0.6664 | 0.6648 |
| chr1H | 12400000 | 0.5393 | 0.4167 | 0.0013 | 0.6798 | 0.6785 |
| chr1H | 12500000 | 0.5412 | 0.4184 | 0.0026 | 0.6799 | 0.6774 |
| chr1H | 12600000 | 0.5425 | 0.4192 | 0.0027 | 0.6763 | 0.6735 |
| chr1H | 12700000 | 0.5427 | 0.4194 | 0.0018 | 0.6749 | 0.6731 |
| chr1H | 12800000 | 0.5432 | 0.4199 | 0.0019 | 0.6712 | 0.6693 |
| chr1H | 12900000 | 0.5462 | 0.4214 | 0.0021 | 0.6532 | 0.6511 |
| chr1H | 13000000 | 0.5475 | 0.4226 | 0.0046 | 0.6443 | 0.6397 |
| chr1H | 13100000 | 0.5506 | 0.425 | 0.0054 | 0.6486 | 0.6431 |
| chr1H | 13200000 | 0.545 | 0.4205 | 0.0046 | 0.6216 | 0.617 |
| chr1H | 13300000 | 0.5454 | 0.4208 | 0.0046 | 0.6207 | 0.6161 |
| chr1H | 13400000 | 0.5454 | 0.4208 | 0.0046 | 0.6207 | 0.6161 |
| chr1H | 13500000 | 0.5426 | 0.4186 | 0.0039 | 0.5989 | 0.5951 |
| chr1H | 13600000 | 0.5424 | 0.4184 | 0.0036 | 0.5935 | 0.5899 |
| chr1H | 13700000 | 0.5392 | 0.416 | 0.0042 | 0.5867 | 0.5825 |
| chr1H | 13800000 | 0.5391 | 0.4165 | 0.0032 | 0.5682 | 0.565 |
| chr1H | 13900000 | 0.5396 | 0.4167 | 0.0026 | 0.5501 | 0.5475 |
| chr1H | 14000000 | 0.5374 | 0.4151 | 0.0021 | 0.544 | 0.5419 |
| chr1H | 14100000 | 0.5374 | 0.4153 | 0.0019 | 0.545 | 0.5431 |
| chr1H | 14200000 | 0.537 | 0.415 | 0.0019 | 0.5433 | 0.5414 |
| chr1H | 14300000 | 0.5368 | 0.4149 | 0.0019 | 0.5388 | 0.5369 |
| chr1H | 15500000 | 0.5364 | 0.4152 | 0.0017 | 0.5384 | 0.5367 |
| chr1H | 15600000 | 0.537 | 0.4156 | 0.0033 | 0.5428 | 0.5394 |
| chr1H | 15800000 | 0.5393 | 0.4171 | 0.0052 | 0.5477 | 0.5426 |
| chr1H | 15900000 | 0.54 | 0.4179 | 0.0079 | 0.564 | 0.5561 |
| chr1H | 16000000 | 0.5453 | 0.4219 | 0.0136 | 0.5875 | 0.5739 |
| chr1H | 16100000 | 0.5472 | 0.4234 | 0.0173 | 0.5968 | 0.5795 |
| chr1H | 16200000 | 0.5474 | 0.4235 | 0.0177 | 0.5974 | 0.5797 |
| chr1H | 16300000 | 0.5486 | 0.4243 | 0.0193 | 0.6037 | 0.5844 |
| chr1H | 16400000 | 0.5485 | 0.4239 | 0.0233 | 0.6069 | 0.5836 |
| chr1H | 16500000 | 0.5527 | 0.4263 | 0.0302 | 0.6149 | 0.5847 |
| chr1H | 16600000 | 0.5524 | 0.4263 | 0.0289 | 0.6122 | 0.5833 |
| chr1H | 16700000 | 0.5524 | 0.4263 | 0.0289 | 0.6122 | 0.5833 |
| chr1H | 16800000 | 0.5507 | 0.4251 | 0.0308 | 0.6077 | 0.5769 |
| chr1H | 16900000 | 0.5508 | 0.4252 | 0.0309 | 0.6066 | 0.5756 |
| chr1H | 17000000 | 0.5523 | 0.4267 | 0.0316 | 0.6043 | 0.5727 |
| chr1H | 17100000 | 0.5527 | 0.427 | 0.0317 | 0.6053 | 0.5736 |
| chr1H | 17200000 | 0.5527 | 0.4271 | 0.032 | 0.6054 | 0.5734 |
| chr1H | 17300000 | 0.5527 | 0.4271 | 0.032 | 0.6054 | 0.5734 |
| chr1H | 17400000 | 0.553 | 0.4273 | 0.0291 | 0.607 | 0.5779 |
| chr1H | 17500000 | 0.553 | 0.4273 | 0.0291 | 0.607 | 0.5779 |
| chr1H | 17600000 | 0.5531 | 0.4274 | 0.0277 | 0.6037 | 0.5761 |
| chr1H | 17700000 | 0.554 | 0.4283 | 0.0289 | 0.6141 | 0.5852 |
| chr1H | 17800000 | 0.5534 | 0.4281 | 0.0315 | 0.6238 | 0.5923 |
| chr1H | 17900000 | 0.5535 | 0.4282 | 0.0321 | 0.6327 | 0.6006 |
| chr1H | 18000000 | 0.5533 | 0.4285 | 0.0285 | 0.6246 | 0.5961 |
| chr1H | 18100000 | 0.5536 | 0.4282 | 0.0286 | 0.6246 | 0.596 |
| chr1H | 18200000 | 0.5534 | 0.4284 | 0.0282 | 0.6253 | 0.5971 |
| chr1H | 18300000 | 0.5509 | 0.4274 | 0.0253 | 0.6023 | 0.577 |
| chr1H | 18400000 | 0.5538 | 0.4315 | 0.019 | 0.5844 | 0.5654 |
| chr1H | 18500000 | 0.5335 | 0.4146 | 0.0465 | 0.632 | 0.5854 |
| chr1H | 18600000 | 0.5245 | 0.4069 | 0.0546 | 0.6471 | 0.5925 |
| chr1H | 18700000 | 0.5396 | 0.4187 | 0.036 | 0.6093 | 0.5733 |
| chr1H | 18800000 | 0.5413 | 0.42 | 0.0337 | 0.6053 | 0.5716 |
| chr1H | 18900000 | 0.54 | 0.4188 | 0.0308 | 0.5893 | 0.5585 |
| chr1H | 19000000 | 0.5368 | 0.4161 | 0.0291 | 0.5818 | 0.5527 |
| chr1H | 20900000 | 0.5289 | 0.4099 | 0.0679 | 0.597 | 0.5291 |
| chr1H | 21000000 | 0.5293 | 0.4101 | 0.0707 | 0.6005 | 0.5298 |
| chr1H | 21100000 | 0.529 | 0.4097 | 0.0767 | 0.6126 | 0.536 |
| chr1H | 21200000 | 0.5292 | 0.4094 | 0.086 | 0.6161 | 0.5301 |
| chr2H | 106900000 | 0.675 | 0.5 | 0.0 | 0.7 | 0.7 |
| chr2H | 107000000 | 0.675 | 0.5 | 0.0 | 0.7 | 0.7 |
| chr2H | 107100000 | 0.675 | 0.5 | 0.0 | 0.7 | 0.7 |
| chr2H | 107200000 | 0.675 | 0.5 | 0.0 | 0.7 | 0.7 |
| chr2H | 107300000 | 0.675 | 0.5 | 0.0 | 0.7 | 0.7 |
| chr2H | 107400000 | 0.675 | 0.5 | 0.0 | 0.7 | 0.7 |
| chr2H | 107500000 | 0.675 | 0.5 | 0.0 | 0.7 | 0.7 |
| chr2H | 107600000 | 0.675 | 0.5 | 0.0 | 0.7 | 0.7 |
| chr2H | 107700000 | 0.675 | 0.5 | 0.0 | 0.7 | 0.7 |
| chr2H | 107800000 | 0.675 | 0.5 | 0.0 | 0.7 | 0.7 |
| chr2H | 107900000 | 0.675 | 0.5 | 0.0 | 0.7 | 0.7 |
| chr2H | 108000000 | 0.675 | 0.5 | 0.0 | 0.7 | 0.7 |
| chr2H | 108100000 | 0.675 | 0.5 | 0.0 | 0.7 | 0.7 |
| chr2H | 108200000 | 0.675 | 0.5 | 0.0 | 0.7 | 0.7 |
| chr2H | 108300000 | 0.675 | 0.5 | 0.0 | 0.7 | 0.7 |
| chr2H | 108400000 | 0.675 | 0.5 | 0.0 | 0.7 | 0.7 |
| chr2H | 108500000 | 0.675 | 0.5 | 0.0 | 0.7 | 0.7 |
| chr2H | 108600000 | 0.675 | 0.5 | 0.0 | 0.7 | 0.7 |
| chr2H | 330900000 | 0.6023 | 0.4773 | 0.0 | 0.6364 | 0.6364 |
| chr2H | 331000000 | 0.6023 | 0.4773 | 0.0 | 0.6364 | 0.6364 |
| chr2H | 331100000 | 0.6023 | 0.4773 | 0.0 | 0.6364 | 0.6364 |
| chr2H | 331200000 | 0.6023 | 0.4773 | 0.0 | 0.6364 | 0.6364 |
| chr2H | 331300000 | 0.6023 | 0.4773 | 0.0 | 0.6364 | 0.6364 |
| chr2H | 647400000 | 0.5682 | 0.4773 | 0.1667 | 0.8182 | 0.6515 |
| chr2H | 647500000 | 0.5682 | 0.4773 | 0.1667 | 0.8182 | 0.6515 |
| chr2H | 647600000 | 0.5682 | 0.4773 | 0.1667 | 0.8182 | 0.6515 |
| chr2H | 647700000 | 0.5682 | 0.4773 | 0.1667 | 0.8182 | 0.6515 |
| chr2H | 647800000 | 0.5682 | 0.4773 | 0.1667 | 0.8182 | 0.6515 |
| chr2H | 647900000 | 0.5682 | 0.4773 | 0.1667 | 0.8182 | 0.6515 |
| chr2H | 648000000 | 0.5682 | 0.4773 | 0.1667 | 0.8182 | 0.6515 |
| chr2H | 648100000 | 0.5682 | 0.4773 | 0.1667 | 0.8182 | 0.6515 |
| chr2H | 648200000 | 0.5682 | 0.4773 | 0.1667 | 0.8182 | 0.6515 |
| chr2H | 648300000 | 0.5682 | 0.4773 | 0.1667 | 0.8182 | 0.6515 |
| chr2H | 648400000 | 0.5682 | 0.4773 | 0.1667 | 0.8182 | 0.6515 |
| chrUn | 12800000 | 0.5274 | 0.408 | 0.6214 | 0.0833 | -0.5381 |
| chrUn | 12900000 | 0.5274 | 0.408 | 0.6214 | 0.0833 | -0.5381 |
| chrUn | 13000000 | 0.5274 | 0.408 | 0.6214 | 0.0833 | -0.5381 |
| chrUn | 13100000 | 0.5274 | 0.408 | 0.6214 | 0.0833 | -0.5381 |
| chrUn | 13200000 | 0.5274 | 0.408 | 0.6214 | 0.0833 | -0.5381 |
| chrUn | 13300000 | 0.5274 | 0.408 | 0.6214 | 0.0833 | -0.5381 |
| chrUn | 13400000 | 0.5274 | 0.408 | 0.6214 | 0.0833 | -0.5381 |

**Supplementary Table 4** Statistically significant sliding window regions in Jeonju182-HvPMS20 vs. R20 (*p < 0.01*). POSI, central position of window (window size = 2 Mb, step = 100 kb). Mean_p99, p95, the mean absolute value of the single nucleotide polymorphism (SNP) index at the 99%, 95% confidence interval upper/lower limits (or the mean absolute value of the SNP index corresponding to *p = 0.01, 0.05*) was used as a threshold. SNPs with an absolute mean SNP index exceeding this value were considered significant at a significance level of 0.01, 0.05.

| **CHROM** | **POSI** | **mean_p99** | **mean_p95** | **mean_SNP**  **index1** | **mean_SNP**  **index2** | **mean_SNP**  **index** |
| --- | --- | --- | --- | --- | --- | --- |
| chr1H | 6100000 | 0.5327 | 0.4126 | 0.9343 | 0.4009 | -0.5334 |
| chr1H | 6200000 | 0.5326 | 0.4124 | 0.9337 | 0.397 | -0.5367 |
| chr1H | 6300000 | 0.5326 | 0.4124 | 0.9337 | 0.397 | -0.5367 |
| chr1H | 6400000 | 0.5331 | 0.4129 | 0.9407 | 0.4068 | -0.5338 |
| chr1H | 6500000 | 0.5333 | 0.4131 | 0.9417 | 0.4061 | -0.5356 |
| chr1H | 6900000 | 0.5152 | 0.3987 | 0.9322 | 0.3998 | -0.5324 |
| chr1H | 7000000 | 0.5148 | 0.3987 | 0.9286 | 0.3858 | -0.5427 |
| chr1H | 7100000 | 0.5152 | 0.3988 | 0.9304 | 0.3786 | -0.5518 |
| chr1H | 7200000 | 0.5139 | 0.3978 | 0.9308 | 0.3793 | -0.5515 |
| chr1H | 7300000 | 0.5168 | 0.4004 | 0.9248 | 0.3718 | -0.553 |
| chr1H | 7400000 | 0.5184 | 0.4018 | 0.9284 | 0.3707 | -0.5577 |
| chr1H | 7500000 | 0.5193 | 0.4026 | 0.931 | 0.3667 | -0.5643 |
| chr1H | 7600000 | 0.5192 | 0.4026 | 0.9341 | 0.3632 | -0.5709 |
| chr1H | 7700000 | 0.5198 | 0.403 | 0.9353 | 0.3598 | -0.5755 |
| chr1H | 7800000 | 0.5204 | 0.403 | 0.9363 | 0.3455 | -0.5908 |
| chr1H | 7900000 | 0.5203 | 0.4032 | 0.9384 | 0.3439 | -0.5945 |
| chr1H | 8000000 | 0.521 | 0.4037 | 0.9387 | 0.3354 | -0.6033 |
| chr1H | 8100000 | 0.5214 | 0.404 | 0.9395 | 0.3335 | -0.606 |
| chr1H | 8200000 | 0.5218 | 0.4042 | 0.9399 | 0.334 | -0.6058 |
| chr1H | 8300000 | 0.522 | 0.4045 | 0.9406 | 0.3347 | -0.6059 |
| chr1H | 8400000 | 0.5218 | 0.4043 | 0.9459 | 0.3363 | -0.6096 |
| chr1H | 8500000 | 0.5215 | 0.4041 | 0.9508 | 0.3447 | -0.6062 |
| chr1H | 8600000 | 0.525 | 0.4068 | 0.959 | 0.3404 | -0.6186 |
| chr1H | 8700000 | 0.5343 | 0.4145 | 0.9742 | 0.3258 | -0.6483 |
| chr1H | 8800000 | 0.5335 | 0.4139 | 0.9769 | 0.3247 | -0.6522 |
| chr1H | 8900000 | 0.5352 | 0.4153 | 0.9797 | 0.3255 | -0.6542 |
| chr1H | 9000000 | 0.5343 | 0.4144 | 0.9801 | 0.3301 | -0.65 |
| chr1H | 9100000 | 0.5324 | 0.4132 | 0.9805 | 0.3415 | -0.639 |
| chr1H | 9200000 | 0.5316 | 0.4123 | 0.9823 | 0.3534 | -0.629 |
| chr1H | 9300000 | 0.5241 | 0.4062 | 0.9913 | 0.3751 | -0.6162 |
| chr1H | 9400000 | 0.5225 | 0.4047 | 0.9913 | 0.3781 | -0.6131 |
| chr1H | 9500000 | 0.5216 | 0.404 | 0.9897 | 0.3814 | -0.6083 |
| chr1H | 9600000 | 0.5233 | 0.4052 | 0.9888 | 0.3863 | -0.6025 |
| chr1H | 9700000 | 0.5231 | 0.4051 | 0.9887 | 0.3881 | -0.6006 |
| chr1H | 9800000 | 0.5208 | 0.4036 | 0.98 | 0.3974 | -0.5826 |
| chr1H | 9900000 | 0.5203 | 0.4032 | 0.981 | 0.4002 | -0.5808 |
| chr1H | 10000000 | 0.5188 | 0.4019 | 0.9837 | 0.4117 | -0.572 |
| chr1H | 10100000 | 0.5181 | 0.4013 | 0.9842 | 0.4128 | -0.5714 |
| chr1H | 10200000 | 0.518 | 0.4013 | 0.9848 | 0.4115 | -0.5733 |
| chr1H | 10300000 | 0.5183 | 0.4015 | 0.985 | 0.4121 | -0.5729 |
| chr1H | 10400000 | 0.5208 | 0.4036 | 0.9842 | 0.4167 | -0.5675 |
| chr1H | 10500000 | 0.5226 | 0.4048 | 0.9814 | 0.4077 | -0.5737 |
| chr1H | 10600000 | 0.5234 | 0.4055 | 0.9826 | 0.4006 | -0.582 |
| chr1H | 10700000 | 0.5225 | 0.4046 | 0.9696 | 0.3911 | -0.5785 |
| chr1H | 10800000 | 0.5229 | 0.4047 | 0.9702 | 0.3854 | -0.5849 |
| chr1H | 10900000 | 0.5251 | 0.4063 | 0.9713 | 0.3709 | -0.6004 |
| chr1H | 11000000 | 0.5259 | 0.4071 | 0.9704 | 0.3662 | -0.6041 |
| chr1H | 11100000 | 0.528 | 0.4085 | 0.9704 | 0.3562 | -0.6142 |
| chr1H | 11200000 | 0.5284 | 0.409 | 0.9675 | 0.3411 | -0.6264 |
| chr1H | 11300000 | 0.5373 | 0.4159 | 0.9627 | 0.3126 | -0.65 |
| chr1H | 11400000 | 0.5389 | 0.4172 | 0.9608 | 0.3065 | -0.6544 |
| chr1H | 11500000 | 0.5397 | 0.4177 | 0.9619 | 0.305 | -0.6569 |
| chr1H | 11600000 | 0.5392 | 0.4173 | 0.9593 | 0.2933 | -0.6659 |
| chr1H | 11700000 | 0.5384 | 0.4165 | 0.9597 | 0.2942 | -0.6654 |
| chr1H | 11800000 | 0.5403 | 0.4182 | 0.9731 | 0.2943 | -0.6788 |
| chr1H | 11900000 | 0.5407 | 0.4184 | 0.9716 | 0.2895 | -0.682 |
| chr1H | 12000000 | 0.5407 | 0.4184 | 0.9716 | 0.2895 | -0.682 |
| chr1H | 12100000 | 0.543 | 0.4203 | 0.9691 | 0.2834 | -0.6857 |
| chr1H | 12200000 | 0.5439 | 0.4208 | 0.9692 | 0.2835 | -0.6857 |
| chr1H | 12300000 | 0.5452 | 0.4214 | 0.9697 | 0.2715 | -0.6982 |
| chr1H | 12400000 | 0.5438 | 0.4201 | 0.9707 | 0.2629 | -0.7078 |
| chr1H | 12500000 | 0.5444 | 0.4206 | 0.9713 | 0.2623 | -0.7089 |
| chr1H | 12600000 | 0.5443 | 0.4203 | 0.9705 | 0.2673 | -0.7031 |
| chr1H | 12700000 | 0.5474 | 0.4224 | 0.9925 | 0.2741 | -0.7184 |
| chr1H | 12800000 | 0.5471 | 0.4223 | 0.9918 | 0.2767 | -0.7151 |
| chr1H | 12900000 | 0.5479 | 0.4229 | 0.9965 | 0.2966 | -0.7 |
| chr1H | 13000000 | 0.5491 | 0.4233 | 0.995 | 0.3082 | -0.6868 |
| chr1H | 13100000 | 0.5516 | 0.4248 | 0.9942 | 0.3064 | -0.6878 |
| chr1H | 13200000 | 0.5464 | 0.421 | 0.9952 | 0.3364 | -0.6588 |
| chr1H | 13300000 | 0.5464 | 0.421 | 0.9952 | 0.3364 | -0.6588 |
| chr1H | 13400000 | 0.5464 | 0.421 | 0.9952 | 0.3364 | -0.6588 |
| chr1H | 13500000 | 0.5429 | 0.4184 | 0.9959 | 0.362 | -0.6339 |
| chr1H | 13600000 | 0.5429 | 0.4183 | 0.9962 | 0.3668 | -0.6294 |
| chr1H | 13700000 | 0.5421 | 0.4181 | 0.9946 | 0.3606 | -0.634 |
| chr1H | 13800000 | 0.5399 | 0.4169 | 0.9955 | 0.3817 | -0.6138 |
| chr1H | 13900000 | 0.5393 | 0.4162 | 0.9962 | 0.4003 | -0.5959 |
| chr1H | 14000000 | 0.5366 | 0.4143 | 0.9968 | 0.4094 | -0.5873 |
| chr1H | 14100000 | 0.5363 | 0.4146 | 0.9971 | 0.4084 | -0.5887 |
| chr1H | 14200000 | 0.5355 | 0.4141 | 0.9971 | 0.4108 | -0.5863 |
| chr1H | 14300000 | 0.5348 | 0.4136 | 0.997 | 0.4169 | -0.58 |
| chr1H | 14400000 | 0.534 | 0.4133 | 0.9977 | 0.4261 | -0.5716 |
| chr1H | 14500000 | 0.5328 | 0.4125 | 0.9981 | 0.4208 | -0.5773 |
| chr1H | 14600000 | 0.5332 | 0.413 | 0.9981 | 0.42 | -0.5781 |
| chr1H | 14700000 | 0.5332 | 0.413 | 0.9981 | 0.42 | -0.5781 |
| chr1H | 14800000 | 0.5344 | 0.4138 | 0.9981 | 0.4175 | -0.5806 |
| chr1H | 14900000 | 0.5341 | 0.4136 | 0.9981 | 0.4192 | -0.5789 |
| chr1H | 15000000 | 0.5324 | 0.4126 | 0.9988 | 0.4197 | -0.5791 |
| chr1H | 15100000 | 0.5316 | 0.4122 | 0.9988 | 0.4211 | -0.5777 |
| chr1H | 15200000 | 0.533 | 0.4132 | 0.9987 | 0.4176 | -0.5812 |
| chr1H | 15300000 | 0.533 | 0.4132 | 0.9987 | 0.4176 | -0.5812 |
| chr1H | 15400000 | 0.533 | 0.4132 | 0.9987 | 0.4176 | -0.5812 |
| chr1H | 15500000 | 0.5338 | 0.414 | 0.9986 | 0.4103 | -0.5884 |
| chr1H | 15600000 | 0.5338 | 0.4142 | 0.9964 | 0.4064 | -0.59 |
| chr1H | 15700000 | 0.5349 | 0.4151 | 0.9951 | 0.4165 | -0.5785 |
| chr1H | 15800000 | 0.5366 | 0.4164 | 0.9923 | 0.4047 | -0.5876 |
| chr1H | 15900000 | 0.5388 | 0.4189 | 0.9868 | 0.3842 | -0.6026 |
| chr1H | 16000000 | 0.547 | 0.4256 | 0.9754 | 0.3533 | -0.622 |
| chr1H | 16100000 | 0.5503 | 0.4279 | 0.9704 | 0.3435 | -0.627 |
| chr1H | 16200000 | 0.5512 | 0.4285 | 0.9661 | 0.3404 | -0.6258 |
| chr1H | 16300000 | 0.5526 | 0.4294 | 0.9589 | 0.3359 | -0.623 |
| chr1H | 16400000 | 0.553 | 0.4293 | 0.9544 | 0.3353 | -0.6191 |
| chr1H | 16500000 | 0.5571 | 0.4327 | 0.9461 | 0.3327 | -0.6134 |
| chr1H | 16600000 | 0.5573 | 0.4326 | 0.9477 | 0.3361 | -0.6116 |
| chr1H | 16700000 | 0.5573 | 0.4326 | 0.9477 | 0.3361 | -0.6116 |
| chr1H | 16800000 | 0.5561 | 0.4318 | 0.9454 | 0.3374 | -0.608 |
| chr1H | 16900000 | 0.5562 | 0.4319 | 0.9454 | 0.3387 | -0.6067 |
| chr1H | 17000000 | 0.5578 | 0.4331 | 0.945 | 0.3408 | -0.6042 |
| chr1H | 17100000 | 0.5582 | 0.4333 | 0.9449 | 0.3401 | -0.6048 |
| chr1H | 17200000 | 0.5583 | 0.4334 | 0.9446 | 0.3399 | -0.6047 |
| chr1H | 17300000 | 0.5583 | 0.4334 | 0.9446 | 0.3399 | -0.6047 |
| chr1H | 17400000 | 0.5583 | 0.4334 | 0.9446 | 0.3399 | -0.6047 |
| chr1H | 17500000 | 0.5583 | 0.4334 | 0.9446 | 0.3399 | -0.6047 |
| chr1H | 17600000 | 0.5598 | 0.4345 | 0.9437 | 0.339 | -0.6047 |
| chr1H | 17700000 | 0.5609 | 0.4353 | 0.9412 | 0.3258 | -0.6154 |
| chr1H | 17800000 | 0.5615 | 0.4358 | 0.9381 | 0.3212 | -0.617 |
| chr1H | 17900000 | 0.5632 | 0.4367 | 0.9356 | 0.3124 | -0.6232 |
| chr1H | 18000000 | 0.5611 | 0.4348 | 0.9372 | 0.3226 | -0.6145 |
| chr1H | 18100000 | 0.56 | 0.4338 | 0.9352 | 0.3229 | -0.6122 |
| chr1H | 18200000 | 0.5583 | 0.4325 | 0.9442 | 0.3303 | -0.614 |
| chr1H | 18300000 | 0.5558 | 0.431 | 0.9697 | 0.3468 | -0.6229 |
| chr1H | 18400000 | 0.5589 | 0.4359 | 0.9788 | 0.3636 | -0.6152 |
| chr1H | 18500000 | 0.5312 | 0.4123 | 0.8972 | 0.3115 | -0.5856 |
| chr1H | 18600000 | 0.5215 | 0.4049 | 0.8839 | 0.3048 | -0.5792 |
| chr1H | 18700000 | 0.5377 | 0.4176 | 0.9248 | 0.3427 | -0.5821 |
| chr1H | 18800000 | 0.5394 | 0.4193 | 0.9285 | 0.3457 | -0.5828 |
| chr1H | 18900000 | 0.5373 | 0.4174 | 0.9338 | 0.3644 | -0.5694 |
| chr1H | 19000000 | 0.5345 | 0.4148 | 0.9391 | 0.3753 | -0.5638 |
| chr1H | 19100000 | 0.5343 | 0.4149 | 0.9449 | 0.4009 | -0.544 |
| chr1H | 19300000 | 0.5328 | 0.4136 | 0.9525 | 0.4171 | -0.5354 |
| chr1H | 19400000 | 0.533 | 0.4137 | 0.9529 | 0.4168 | -0.5361 |
| chr1H | 19500000 | 0.5327 | 0.4135 | 0.9532 | 0.4158 | -0.5374 |
| chr1H | 19600000 | 0.5323 | 0.4132 | 0.9537 | 0.4143 | -0.5394 |
| chr1H | 19700000 | 0.5322 | 0.4131 | 0.9537 | 0.4146 | -0.5391 |
| chr1H | 19800000 | 0.5322 | 0.4131 | 0.9535 | 0.415 | -0.5386 |
| chr1H | 19900000 | 0.532 | 0.413 | 0.952 | 0.4143 | -0.5377 |
| chr1H | 20000000 | 0.5349 | 0.4151 | 0.9363 | 0.3926 | -0.5436 |
| chr1H | 20100000 | 0.5345 | 0.4147 | 0.9359 | 0.3949 | -0.541 |
| chr1H | 20200000 | 0.5336 | 0.4138 | 0.9355 | 0.3955 | -0.54 |
| chr1H | 20300000 | 0.533 | 0.4133 | 0.9364 | 0.3964 | -0.54 |
| chr1H | 20400000 | 0.5326 | 0.4128 | 0.9339 | 0.3946 | -0.5393 |
| chr1H | 20600000 | 0.5394 | 0.4174 | 0.9266 | 0.3864 | -0.5402 |
| chr2H | 331200000 | 0.6364 | 0.5114 | 1.0 | 0.3636 | -0.6364 |
| chr2H | 331300000 | 0.6364 | 0.5114 | 1.0 | 0.3636 | -0.6364 |
| chr2H | 646900000 | 0.5758 | 0.4697 | 0.8333 | 0.1818 | -0.6515 |
| chr2H | 647000000 | 0.5758 | 0.4697 | 0.8333 | 0.1818 | -0.6515 |
| chr2H | 647100000 | 0.5758 | 0.4697 | 0.8333 | 0.1818 | -0.6515 |
| chr2H | 647200000 | 0.5758 | 0.4697 | 0.8333 | 0.1818 | -0.6515 |
| chr2H | 647300000 | 0.5758 | 0.4697 | 0.8333 | 0.1818 | -0.6515 |
| chr2H | 647400000 | 0.5758 | 0.4697 | 0.8333 | 0.1818 | -0.6515 |
| chr2H | 647500000 | 0.5758 | 0.4697 | 0.8333 | 0.1818 | -0.6515 |
| chr2H | 647600000 | 0.5758 | 0.4697 | 0.8333 | 0.1818 | -0.6515 |
| chr2H | 647700000 | 0.5758 | 0.4697 | 0.8333 | 0.1818 | -0.6515 |
| chr2H | 647800000 | 0.5758 | 0.4697 | 0.8333 | 0.1818 | -0.6515 |
| chr2H | 647900000 | 0.5758 | 0.4697 | 0.8333 | 0.1818 | -0.6515 |
| chr2H | 648000000 | 0.5758 | 0.4697 | 0.8333 | 0.1818 | -0.6515 |
| chr2H | 648100000 | 0.5758 | 0.4697 | 0.8333 | 0.1818 | -0.6515 |
| chr2H | 648200000 | 0.5758 | 0.4697 | 0.8333 | 0.1818 | -0.6515 |
| chr2H | 648300000 | 0.5758 | 0.4697 | 0.8333 | 0.1818 | -0.6515 |
| chr2H | 648400000 | 0.5758 | 0.4697 | 0.8333 | 0.1818 | -0.6515 |
| chr2H | 648500000 | 0.5758 | 0.4697 | 0.8333 | 0.1818 | -0.6515 |
| chr2H | 648600000 | 0.5758 | 0.4697 | 0.8333 | 0.1818 | -0.6515 |
| chr2H | 648700000 | 0.5758 | 0.4697 | 0.8333 | 0.1818 | -0.6515 |
| chr2H | 648800000 | 0.5758 | 0.4697 | 0.8333 | 0.1818 | -0.6515 |
| chrUn | 12800000 | 0.538 | 0.4139 | 0.3786 | 0.9167 | 0.5381 |
| chrUn | 12900000 | 0.538 | 0.4139 | 0.3786 | 0.9167 | 0.5381 |
| chrUn | 13000000 | 0.538 | 0.4139 | 0.3786 | 0.9167 | 0.5381 |
| chrUn | 13100000 | 0.538 | 0.4139 | 0.3786 | 0.9167 | 0.5381 |
| chrUn | 13200000 | 0.538 | 0.4139 | 0.3786 | 0.9167 | 0.5381 |
| chrUn | 13300000 | 0.538 | 0.4139 | 0.3786 | 0.9167 | 0.5381 |
| chrUn | 13400000 | 0.538 | 0.4139 | 0.3786 | 0.9167 | 0.5381 |

**Supplementary Table 5** Candidate quantitative trait loci (QTL) regions and common variants identified in two QTL-seq analyses.

| QTL-seq analysis | Chr | Sliding window  (*p<0.01*) | bp | Variants  (SNP/InDel) |
| --- | --- | --- | --- | --- |
| Hopum-S20 vs R20 | 1H | 1,5000,000-5,500,000 | 4,000,000 | 569 |
|  | 1H | 6,800,000-21,200,000 | 14,400,000 | 2,674 |
|  | 2H | 106,900,000-108,600,000 | 1,700,000 | 3 |
|  | 2H | 330,900,000-331,300,000 | 400,000 | 1 |
|  | **2H** | **647,400,000-648,400,000** | **1,000,000** | **1** |
| Jeonju182-S20 vs R20 | 1H | 6,100,000-6,500,000 | 400,000 | 27 |
|  | **1H** | **6,900,000-20,600,000** | **13,700,000** | **2,690** |
|  | **2H** | **331,200,000-331,300,000** | **100,000** | **-** |
|  | 2H | 646,900,000-648,800,000 | 1,900,000 | 1 |

**Supplementary Table 6** Variant information identified through filtering within candidate quantitative trait loci regions.

| Position(bp) | Chr | Variants type | Variants |
| --- | --- | --- | --- |
| 6,940,595-18,008,713 | 1H | SNP/InDel | 1,640/322 |
| 19,363,700-20,551,018 |  | SNP/InDel | 156/12 |

**Supplementary Table 7** List of 134 cleaved amplified polymorphic sequence markers designed based on variant analysis within candidate quantitative trait loci (QTL) regions. The markers shaded in gray represent non-synonymous single nucleotide polymorphism (SNP) variants.

| Marker | Chr | Position | Ref | Alt | Sequence | Product size  (bp) | Restriction enzyme | Variants type  (effect) |
| --- | --- | --- | --- | --- | --- | --- | --- | --- |
| PMC_1 | 1H | 7649737 | T | C | (F) TCACAACTCTGGATGAGATCAG  (R) TTACACCAAGAGATTGATTCCC | 541 | HpyCH4IV | SNP(INTRON) |
| PMC_2 | 1H | 7764837 | G | A | (F) CTGTATGCCCAGAATATGGTTT  (R) TCTTCTCTCTTCTCACCACCAT | 496 | HpyCH4V | SNP(INTRON) |
| PMC_3 | 1H | 7767944 | A | T | (F) ATAACAATATCTCATCGCCCAG  (R) GTATCCAAAGACACCATCGACT | 599 | AseI | SNP(INTRON) |
| PMC_4 | 1H | 7842041 | A | G | (F) GTTGAATCTGGAATTCTGAAGC  (R) CAAGAAGAGAACCTCCACTCAC | 327 | BstUI | SNP  (non-synonymous) |
| PMC_5 | 1H | 7844268 | A | G | (F) CGAACAATCTAGCCTTATACCG  (R) GCCTCATATGTGAGTAAGGAGC | 597 | CviAII | SNP(INTRON) |
| PMC_6 | 1H | 7844300 | G | C | (F) CGAACAATCTAGCCTTATACCG  (R) GCCTCATATGTGAGTAAGGAGC | 597 | MboI | SNP(INTRON) |
| PMC_7 | 1H | 7844734 | T | C | (F) TTACAAGTGCATGGAAGTTCTG  (R) CGATGACCCATAACTGTAGGAT | 317 | AluI | SNP(INTRON) |
| PMC_8 | 1H | 7845395 | G | A | (F) GCACGTTATGTACCAACCCTAT  (R) ACTCTTGACAGAGGCAGTGAAT | 439 | HpyCH4V | SNP(INTRON) |
| PMC_9 | 1H | 7846629 | A | G | (F) TAAGGAGACAAGTAGGAGCAGC  (R) GAGATCCATGGTTTCTGATTGT | 518 | DdeI | SNP(INTRON) |
| PMC_10 | 1H | 7847724 | T | A | (F) TGGATTGCATATGAAGTGAGAA  (R) CAAGATCAACAGGTCCTCTCTC | 588 | DdeI | SNP  (synonymous) |
| PMC_11 | 1H | 7852288 | G | A | (F) ACTTGCGCTAACCTTGTATTGT  (R) TTCTCAGGTGGCAGGTTAGTAT | 600 | TaqI | SNP  (synonymous) |
| PMC_12 | 1H | 7878422 | C | G | (F) ATCCACTTTCTCTGCCTTACAA\  (R) CATGTTCCACACTCATTTATGG | 663 | HinfI | SNP(INTRON) |
| PMC_13 | 1H | 7879077 | T | C | (F) TCTGCTTTCTAGTAAAGTCGCC  (R) AGCTCAATAGAAGAATCACCCA | 353 | Bsp1286I | SNP(INTRON) |
| PMC_14 | 1H | 7879663 | G | A | (F) TTCTTCCACAGTCTTCTAGGGA  (R) AGAATCCAACCTAACTGAAGCA | 650 | HpyCH4V | SNP  (synonymous) |
| PMC_15 | 1H | 8211216 | TAA | TA | (F) AAGCATAAATTTGATTAGCGGA  (R) GAGTTCCAGGAAACTTCCTCTT | 624 | AvaII | InDel  (INTRON) |
| PMC_16 | 1H | 8214697 | G | A | (F) CACTTCCTTCCACGGTAGATAA  (R) TGTTTATATTCGAACATGGCAA | 599 | TaqI | SNP(INTRON) |
| PMC_17 | 1H | 8215796 | G | T | (F) AGGTCGTACGTCCACATAATTC  (R) ATCTGGTAACGGATGAACTCC | 646 | HpaII | SNP(INTRON) |
| PMC_18 | 1H | 8215983 | C | G | (F) AGGTCGTACGTCCACATAATTC  (R) ATCTGGTAACGGATGAACTCC | 646 | CviAII | SNP(INTRON) |
| PMC_19 | 1H | 8354842 | C | T | (F) ATGTAACAAATCTGCAATTCCC  (R) GCACAAACCATACCATACCTTT | 553 | HpyCH4IV | SNP(INTRON) |
| PMC_20 | 1H | 8356477 | G | T | (F) TCTAGTCATCGCGACATATCAG  (R) GCTTCGAAGGTCTGCTCTATTA | 387 | EcoRV | SNP(INTRON) |
| PMC_21 | 1H | 8531382 | G | C | (F) GTACCTGTAATCCAGCACCAAT  (R) GAGCAGTGAAGAAGGTAATGCT | 547 | CviAII | SNP(INTRON) |
| PMC_22 | 1H | 8532858 | T | C | (F) CCGTTACCATAACAATCCTCAT  (R) TTCTGGTATGGTCATCAGTTCA | 664 | AluI | SNP(UTR_5) |
| PMC_23 | 1H | 8870292 | C | T | (F) CCACTTGAGGAGAACAACTTTC  (R) CATCATATTCAATCCAATGCTG | 520 | NlaⅢ | SNP  (non-synonymous) |
| PMC_24 | 1H | 8875545 | A | G | (F) CGATGATATTCGCTCACAGATA  (R) ATACATGGCAATAGGAATCGAC | 649 | CviAII | SNP(INTRON) |
| PMC_25 | 1H | 8876314 | G | A | (F) ATCCTCAATCTTAGCCAGTGAA  (R) TTTAATATGGGACGTCTGGTTC | 472 | CviQI | SNP(INTRON) |
| PMC_26 | 1H | 8877543 | G | A | (F) GTCTCTATGGATTATGGGTGGA  (R) TAGAAGTTGCTTGGAACCAGAT | 611 | DdeI | SNP(INTRON) |
| PMC_27 | 1H | 8936182 | C | T | (F) CTACTGTACCGAGCACATTGAA  (R) GTATCACGATCAACATCACCAG | 564 | BsmI | SNP(INTRON) |
| PMC_28 | 1H | 8938251 | A | G | (F) ACGAGTTCAAGACCACCTACC  (R) TTTCGGAATATTGAAGCTGTTT | 622 | HpaⅡ | SNP  (non-synonymous) |
| PMC_29 | 1H | 8951116 | G | A | (F) GAAGGTCCTCTTATGCTGTACG  (R) AACACCATCCATAAAGTCGTTC | 543 | CviQⅠ | SNP  (non-synonymous) |
| PMC_30 | 1H | 8951914 | G | A | (F) GAACGACTTTATGGATGGTGTT  (R) TGCAGTGTCGTTTCTTCTCTTA | 672 | BsaAⅠ | SNP  (non-synonymous) |
| PMC_31 | 1H | 9312497 | G | C | (F) AGATCTCGTTTCGTCTTGGTT  (R) ACTGCTATGAACTTGGACTGCT | 669 | NaeⅠ | SNP  (non-synonymous) |
| PMC_32 | 1H | 9313151 | G | A | (F) AGCAGTCCAAGTTCATAGCAGT  (R) AGTTGTAACATGGAACCAGGAC | 594 | BfaI | SNP(INTRON) |
| PMC_33 | 1H | 9313502 | T | C | (F) CAAATCAACGTGATGTGGATAG  (R) ATGACAGGTTCATCCCAGATAG | 393 | AluI | SNP  (synonymous) |
| PMC_34 | 1H | 9313685 | C | T | (F) CAAATCAACGTGATGTGGATAG  (R) ATGACAGGTTCATCCCAGATAG | 393 | HinP1I | SNP  (synonymous) |
| PMC_35 | 1H | 9330617 | T | C | (F) CCGTTTGTCTGTCCAAATTAGT  (R) CTTCAAGTAGGACCTCACGTTC | 513 | CviAII | SNP(INTRON) |
| PMC_36 | 1H | 9330782 | G | C | (F) CTCACGAGTGGCTATGACATTA  (R) CTTCAAGTAGGACCTCACGTTC | 340 | MboI | SNP  (non-synonymous) |
| PMC_37 | 1H | 9331963 | A | G | (F) TCTAAGAGGTGAGCAACAGGAT  (R) TGGTTGAACTTCAGTACAGTCG | 432 | HinfI | SNP(INTRON) |
| PMC_38 | 1H | 9334843 | TA | TAA | (F) CATGTCACAGTTTGAAGGATTG  (R) TCCATCTTGGTACCATTAAAGC | 462 | AflII | InDel  (INTRON) |
| PMC_39 | 1H | 9336648 | G | C | (F) ATAATCGTAGCCTTGATTGCAT  (R) CTAGCAGCCTAAAGAACCAAGA | 590 | HpaII | SNP  (synonymous) |
| PMC_40 | 1H | 9411674 | C | T | (F) TGGTAGAGGAAGGAAACAAGAA  (R) GAACTTCACCCTCATCAATGTT | 696 | EcoRV | SNP  (synonymous) |
| PMC_41 | 1H | 9413572 | T | C | (F) GTTCACCCATGTTAGTTCCACT  (R) GTCGTTCTGTTACCTGGTTGAT | 516 | CviAII | SNP(INTRON) |
| PMC_42 | 1H | 9612082 | G | A | (F) TCCCTTTGATCCATATTATTCG  (R) TCCTTTGTTGAACATACCATGA | 648 | HpyCH4IV | SNP(UTR_3) |
| PMC_43 | 1H | 9612187 | A | G | (F) TCCCTTTGATCCATATTATTCG  (R) TCCTTTGTTGAACATACCATGA | 648 | MvaI | SNP(UTR_3) |
| PMC_44 | 1H | 9622061 | G | C | (F) TTATGAACTGAATGCTTGTTCG  (R) TGAAGATGATACATGGAGGACA | 564 | StuⅠ | SNP  (non-synonymous) |
| PMC_45 | 1H | 9622288 | G | T | (F) TGCAAGTCAACCTTGTCAAATA  (R) CAACACTGTTCTGCACATTTCT | 651 | BsrGI | SNP  (synonymous) |
| PMC_46 | 1H | 9622928 | G | A | (F) ACACGGAGAAATCTCATCAATC  (R) TTTGAGCCAAGCTAGTAACCTC | 574 | AluI | SNP(INTRON) |
| PMC_47 | 1H | 9623010 | G | A | (F) ACACGGAGAAATCTCATCAATC  (R) TTTGAGCCAAGCTAGTAACCTC | 574 | HpaII | SNP  (synonymous) |
| PMC_48 | 1H | 10099526 | T | G | (F) ACTTCTTCGACCGATTCTTCTT  (R) TCGGCATAGAGGGATGAATAC | 650 | DdeI | SNP  (synonymous) |
| PMC_49 | 1H | 10241361 | C | A | (F) TTAGCGTACGGAAACGTCTTAT  (R) TAACCTACATCTCCTGTGGAGG | 573 | HaeII | SNP(INTRON) |
| PMC_50 | 1H | 10241578 | G | C | (F) TGCGTTACTGTGTATCATTGGT  (R) TGAACACAAGTTATCGGAACTG | 610 | DdeI | SNP(INTRON) |
| PMC_51 | 1H | 10241627 | G | A | (F) ATGGAACGCTTTAATAGTGGTG  (R) ATCATCATTGTCCTTGAACACA | 511 | ScaI | SNP(INTRON) |
| PMC_52 | 1H | 10245800 | A | C | (F) GACCAGGGATCGTAGAGTGTAG  (R) TTGAGAATCAGCTTCATGTCAC | 633 | NcoI | SNP(INTRON) |
| PMC_53 | 1H | 10397843 | G | C | (F) GAACCTTGAGGGACATTGTCTA  (R) TCCATCTGGTTAGGAGAGAGAA | 626 | AgeI | SNP  (synonymous) |
| PMC_54 | 1H | 10536561 | A | G | (F) CAGCCTATCCCACATCATAGTT  (R) TATCTCAATGACTATCCCGACC | 396 | DdeI | SNP(INTRON) |
| PMC_55 | 1H | 10536902 | A | G | (F) GGTCGGGATAGTCATTGAGATA  (R) GGTGAAGATGTCTCTCTACCCA | 410 | BglⅡ | SNP  (non-synonymous) |
| PMC_56 | 1H | 10537350 | G | A | (F) CTCGAAGAGGTCTTTGGTATTG  (R) CTACATCGATGAGGAGACGAAT | 640 | MboI | SNP  (synonymous) |
| PMC_57 | 1H | 10549220 | T | C | (F) ATGCAGGAAATTCTGAAGAAGA  (R) CAGATCTGATGCAACAGTAGGA | 554 | CviQI | SNP(UTR_3) |
| PMC_58 | 1H | 10556034 | G | C | (F) GTGTTGTATATTGTGCCAATGC  (R) TCCACCATACGACTGATTGTTA | 590 | DdeI | SNP(INTRON) |
| PMC_59 | 1H | 10820379 | G | T | (F) ATCGTCTGCACTATTTATGGCT  (R) ATTTCTCTAGTTCTGCCTTCCC | 365 | SspI | SNP(INTRON) |
| PMC_60 | 1H | 10821141 | C | A | (F) TGATCCTGATGACCATGACTTA  (R) TTTGGTGTTGTAATGCTGAGAG | 473 | MboI | SNP  (synonymous) |
| PMC_61 | 1H | 10824528 | A | G | (F) ACTGCTAAGCGAATTATGTGGT  (R) ACTATGCAATACAGAAACGGCT | 564 | DdeI | SNP(UTR_3) |
| PMC_62 | 1H | 10848248 | T | G | (F) GTGATCCACCAATCATGGTACT  (R) GGAGAAGCAGCAGCAGAAG | 672 | AvaI | SNP  (synonymous) |
| PMC_63 | 1H | 11129348 | T | C | (F) GGATCACTGTTGATTTGACCTT  (R) CAGTCTGAGTTCCGATGATGTA | 405 | AseI | SNP(UTR_3) |
| PMC_64 | 1H | 11468936 | G | C | (F) CCTACTCAACTGCTCCATCTTC  (R) CAATACATGTGAACCTTCATCG | 598 | CviAII | SNP(INTRON) |
| PMC_65 | 1H | 11469002 | G | T | (F) CCTACTCAACTGCTCCATCTTC  (R) CAATACATGTGAACCTTCATCG | 598 | DdeI | SNP(INTRON) |
| PMC_66 | 1H | 11473647 | G | A | (F) TCTCTGATGGATTAAGGATTGC  (R) GCAAGACTCAGGGACAATTAAG | 315 | HpyCH4V | SNP(INTRON) |
| PMC_67 | 1H | 11474999 | G | A | (F) TGGGTGAAGGGATATCTATCTG  (R) CAAAGGATATCATCACACGAAA | 678 | CviQI | SNP(UTR_3) |
| PMC_68 | 1H | 11519355 | A | G | (F) ACATCATGGCGGACCTCTA  (R) CCGACGTTCATGGAGTAGAC | 670 | CviQI | SNP  (synonymous) |
| PMC_69 | 1H | 11547125 | G | A | (F) GTACATGCACCTGTCAACATTC  (R) TTTGCTTGGAAATGTCTTCTCT | 443 | SspI | SNP(INTRON) |
| PMC_70 | 1H | 11547475 | T | C | (F) GTACATGCACCTGTCAACATTC  (R) ATAACTTCTATCAGGGCTGCAA | 685 | HpyCH4IV | SNP  (synonymous) |
| PMC_71 | 1H | 11547580 | G | C | (F) TTGTGCATCATTAAGCAGAAAC  (R) ATAACTTCTATCAGGGCTGCAA | 460 | HinP1I | SNP  (non-synonymous) |
| PMC_72 | 1H | 11802894 | A | G | (F) GTGATTTGTCCCTTGTCTGTTT  (R) AAGAGAGAGCTAGCAGCAAAGA | 494 | HpyCH4Ⅴ | SNP  (non-synonymous) |
| PMC_73 | 1H | 11850259 | A | T | (F) ATGGGAAATGAAGAGAAAGGTT  (R) CTCTGAGAGCTTCATCTTTGCT | 373 | EcoRV | SNP(INTRON) |
| PMC_74 | 1H | 11851858 | C | T | (F) TTTCTCCAGCCTTAGTCATGTT  (R) AAAGAGCTAACTCACAACGCAT | 700 | DraI | SNP(INTRON) |
| PMC_75 | 1H | 11864697 | C | T | (F) GCTTCTTCGACATGGAGTACC  (R) GTCTGCTTTGACAGGTTGCT | 664 | ScaI | SNP  (non-synonymous) |
| PMC_76 | 1H | 11864741 | C | G | (F) GCTTCTTCGACATGGAGTACC  (R) GTCTGCTTTGACAGGTTGCT | 664 | NlaⅢ | SNP  (non-synonymous) |
| PMC_77 | 1H | 11882250 | T | C | (F) TGACTCCTCTAAGCACTTCCAT  (R) AAACAACAATGAGCTGTGAGTG | 366 | HpyCH4V | SNP(INTRON) |
| PMC_78 | 1H | 12107896 | T | C | (F) CTTGGTGATGTTAGGGTCAGAT  (R) AAGAGAGCAACCATTCTAGCTG | 631 | HpyCH4IV | SNP  (synonymous) |
| PMC_79 | 1H | 12108414 | ACCC | ACC | (F) CAACATAAGCACCCATACTGTG  (R) CAGTGTCAGGGATTAACCAAAT | 610 | HpyCH4V | InDel  (INTRON) |
| PMC_80 | 1H | 12613010 | A | G | (F) TATGGTCTACTTGGAGCCACTT  (R) AGATGCCGAACAATGCTACTAT | 496 | BstNⅠ | SNP  (non-synonymous) |
| PMC_81 | 1H | 12803406 | T | A | (F) TGCTCATCTTATTGCAGATGTC  (R) GCTCCAAATAATCCCAATGTAA | 494 | AluI | SNP(INTRON) |
| PMC_82 | 1H | 12803630 | A | G | (F) TGCTCATCTTATTGCAGATGTC  (R) GGAAATACTCCCGCTTAACTTT | 580 | HpaI | SNP(INTRON) |
| PMC_83 | 1H | 13208675 | A | G | (F) TTAGTGACTGACGTGTGGTTTC  (R) AGGGAAGAAGTTAGTCCTCACC | 665 | MvaI | SNP(INTRON) |
| PMC_84 | 1H | 13208883 | C | T | (F) ATAATTGGCAATACCACCTGAC  (R) TAAACTCCAGCTCCGACAGTAT | 587 | HinP1I | SNP(INTRON) |
| PMC_85 | 1H | 13210522 | T | G | (F) AGGAGATTTCATCAGTGTGCTT  (R) ACTCGATACATTGGATGCCTAC | 652 | DdeI | SNP(INTRON) |
| PMC_86 | 1H | 13368657 | G | GA | (F) GAGCTCGTCCTTTGTGTAAATC  (R) CCGTAGGTAGGTAACATCATCC | 549 | AluI | InDel  (INTRON) |
| PMC_87 | 1H | 13380333 | C | T | (F) TGCTGAGAGGATAAGGAGAGAG  (R) TGTAGGTGAACTCATCAACGAC | 635 | BalI | SNP  (non-synonymous) |
| PMC_88 | 1H | 13456122 | A | T | (F) ATCAATGTTCGCGCTACTTATT  (R) CCCAAGTCGATCGTTATTGTAT | 446 | CviAII | SNP(INTRON) |
| PMC_89 | 1H | 13456372 | C | G | (F) ATACAATAACGATCGACTTGGG  (R) CGTTTGTTTATGAAACATGGTG | 446 | HpyCH4V | SNP(INTRON) |
| PMC_90 | 1H | 13458387 | C | T | (F) CGTGTGTGTCCTGTTCTTTG  (R) ATGGGTTTCTACGGCTCTTC | 694 | CviQI | SNP(INTRON) |
| PMC_91 | 1H | 13865037 | C | T | (F) ACCACCTGTTAATCAGTTGTCC  (R) TCCTCATCGGAGTCTCATACTT | 684 | HpyCH4V | SNP(INTRON) |
| PMC_92 | 1H | 13953506 | C | G | (F) AGGTAGCCAGGAAGATGGAC  (R) CCTAATCAAACACGAACACTCA | 682 | DdeI | SNP(INTRON) |
| PMC_93 | 1H | 14098719 | T | G | (F) GTTATTATGGTGTGTTCCCAGG  (R) GTCGACCAAATCAGAAATTGTT | 555 | DdeI | SNP(UTR_3) |
| PMC_94 | 1H | 14099749 | T | TC | (F) TGAATTTCTTACGCATGACTTG  (R) TCGATAGTACATGCCTCCTCTT | 548 | CviAII | InDel(UTR_3) |
| PMC_95 | 1H | 14101542 | C | T | (F) AGGTGGCTTGAAAGATGAAATA  (R) AACTTCTGTCGGAACAGCTTTA | 507 | MboI | SNP  (non-synonymous) |
| PMC_96 | 1H | 14102525 | A | G | (F) CTTATGTCTACACAGCAACGGA  (R) CGCGAGTTTGTACAGTATTTGT | 559 | HpyCH4V | SNP(INTRON) |
| PMC_97 | 1H | 14147248 | G | T | (F) GCTGAAACAACTATTGGGTCTC  (R) AATTAGAGTGTCCACATGACCC | 433 | HpaI | SNP(INTRON) |
| PMC_98 | 1H | 14149897 | T | C | (F) CCATGTGATCCCAATTCTAGTT  (R) ACCGGTAGACTGATAACCAATG | 688 | MfeI | SNP(INTRON) |
| PMC_99 | 1H | 14150441 | G | A | (F) AGGATAGGGTACTCTCCTTTGG  (R) ACAACATGTAGATAGGGACGGA | 619 | MvaI | SNP(INTRON) |
| PMC_100 | 1H | 14151093 | C | T | (F) ATGAGGTCAGATAGTCACCCAC  (R) TCTGACTTAGGACAAAGCCAAT | 430 | CviAII | SNP(INTRON) |
| PMC_101 | 1H | 14151513 | C | G | (F) GCTCGTACTTTCTTTCTTCCAA  (R) TTAATGCGTACAGGTTCTGTTG | 635 | CviQI | SNP(INTRON) |
| PMC_102 | 1H | 14153934 | G | A | (F) TGTCAGATTCTAGGGTCGAAGT  (R) TTTCCCTTTATCCCTACTCCAT | 401 | TaqI | SNP(INTRON) |
| PMC_103 | 1H | 14155463 | T | A | (F) TTGCAAATTCGTGACATACATT  (R) TTGTTCAGGCTAGTTAGTCGGT | 466 | DdeI | SNP(INTRON) |
| PMC_104 | 1H | 14660785 | C | T | (F) GGTTGGCCATACACAGTTTAGT  (R) ACGTTGTAAGAACAAGGCAAGT | 416 | TaqI | SNP(INTRON) |
| PMC_105 | 1H | 14661960 | C | T | (F) CGCTGGTATCCTAGTAAATTGC  (R) TCTGTTCAGTATTTGTGCTTCG | 621 | BfaI | SNP(INTRON) |
| PMC_106 | 1H | 14667209 | G | C | (F) GTGGGTATCACTCAGGAATTGT  (R) AGGGAAGGAATGCTAGGATAAG | 556 | CviQI | SNP  (non-synonymous) |
| PMC_107 | 1H | 14713819 | T | C | (F) GTTAAATTCATGTTTCGGCAAT  (R) AGAGCACCCGTAATATTGTGAC | 464 | HpyCH4V | SNP(INTRON) |
| PMC_108 | 1H | 14715864 | C | A | (F) AATTGATGTCCAATTTCTTTGC  (R) CGGTATACCTGTCCAATCTCAT | 379 | HincII | SNP(INTRON) |
| PMC_109 | 1H | 14716423 | C | G | (F) GCGTATGAGATTGGACAGGTAT  (R) AAATTATGCATTTCATATGGGC | 425 | AvaII | SNP(INTRON) |
| PMC_110 | 1H | 14720414 | C | A | (F) TACATTACACTCCCTTCGACCT  (R) TTGTGCAACAATCTCTAAATGC | 576 | CviQI | SNP(UTR_3) |
| PMC_111 | 1H | 14726689 | G | A | (F) CTGTGATTAGTCCTGAGGAAGC  (R) GAACCAATTCCATCTCCAGTAA | 583 | Bsp1286I | SNP(synonymous) |
| PMC_112 | 1H | 14970596 | A | G | (F) AGCTCGTTGTCCTCTATGTTGT  (R) ATTCCTTTCTTGTGCTGGTAAA | 544 | MluI | SNP(synonymous) |
| PMC_113 | 1H | 15081617 | T | C | (F) GGAACAGGCTACAAACCTAGTG  (R) TGTTTGAAGTATTCACTCGACG | 613 | CviQI | SNP(INTRON) |
| PMC_114 | 1H | 15081836 | G | A | (F) GGAACAGGCTACAAACCTAGTG  (R) TGTTTGAAGTATTCACTCGACG | 613 | Hpy188Ⅰ | SNP  (non-synonymous) |
| PMC_115 | 1H | 15083727 | C | A | (F) CTGGTTTCTGACTCTGTCGTTT  (R) GGTAGATTCTCTATGCTCGTGG | 682 | BstUI | SNP(synonymous) |
| PMC_116 | 1H | 15084460 | G | A | (F) GGTGATTAACTTATGTGGGTGG  (R) GTCAGAATCTGGAAGACTGGAG | 305 | HaeIII | SNP  (synonymous) |
| PMC_117 | 1H | 15091857 | T | C | (F) AGTATTACATGGTGAAGTGGGC  (R) GGGAAATAGAAGAGCTGGAGAT | 672 | DdeI | SNP  (synonymous) |
| PMC_118 | 1H | 15092505 | G | A | (F) ATCTCCAGCTCTTCTATTTCCC  (R) AGATCCAGGTGATCCATGTAAG | 501 | HpyCH4V | SNP(INTRON) |
| PMC_119 | 1H | 15223620 | C | A | (F) TTTCTAACAAACCCTCTCTCCA  (R) AAACCTCAGCCTTCAATGTAAA | 662 | HpaII | SNP(INTRON) |
| PMC_120 | 1H | 15224424 | AGCAGGCG | AG | (F) TTAAGTCTCGACTTGAACGTGA  (R) CCAACCCAATATTTGTCACTCT | 646 | NheI | InDel  (CODON DEL) |
| PMC_121 | 1H | 15224929 | A | G | (F) CGCGTAATAGTTGATCAAGACA  (R) CTGCTTTATTTCCTTACGATGG | 690 | MwoⅠ | SNP  (non-synonymous) |
| PMC_122 | 1H | 15435920 | G | C | (F) ACAGAGTTTCTTCCTTCCTTCC  (R) TACAAGTATTGGAAGATTGGGC | 686 | SacII | SNP  (synonymous) |
| PMC_123 | 1H | 15436744 | G | A | (F) GTCCACAGTGACATTGACAAAC  (R) AATGGTGCGTTGATCTATTACC | 461 | AluI | SNP(INTRON) |
| PMC_124 | 1H | 15438324 | AAT | AATACTAT | (F) ACTTATGTTGCAGACTGGCTTT  (R) GGGTTACTGGTTTGTTGTGTCT | 395 | SspI | InDel  (INTRON) |
| PMC_125 | 1H | 15439149 | G | A | (F) CATATCGATGTCCTCTCCTGAT  (R) ATGTGACAGACATTTCCCTTTC | 557 | AluI | SNP(INTRON) |
| PMC_126 | 1H | 15440855 | G | C | (F) TTTGAAGTCTGAATATGTTGCG  (R) TCTGGGTATTTGGTCCAGTTAG | 633 | MvaI | SNP  (synonymous) |
| PMC_127 | 1H | 16855936 | G | T | (F) CAGTGTGTGAGGAAGACGC  (R) GCTTCTGTATCTTTCATGCTCC | 665 | CviAII | SNP  (synonymous) |
| PMC_128 | 1H | 19434762 | T | G | (F) GACCCTAGTTGCCATGTTAGAG  (R) CTACTTAAGAAATGTGCCTGCC | 526 | MboI | SNP(INTRON) |
| PMC_129 | 1H | 19594745 | C | T | (F) ACCACTACAAGCTACAGCCCTA  (R) TTCGGATAGATACTCAAGCGAT | 571 | HpyCH4Ⅴ | SNP  (non-synonymous) |
| PMC_130 | 1H | 19595703 | G | A | (F) GGAGTCTCCTCTCCAGAACTTT  (R) TATCAGTACCAAGGACCACACA | 473 | AgeI | SNP  (non-synonymous) |
| PMC_131 | 1H | 19821113 | T | C | (F) GTTGAAATGATCAGAAGGGTGT  (R) CATGTGTAGCATCAGGTGGA | 687 | MspA1I | SNP(INTRON) |
| PMC_132 | 1H | 19835099 | G | A | (F) CTCTGGCATTGTTAAGCTCTCT  (R) GCAACCAGTTTCAGGTACTCAT | 451 | HinfI | SNP  (synonymous) |
| PMC_133 | 1H | 19835685 | A | G | (F) GCATGGAGTGGTTCTCTATCAT  (R) CACGCCAATAAGGAGAAACTAA | 600 | HinfI | SNP(UTR_3) |
| PMC_134 | 1H | 20133661 | G | A | (F) TGAGCCTCTTATCCTTTGTGAT  (R) CCGGCTAATTACACATACCAGT | 484 | HpyCH4V | SNP(INTRON) |

**Supplementary Table 8** Predicted candidate genes and their functions associated with 21 non-synonymous single nucleotide polymorphism (SNP) markers. nSNP refers to a non-synonymous SNP.

| Marker | Position | Type | Gene ID | Description |
| --- | --- | --- | --- | --- |
| PMC_4 | 7842041 | nSNP | *HORVU.MOREX.r3.1HG0003700* | Polyadenylate-binding protein 2 |
| PMC_23 | 8870292 | nSNP | *HORVU.MOREX.r3.1HG0004130* | LINE-1 reverse transcriptase like |
| PMC_28 | 8938251 | nSNP | *HORVU.MOREX.r3.1HG0004220* | Cysteine proteinase |
| PMC_29 | 8951116 | nSNP | *HORVU.MOREX.r3.1HG0004230* | Elongation factor |
| PMC_30 | 8951914 | nSNP | *HORVU.MOREX.r3.1HG0004230* | Elongation factor |
| PMC_31 | 9312497 | nSNP | *HORVU.MOREX.r3.1HG0004540* | Pumilio-like protein |
| PMC_36 | 9330782 | nSNP | *HORVU.MOREX.r3.1HG0004560* | ERD (Early-responsive to dehydration stress) family protein |
| PMC_44 | 9622061 | nSNP | *HORVU.MOREX.r3.1HG0004890* | Kinetochore protein spc25 |
| PMC_55 | 10536764 | nSNP | *HORVU.MOREX.r3.1HG0005350* | Leucine-rich repeat protein kinase family protein putative |
| PMC_71 | 11547580 | nSNP | *HORVU.MOREX.r3.1HG0005620* | Pectin acetylesterase |
| PMC_72 | 11802894 | nSNP | *HORVU.MOREX.r3.1HG0005720* | Sensory neuron membrane protein 1 |
| PMC_75 | 11864697 | nSNP | *HORVU.MOREX.r3.1HG0005790* | Clathrin assembly protein putative expressed |
| PMC_76 | 11864741 | nSNP | *HORVU.MOREX.r3.1HG0005790* | Clathrin assembly protein putative expressed |
| PMC_80 | 12613010 | nSNP | *HORVU.MOREX.r3.1HG0006070* | Homeobox protein putative |
| PMC_87 | 13380333 | nSNP | *HORVU.MOREX.r3.1HG0006420* | Disease resistance protein |
| PMC_95 | 14101542 | nSNP | *HORVU.MOREX.r3.1HG0006660* | Inorganic pyrophosphatase family protein |
| PMC_106 | 14667209 | nSNP | *HORVU.MOREX.r3.1HG0007170* | Protoheme IX farnesyltransferase |
| PMC_114 | 15081836 | nSNP | *HORVU.MOREX.r3.1HG0007360* | Type I inositol-145-trisphosphate 5-phosphatase 1 |
| PMC_121 | 15224929 | nSNP | *HORVU.MOREX.r3.1HG0007460* | rRNA N-glycosidase |
| PMC_129 | 19594745 | nSNP | *HORVU.MOREX.r3.1HG0008380* | methyl-coenzyme M reductase II subunit gamma putative (DUF3741) |
| PMC_130 | 19595703 | nSNP | *HORVU.MOREX.r3.1HG0008380* | methyl-coenzyme M reductase II subunit gamma putative (DUF3741) |
